# Duration-modulated neural population dynamics in humans during BMI controls

**DOI:** 10.1038/s42003-026-10369-8

**Published:** 2026-06-26

**Authors:** Fei Yin, Charles Guan, Tyson Aflalo, Jorge Gamez, Kelsie Pejsa, Emily Rosario, Charles Liu, Ausaf Bari, Richard A. Andersen

**Affiliations:** 1 Division of Biology and Biological Engineering, California Institute of Technology, Pasadena, CA USA; 2 T&C Chen Brain-Machine Interface Center, California Institute of Technology, Pasadena, CA USA; 3 Research Institute, Casa Colina Hospital and Centers for Healthcare, Pomona, CA USA; 4 Department of Neurological Surgery, Keck School of Medicine of USC, Los Angeles, CA USA; 5 Neurorestoration Center, Keck School of Medicine of USC, Los Angeles, CA USA; 6 Department of Neurosurgery, David Geffen School of Medicine at UCLA, Los Angeles, CA USA

**Keywords:** Neural decoding, Brain-machine interface, Dynamical systems, Motor cortex

## Abstract

The motor cortex (MC) is often described as an autonomous dynamical system during movement execution. In an autonomous dynamical system, flexible movement generation depends on reconfiguring the initial conditions, which then unwind along known dynamics. An open question is whether these dynamics govern MC activity during brain-machine interface (BMI) control. We investigate MC activity during BMI cursor movements of multiple durations, ranging from hundreds of milliseconds to sustained over seconds. These durations are chosen to cover the range of movement durations necessary to control modern BMIs under varying precision levels. Movements share their MC initial condition with movements of different durations in the same direction. Long-duration movements sustain MC activity in a low-velocity steady state until each movement goal is reached. The difference across durations in MC population dynamics may be attributed to external inputs. Our results highlight the role of sustained inputs to MC during movement.

## Abstract (web summary)

Motor cortex population dynamics during brain-machine interface control reveal that movements of varied durations share initial conditions but require sustained external inputs to maintain prolonged low-velocity steady states.

## Introduction

The motor cortex (MC) plays a central role in volitional movements, but its precise mechanism is still debated^1^. MC studies from the 1980s to early 2000s largely focused on representational modeling (RM), identifying movement parameters that correlate directly with neural activity. Studies discovered a wide range of represented parameters, including movement direction, speed, force, muscle activity, and posture^2–8^. However, evidence for a literal parameter representation remains inconclusive. Multiple movement parameters were often represented simultaneously, and these representations shifted across task contexts^1,9,10^ or even within a single movement^11^, leading some researchers to suggest that this parameter search was misleading^1,10,12^.

The autonomous dynamical systems hypothesis (aDSH) emerged as a response to the limitations of RM in explaining MC activity. The aDSH states that the motor cortex comprises a pattern generator, where preparatory activity sets the initial condition and movement execution activity unfolds along predictable dynamics^13–19^. The aDSH has been demonstrated most comprehensively with able-bodied non-human primate subjects performing a reach tasks^13,20–22^. In these tasks, movement preparation initializes the neural state of the dorsal premotor (PMd) and motor cortex, which then determines quasi-oscillatory MC activity during reach execution^16^. Recurrent neural network models suggest that local recurrence could implement MC’s brief oscillations^16,18,23–25^, which provide a basis set for generating multiphasic muscle activity^13,18^.

Mathematically, a dynamical system can be described by a function *f* mapping the system state *x* and external inputs *u* to the instantaneous change in state $\frac{{{\rm{d}}}x}{{{\rm{d}}}t}$:

1 $$\frac{{{\rm{d}}}x}{{{\rm{d}}}t}=f(x(t),u(t))$$

where *t* indicates time. Frequently^14,26^, the system state is defined as the MC firing rate vector **r**, and *f* is constrained to be an additive linear function *h* that models local recurrence. The input **u** can include external stimuli and the firing rates of other brain areas. In this simplification, Eq. 1 can be written as:

2 $$\frac{{{\rm{d}}}{{\bf{r}}}}{{{\rm{d}}}t}=h({{\bf{r}}}(t))+{{\bf{u}}}(t)$$

The aDSH posits that the system (MC) is self-contained during the execution of prepared movements^27,28^. That is, *u* is negligible during movement execution, further simplifying Eq. 2 into:

3 $$\frac{{{\rm{d}}}{{\bf{r}}}}{{{\rm{d}}}t}=h({{\bf{r}}}(t))={{\bf{Ar}}}(t)+{{\bf{b}}}$$

where **A** is the linear dynamics matrix and **b** is an offset vector.

The aDSH explained several phenomena that had puzzled the RM framework. For example, neurons appear to change representational tuning with time because they generate a temporal basis rather than directly driving the output^14,17^. And apparent modulation by multiple movement parameters was not a literal parameter representation but rather a basis set for downstream muscle read-out.

Three related claims follow from the aDSH. First, because the local MC dynamics *h* are stable^29^ and external inputs **u** are hypothesized to be negligible, generating different neural trajectories **r**(*t*) is solely determined by specifying different initial conditions **r**(*t* = 0)^13,16,19^. Second, these dynamics cause movement; specifically, neural perturbations disrupt movement if and only if the perturbation alters the task dynamics subspace^24^. Third, under the aDSH, MC dynamics are agnostic to external input, including sensory feedback. In the absence of that feedback, the circuit cannot correct errors once a movement has begun. Since motor circuits are not noise-free systems, accurate execution depends on the intrinsic MC dynamics being robust to noise. Noise-robust autonomous dynamical systems exhibit low tangling (described in MC by ref. ^23^) (i.e., smooth flow fields) and low divergence (described in the supplementary motor area, but notably not in MC, by ref. ^30^).

A key open question is whether the same autonomous dynamical principles govern MC activity during brain-machine interface (BMI) control. Movement BMIs aim to restore movement to people with motor disabilities by decoding motor intent from neural activity^31–39^. BMI cursor control has long been considered analogous to able-bodied reaching^31,40–44^. Given the success of aDSH in explaining MC activity during able-bodied reaching, this analogy suggests that aDSH-based modeling could improve human BMI control^17,28^. Beyond planar reaching, aDSH-based models have already been extended to grasping, locomotion, and speech^27,45,46^, reinforcing their potential for diverse BMI applications.

However, the aDSH has also faced critiques, including alternative interpretations of the observed population structure^27,47^ and evidence that its principles may not generalize to more complex or interrupted movements, such as dexterous actions^26,48^ or stopping behaviors^30^. A core limitation for BMI applications is the assumption that external inputs are negligible. Compared to the able-bodied movements usually modeled by aDSH, BMI movements are controlled by a small fraction of neurons in MC, so BMI control is slower, less precise^49,50^, and prone to nonstationarities^51^. Consequently, high-performance BMI control benefits from rapid real-time feedback^49,52–55^. Moreover, proprioceptive signals reflected in MC during able-bodied movement^56^ are absent during BMI control. Behavioral dynamics and sensory inputs thus differ substantially between BMI control and able-bodied reaching.

Given these differences, an open question is how well the aDSH applies to BMI movements, particularly as dynamical systems modeling promises to improve neural decoding^17,28,29,57^. To examine whether MC activity during BMI control can be explained by autonomous dynamics alone or also reflects external drive, we designed cursor task variants that separately emphasized ballistic and sustained control. We found that ballistic and sustained movements shared similar MC initial conditions, yet sustained movements entered a prolonged low-velocity steady phase. This divergence suggests that, despite the absence of somatosensory and proprioceptive feedback, inputs from beyond local MC contribute to sustained activity, inconsistent with the aDSH.

## Results

### Intracortical brain-machine interface (BMI) cursor control

**Fig. 1:**
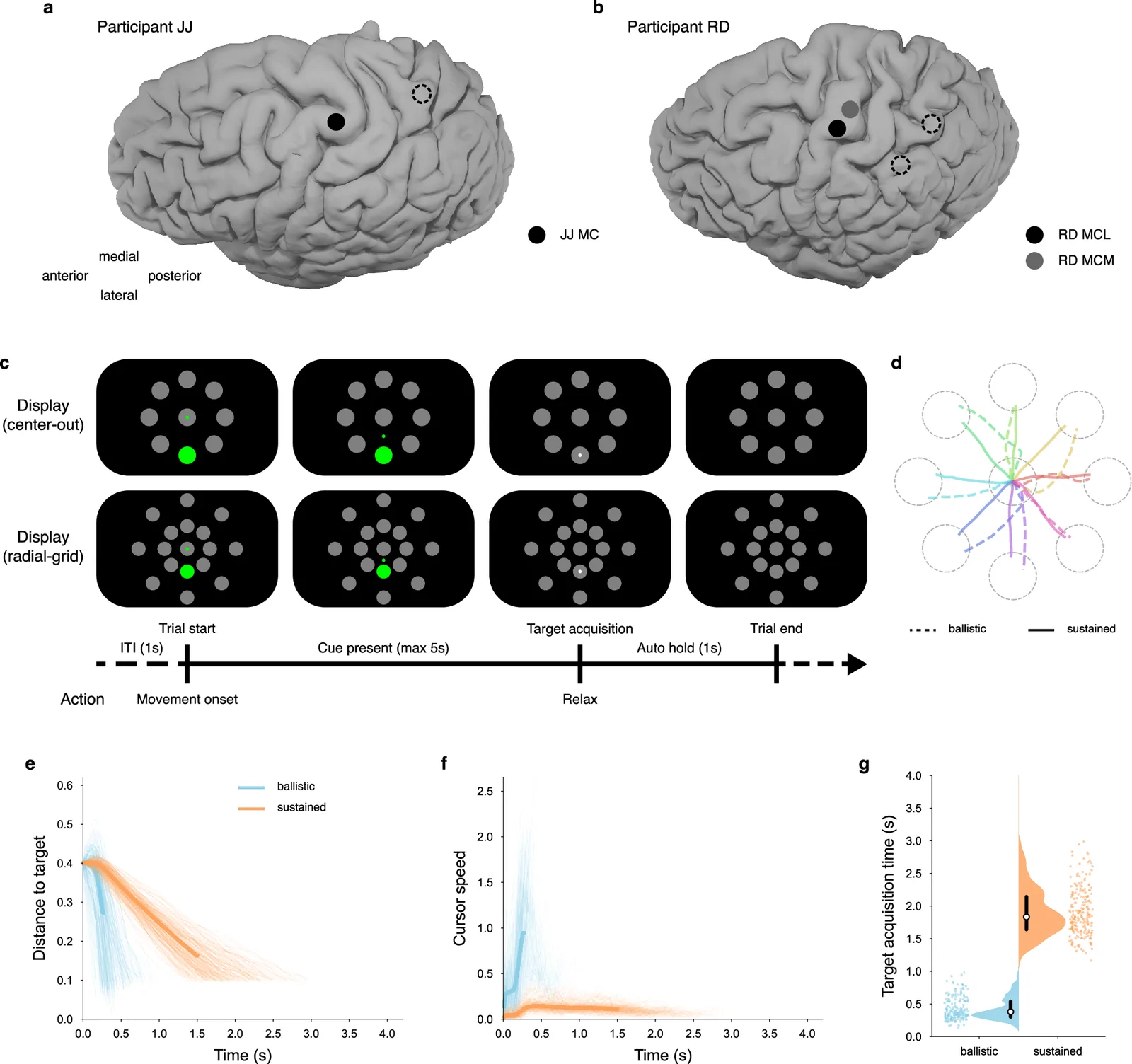
Locations of implanted arrays, trial phases, and behavioral data. **a** Implant locations in participant JJ. He was implanted with two 96-channel NeuroPort Utah electrode arrays (Blackrock Neurotech), one of which is in the left motor cortex (MC) near the hand knob area. The dashed circle indicates another implanted array not analyzed in this study. **b** Implant locations of participant RD. He was implanted with four 64-channel NeuroPort Utah electrode arrays, two of which are in the hand knob area of the left MC. Dashed circles indicate other implanted arrays not analyzed in this study. **c** Trial phases of the (ballistic vs. sustained) center-out and (near vs. far) radial-grid tasks. The participant sees a screen with targets radially arranged around a center reference. As a trial starts, one of the targets is cued (turns green), instructing the participant to move the cursor toward that target. When the cursor makes contact with the cued target, the participant relaxes, and the cursor is then automatically gravitated toward the center of that target. Consecutive trials are separated by a 1 s inter-trial interval (ITI). **d** Example single-trial cursor trajectories during ballistic and sustained conditions of the center-out task. The trajectories are color-coded by target. Targets are shown as circles with dashed lines. **e** Distance to target over time for participant JJ's center-out task trials (*n* = 180 ballistic and *n* = 207 sustained trials, pooled across 3 sessions). Each light-colored trace represents a trial. The dark colored traces represent condition averages. **f** Cursor speed over time for participant JJ's center-out task trials (same as those in **e**). Each light-colored trace represents a trial. The dark colored traces represent condition averages. **g** Target acquisition times for participant JJ's center-out task trials (same as those in **e**). Each half-violin shows the condition-specific kernel density estimate of the distribution across all trials. The central vertical bar spans the interquartile range (25th–75th percentile); the white circle indicates the median. Individual trials are shown as dots.

We recorded neural activity from microelectrode arrays implanted in participants JJ’s and RD’s hand knob of the left MC (Fig. 1a, b) while they completed various 2-D BMI cursor tasks. Both participants lost hand control after cervical spinal cord injuries, but attempted to move their effectors as though controlling an analog game controller stick. Using data from an attempted-movement calibration task, we trained a closed-loop decoder to predict cursor velocity from neural firing rates (see “Methods”). We use the terms “BMI control” and “movement” interchangeably. Although all arrays were used for online BMI control, we only analyzed MC recordings to investigate the aDSH.

### Ballistic and sustained BMI movements

The aDSH is typically studied in non-human primates performing ballistic arm reaches, with movement durations on the order of 400 ms^13,16^, but comparably precise BMI movements can take several seconds^50^. BMI-useful models of MC activity should apply to movements of both ballistic and sustained timescales. To replicate ballistic and sustained reaches, the participants performed the center-out BMI task (Fig. 1c) under two decoder gain parameters. For ballistic reaches, the decoder gain was set so that movements completed within roughly 500 ms. For sustained reaches, we decreased the decoder gain to require sustained movement of more than 1.5 s. We wanted to investigate reaches analogous to well-practiced, able-bodied reaching, but BMI control is imprecise at the high decoder gains^50^ used in the ballistic condition. Therefore, we partially assisted ballistic cursor control (see “Methods”); the assistance level was kept small to ensure that cursor movement was still primarily driven by neural activity. Furthermore, participants were not required to hold the cursor at the target. They were instructed to relax when they reached the target. Ballistic and sustained reaches were grouped into respective trial blocks. Trial blocks were interleaved.

Under both gain parameters, the participants acquired the targets consistently (Fig. 1d–g and Video 1). As designed, ballistic (BMI) movement trials took a roughly similar amount of time as previous non-human primate studies^11,13^ (acquisition time, participant JJ sessions: 0.43 s ± SD 0.17 s; participant RD sessions: 0.79 s ± SD 0.16 s). As designed, sustained movement trials were substantially longer (acquisition time, participant JJ sessions: 1.91 s ± SD 0.37 s; participant RD sessions: 2.07 s ± SD 0.41 s; 95% confidence interval of mean difference across conditions (ballistic vs. sustained): [1.42, 1.53] s for participant JJ sessions, [1.24, 1.32] s for participant RD sessions). These patterns were consistent across sessions (Supplementary Fig. 1a–c).

Cursor speed profiles reflect the task structure (Fig. 1f and Supplementary Fig. 1b). After trial onset, an initial visual delay period is evident before the participant takes full control of the cursor to approach the target. This is consistent with the distance-to-target traces where the distance initially increases during some trials (Fig. 1e and Supplementary Fig. 1a). Ballistic trials then reach higher speeds, with many trials completing shortly after reaching maximum speed, while remaining trials continue toward the target at reduced speed. Sustained trials exhibit lower overall speeds with a more gradual reduction as the cursor approaches the target.

### Comparing ballistic and sustained BMI movements reveals multiphasic dynamics

**Fig. 2:**
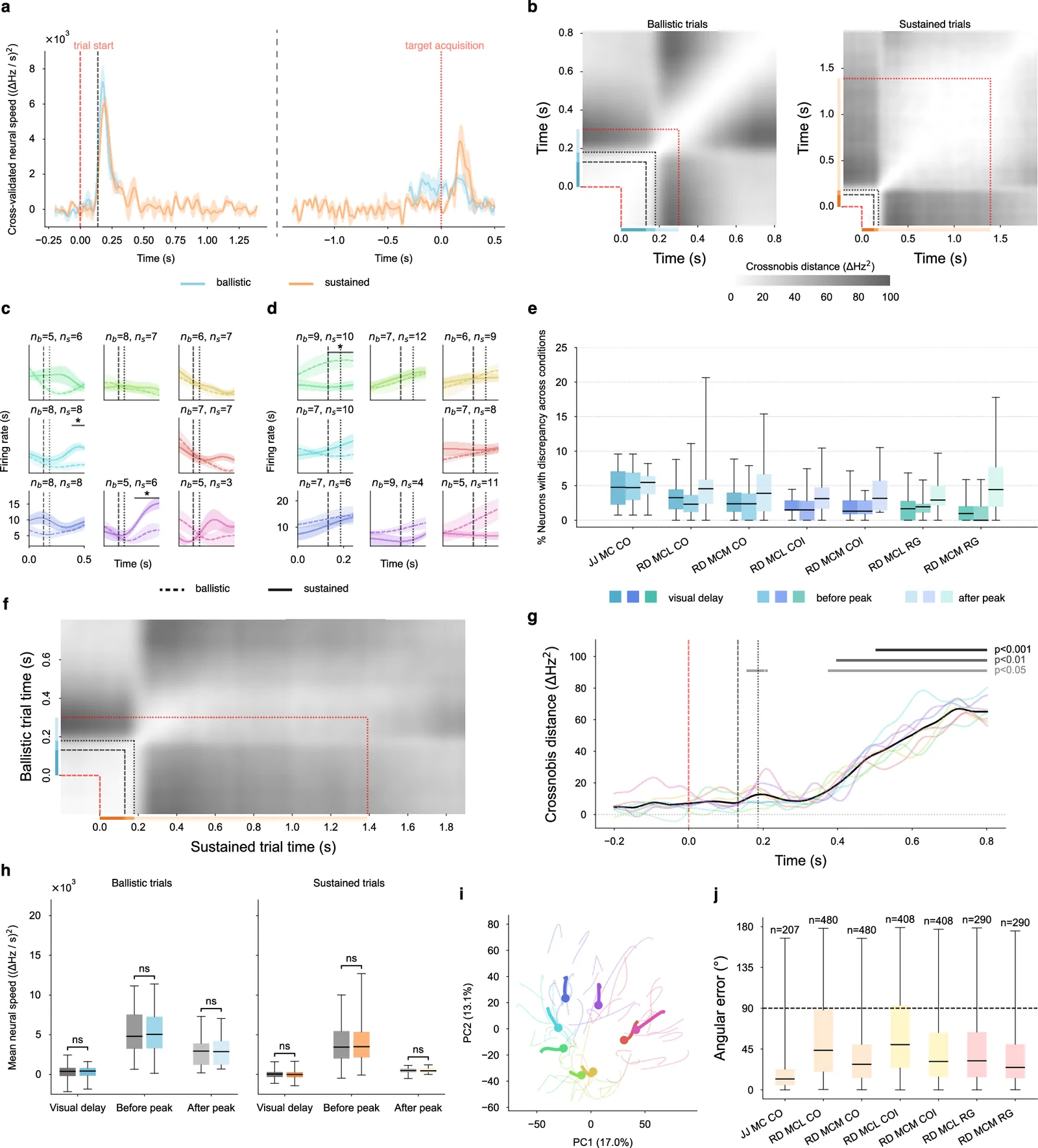
Multiphasic neural dynamics during BMI movements and cross-condition discrepancy. **a** Cross-validated neural speed during ballistic (*n* = 162) and sustained (*n* = 186) trials in participant JJ's center-out sessions. Trials of each condition are truncated to the shortest trial duration observed for that condition. Error bands show 95% confidence intervals across trials. Left: trials are aligned to the start time marked by the red dashed line. Black dashed line (left) marks the peak onset time, i.e., the time at which neural speed first rises above baseline (see “Methods”). Data includes 200 ms before trial start. Right: trials are aligned to the target acquisition time marked by the red dotted line. Data includes 500 ms after target acquisition. **b** Crossnobis distance across time for ballistic (left; *n* = 162) and sustained (right; *n* = 186) trials in participant JJ's center-out sessions. Data includes 200 ms before trial start and 500 ms after target acquisition. Trials of each condition are truncated to the shortest trial duration observed for that condition. Red dashed lines mark the trial start time. Red dotted lines mark the target acquisition time of the shortest trial of each condition. Black dashed lines and dotted lines mark the peak onset times and peak times (see “Methods”), respectively. Horizontal and vertical bars beside axis ticks mark phases within each trial. Lighter to darker shades of each color correspond to visual delay (see “Methods”), before-peak, and after-peak windows, respectively. **c**, **d** Example single neuron responses to each target in participant JJ's center-out sessions. Each color represents a target. Error bands show SEMs across trials. The number of ballistic (*n*_*b*_) and sustained (*n*_*s*_) trials per target is indicated above each subplot. Dashed and dotted vertical lines mark the peak onset times and peak times, respectively. Significant differences between ballistic and sustained trials are marked (Mann–Whitney *U* test with false discovery rate correction, *p < 0.05). **e** Percent of neurons with significant cross-condition discrepancy across targets in all sessions from both participants. Blues, indigos, and greens denote the gain-scaling (*n* = 3 participant JJ sessions; *n* = 6 participant RD sessions), interleaved gain-scaling (*n* = 3 participant RD sessions), and distance-scaling (*n* = 3 participant RD sessions) tasks, respectively. Lighter to darker shades of each color correspond to the visual delay, before-peak, and after-peak windows, respectively. **f** Crossnobis distance across time between ballistic (y-axis; *n* = 162) and sustained (x-axis; *n* = 186) trials in participant JJ's center-out sessions. Notation and conventions are the same as in (**b**). **g** Cross-condition crossnobis distance along the diagonal (same time point across conditions) for ballistic (*n* = 162) versus sustained (*n* = 186) trials. The black trace shows the mean across sessions and target directions. Thin colored traces show per-target averages. Horizontal bars above the traces indicate time bins where the crossnobis distance is significantly greater than the pre-movement baseline (Mann–Whitney *U* test, FDR-corrected; bar shading indicates *p* < 0.05, *p* < 0.01, and *p* < 0.001). Vertical lines follow the conventions in (**b**). **h** Robustness check for within-condition crossnobis distance after removing neurons with significant firing rate differences between ballistic and sustained trials. For each neuron and target direction, a Mann–Whitney *U* test was applied at each time bin spanning the visual delay, before-peak, and after-peak epochs; FDR correction was applied jointly across all movement-epoch bins. Neurons significant in any movement epoch or target direction were removed. Box plots show neural speed averaged within each movement epoch (visual delay, before-peak, after-peak) before (gray) and after (condition color) neuron removal. Each observation is one per-target crossnobis value pooled across sessions. Boxes span the interquartile range. Whiskers extend to the minimum and maximum. Horizontal lines indicate the median. Brackets indicate paired Wilcoxon signed-rank tests between the original and reduced neural populations (*n* = 24 per-target observations: 8 targets × 3 sessions). **i** Trajectories of the top 2 principal components of motor cortex (MC) population activity during the final 300 ms before target acquisition. Each thin trace represents a single trial colored by target direction. Bold traces show target averages. Filled circles mark the trajectory endpoint (mean firing rate at target acquisition). Variance explained by each PC is indicated on axis labels. Data shows sustained trials (*n* = 52) during one of participant JJ's sessions. **j** Target direction decoding from the final 300 ms of neural activity in sustained trials, summarized across participants and tasks. For each trial, the time-averaged population firing rate vector was used to decode the continuous target angle via L2-regularized linear regression. The target angle was encoded as ($\cos \theta ,\sin \theta$) to handle circular periodicity, and the predicted angle was recovered via arctan2. Decoding accuracy was estimated using 5-fold cross-validation. Box plots show the distribution of absolute angular error (degrees) across trials pooled across sessions within each participant-region-task group. Boxes span the interquartile range, whiskers extend to the minimum and maximum, and horizontal lines indicate the median. The dashed line indicates the chance level (90^∘^, the expected mean absolute error for a uniformly random predictor). The number of trials is indicated above each box.

We computed the cross-validated neural speed (see “Methods”) for MC activity during ballistic and sustained BMI movements (Fig. 2a and Supplementary Fig. 2a, b). Cross-validated neural speed rose sharply during movement onset (participant JJ: mean Δ = 8.42 kHz^2^ s^−1^, 95% CI [7.31, 9.53] kHz^2^ s^−1^, *p* < 0.001, Cohen’s *d* = 2.20; participant RD: mean Δ = 7.13 kHz^2^ s^−1^, 95% CI [6.46, 7.80] kHz^2^ s^−1^, *p* < 0.001, Cohen’s *d* = 1.52; Wilcoxon signed-rank test) but then dropped by about 92% and stabilized near ITI baseline (participant JJ: mean Δ = 0.74 kHz^2^ s^−1^, 95% CI [0.16, 1.32] kHz^2^ s^−1^, *p* = 0.014, Cohen’s *d* = 0.37; participant RD: mean Δ = 0.51 kHz^2^ s^−1^, 95% CI [0.25, 0.77] kHz^2^ s^−1^, *p* < 0.001, Cohen’s *d* = 0.28; Wilcoxon signed-rank test), even as the movements continued. This indicates that the neural activity maintained a constant, stationary representation of intent while sustaining the BMI movement. We will refer to the movement onset with high neural speed as the transient phase and the subsequent movement continuation with near-baseline neural speed as the steady phase. Ballistic trials completed earlier than sustained trials, resulting in a less prominent steady phase (participant JJ: mean durations 0.316 s vs. 1.833 s, Δ = − 1.517 s, 95% CI [−1.580,−1.455] s, *p* < 0.001, rank biserial *r* = 1.000; participant RD: 0.494 s vs. 1.719 s, Δ = − 1.225 s, 95% CI [−1.258,−1.192] s, *p* < 0.001, rank biserial *r* = 0.981; Mann–Whitney *U* test). The initial peak in neural speed occurred at similar times in ballistic and sustained trials (participant JJ: Δ = − 0.021 s, 95% CI [−0.038, −0.003] s, *p* = 0.016, rank biserial *r* = 0.429; participant RD: Δ = − 0.040 s, 95% CI [−0.076, −0.004] s, *p* = 0.099, rank biserial *r* = 0.143; Mann–Whitney *U* test) (Supplementary Fig. 2m, n). Comparing the two participants, the initial peak occurred about 184 ms earlier (Δ = − 0.184 s, 95% CI [−0.204, −0.164] s; *p* < 0.001, Mann–Whitney *U* test; rank biserial *r* = 0.963) in participant JJ than in participant RD under both conditions (Supplementary Fig. 2m, n). This is likely due to the difference in reaction time between the two participants. In the acquisition-aligned traces (Fig. 2a, right), a secondary deflection in neural speed is visible near target contact, most prominently in participant JJ. This reflects movement offset—the transition from active control to relaxation. This is consistent with JJ’s more binary onset-offset motor imagery strategy (see “Methods”). Participant RD’s graded, position-like strategy produces a less abrupt offset (Supplementary Fig. 2a, b).

The crossnobis distance (see “Methods”) of MC activity between all pairs of time indices during BMI movements (Fig. 2b and Supplementary Fig. 2g, h) paints a more holistic picture. In particular, the crossnobis distance between the pre-movement period (ITI and visual delay period) and the steady phase is large, indicating that they do not share tuning profiles. Furthermore, the crossnobis distance is relatively smaller within the steady phase. In other words, it is not just that the participants were inactive during the movement or that coding is purely phasic. Instead, their neural activities were actively sustained during that time. To directly confirm that steady-phase activity carries goal-directed information rather than reflecting nonspecific arousal or neural idling, we examined the final 300 ms of sustained trials before target acquisition. Principal component analysis (PCA) of steady-phase MC activity reveals target-separable structure: trajectories cluster by target direction in the top two principal components (Fig. 2i). A linear discriminant analysis classifier trained on time-averaged firing rate vectors from this window decoded target direction well above chance across participants and tasks (Fig. 2j). These results demonstrate that MC activity during the steady phase maintains a structured, target-specific representation of motor intent throughout sustained BMI movements. Taken together, when a BMI user attempts to move and sustain motor intent, MC activity appears to consist of an initial dynamic phase and a subsequent steady phase until the target is reached.

### Mid-movement cross-condition discrepancies in single-neuron and population activity suggest input-driven dynamics

Task condition modulated the neural response. The aDSH attributes variation in movement to differences in the pre-movement neural state. However, if differences between conditions emerge after movements have begun, it would suggest that inputs to MC are no longer negligible, contradicting the aDSH. We refer to such differences as mid-movement cross-condition discrepancies. We examined whether individual neurons showed cross-condition discrepancies during movement. When tested per target direction, approximately 5% of neurons showed significant firing rate differences between ballistic and sustained trials during the steady phase (Fig. 2c–e). However, the identity of these neurons varied across targets— when we asked whether a neuron showed a cross-condition discrepancy for any target, the fraction rose to upward of 40% (Supplementary Fig. 3, ALL columns). This suggests that mid-movement input-related modulation is broadly distributed across the recorded population rather than concentrated in a specialized subpopulation.

To test whether cross-condition discrepancies are evident at the population level, we computed the crossnobis distance between ballistic and sustained neural states at matched time points (Fig. 2f, g, and Supplementary Fig. 4). Cross-condition crossnobis distance was low during the transient phase but rose significantly above the pre-movement baseline during the steady phase across target directions, sessions, and participants (Mann–Whitney *U* test, FDR-corrected; Fig. 2g). As a robustness check, we removed all neurons that showed significant firing rate differences between conditions in any epoch or target direction and recomputed within-condition neural speed. For participant JJ, the changes were not significant (Fig. 2h); for participant RD, some epoch-level differences reached significance, but the overall pattern was preserved, with neural speed changing by at most 20% (Supplementary Fig. 5). This confirms that the population-level cross-condition discrepancy is not driven by the subset of individually significant neurons, consistent with a broadly distributed input signal.

Similar patterns are present on the population level. We fit a linear dynamical system (LDS), a proxy implementation of the aDSH, to ballistic and sustained trials jointly. LDS models the data as:

4 $${{{\bf{x}}}}_{t+1}={{{\bf{Ax}}}}_{t}+{{\bf{b}}}+{{{\bf{v}}}}_{t}$$

5 $${{{\bf{y}}}}_{t}={{{\bf{Cx}}}}_{t}+{{\bf{d}}}+{{{\bf{w}}}}_{t}$$

where **A** and **b** are the dynamics matrix and bias, **C** and **d** are the emission matrix and bias, **v**_*t*_ and **w**_*t*_ are zero mean Gaussian noise terms, **y**_*t*_ is the observation (MC firing rates), and **x**_*t*_ is the latent state. When we train an LDS with some high-dimensional sequential data, we effectively reduce the dimensionality to the number of latent states. Note that **A** and **b** do not change with time.

**Fig. 3:**
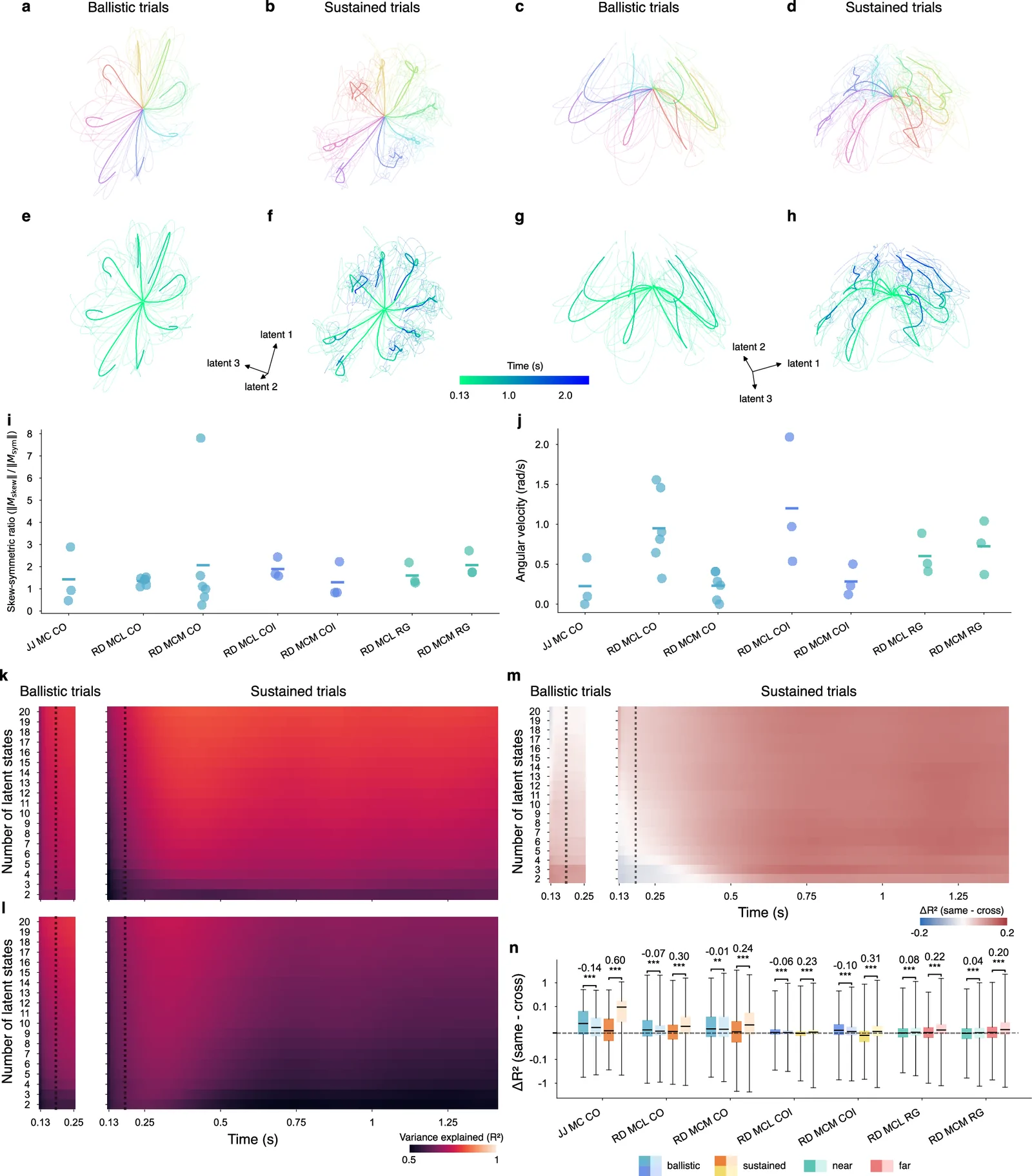
Task condition modulated the neural response in LDS latent spaces, showing additional evidence of inputs. Latent neural trajectories from fitting a 3-dimensional linear dynamical system (LDS) to ballistic (**a**; *n* = 52) and sustained (**b**; *n* = 52) trials jointly. Colors represent different targets. Thin trajectories show single trials. Thick trajectories show target averages after time-resampling. **c**, **d** The same LDS latent neural trajectories as in (**a**, **b**) but from a different viewing angle. **e**, **f** Latent neural trajectories color-coded by time since the start of trials. Thin trajectories show single trials. Thick trajectories show target averages after time-resampling. **g**, **h** Same as (**e**, **f**), but from a different viewing angle and without single trials. **i** Skew-symmetric ratio (see “Methods”) of the fitted LDS dynamics matrix, quantifying the relative strength of rotational versus non-rotational dynamics. Individual dots represent individual recording sessions. There are *n* = 3 center-out sessions for participant JJ and *n* = 6 center-out, *n* = 3 interleaved center-out, and *n* = 3 radial-grid sessions for participant RD. Horizontal lines indicate the cross-session mean. Because the model was trained jointly on sustained and ballistic trials, a single dynamics matrix governs both conditions, and results are combined across conditions. **j** Dominant angular velocity (see “Methods”) of the fitted LDS dynamics, reflecting the fastest rotational frequency in the latent state evolution. Visualization follows the standards of (**i**). **k** Cross-validated inference performance of LDS models on ballistic (left; *n* = 162) and sustained (right; *n* = 186) trials of participant JJ's center-out sessions. The models are trained and tested on trials of the same condition. Data are averaged across cross-validation folds and sessions. The width of each heatmap is proportional to trial duration. Dotted vertical line indicates peak time, where the neural speed is highest (see “Methods”). **l** Same as (**k**), but for cross-condition models where training and testing are performed on trials of opposite conditions. i.e., ballistic trials are inferred using models trained on sustained trials, and vice versa. **m** Difference in inference performance between same-condition (**k**) and cross-condition (**l**) models for ballistic (left) and sustained (right) trials of participant JJ's center-out sessions. Positive values (red) indicate that the same-condition model forecasts neural dynamics more accurately than the cross-condition model; negative values (blue) indicate the reverse. **n** Difference in inference performance between same-condition and cross-condition models is partitioned into transient and steady phases of movements. Data are aggregated across time bins, latent dimensions (2–20), cross-validation folds, and sessions. For each participant, brain area, and task combination (x-axis), four boxes show the distribution of per-time-bin Δ*R*^2^ values: the two left boxes correspond to the first condition (ballistic or near), and the two right boxes correspond to the second condition (sustained or far), with darker shading indicating the transient phase (before peak time) and lighter shading the steady phase (after peak time). There are *n* = 162 ballistic and *n* = 186 sustained trials for participant JJ's center-out task, *n* = 426 ballistic and *n* = 429 sustained trials for participant RD's center-out task, *n* = 476 ballistic and *n* = 366 sustained trials for participant RD's interleaved center-out task, and *n* = 252 near and *n* = 260 far trials for participant RD's radial-grid task. Box plots show medians and interquartile ranges; whiskers extend to the minimum and maximum. Brackets denote two-sided Mann–Whitney *U* tests comparing transient versus steady distributions; effect sizes are reported as rank-biserial *r*. Dashed horizontal line marks Δ*R*^2^ = 0. The Y-axis is displayed on a symmetric logarithmic scale (linear within ±0.1, logarithmic beyond).

LDS produced latent trajectories that encode the radial structure of the center-out task (Fig. 3a–d). Time-coloring these trajectories shows that ballistic and sustained movements begin on comparable regions of a low–dimensional manifold, yet diverge in their temporal evolution: ballistic paths skim the outer “ring” (steady phase) and terminate quickly, whereas sustained paths linger on the same ring for the majority of the movement (Fig. 3e–h and Supplementary Fig. 6a–n). Such unequal dwell times, coupled with the cross-condition discrepancies quantified earlier (Fig. 2f, g, and Supplementary Fig. 4), suggest that a single time-invariant flow field may not fully account for the observed condition-dependent trajectories.

When we compare same-condition with cross-condition LDS inference across latent dimensionalities and time, same-condition models generally outperform cross–condition models, particularly in the steady phase (Fig. 3k–m and Supplementary Fig. 7). At low latent dimensionalities during the transient phase, cross–condition models occasionally match or slightly exceed same-condition performance (negative Δ*R*^2^ values in Fig. 3m). This occurs because ballistic trials, being shorter, contribute proportionally more transient-phase data to their training set; the resulting models therefore capture transient dynamics well and can generalize to the early, dynamically similar portion of sustained trials. As the number of latent states increases and the model grows more expressive, the advantage of same-condition inference diminishes for ballistic trials but persists for sustained trials, especially in their steady phase (Fig. 3n). Multiple factors may contribute to this asymmetry: the limited amount of steady-phase training data in the brief ballistic trials, genuine condition-specific dynamics in the steady phase, or the possibility that ballistic trials terminate before fully transitioning to a distinct steady-state regime. The LDS partition alone cannot disambiguate these factors. Nonetheless, the pattern is consistent with the broader evidence that condition-specific structure emerges predominantly after the initial transient, as shown by cross-condition crossnobis divergence over continuous time (Fig. 2f, g) and single-neuron discrepancies (Fig. 2e).

To formally quantify rotational structure in the LDS trajectories, we computed the skew-symmetric ratio and dominant angular velocity of the fitted dynamics matrix (see “Methods”). Because LDS fits a single time-invariant dynamics matrix, these metrics characterize the overall rotational content of MC dynamics across the entire movement (Fig. 3i, j). The skew-symmetric ratio and dominant angular velocity varied across sessions, participants, cortical subregions, and tasks, indicating that the degree of rotational structure in the fitted dynamics is not a fixed property of MC but depends on recording and task context. However, the time-invariant nature of LDS does not allow these dynamics-dependent metrics to be decomposed by phase. More broadly, the linear LDS offers only a single, time–invariant flow field shared across all conditions. Within this model class, any cross-condition discrepancy not explained by initial conditions would imply the influence of external inputs. However, a nonlinear autonomous system could produce different local dynamics in different regions of state space, potentially accounting for the cross-condition discrepancies without invoking external inputs. To test this possibility, we next fit a recurrent switching linear dynamical system (rSLDS) that allows piece-wise linear dynamics while still remaining autonomous.

### Modeling multiphasic dynamics with switching linear dynamical systems

Recurrent switching linear dynamical systems (rSLDS)^58^ (see “Methods”) provide a way to adjudicate between nonlinear autonomy and external inputs. If nonlinearity alone explains the condition-dependent differences observed in the LDS analysis, an rSLDS should require three or more discrete states: one for the transient phase and at least two distinct steady-phase states (for ballistic and sustained trials, respectively) to fit the data optimally. Conversely, if a two-state model suffices, the residual cross-condition discrepancies are consistent with modulation by external inputs.

We therefore trained rSLDS models on the same MC firing rates, sweeping the number of continuous states (C) and discrete states (D). An rSLDS is defined as:

6 $${{{\bf{x}}}}_{t+1}={{{\bf{A}}}}_{{z}_{t}}{{{\bf{x}}}}_{t}+{{{\bf{b}}}}_{{z}_{t}}+{{{\bf{v}}}}_{t}$$

7 $${{{\bf{y}}}}_{t}={{\bf{C}}}{{{\bf{x}}}}_{t}+{{\bf{d}}}+{{{\bf{w}}}}_{t}$$

Similar to an LDS, ${{{\bf{A}}}}_{{z}_{t}}$ and ${{{\bf{b}}}}_{{z}_{t}}$ are the dynamics matrix and bias, **C** and **d** are the emission matrix and bias, **v**_*t*_ and **w**_*t*_ are zero-mean Gaussian noise terms, **y**_*t*_ is the observation (MC firing rates), and **x**_*t*_ is the latent state. However, with the addition of *z*_*t*_, the discrete state that varies with time, an rSLDS allows the latent dynamics to switch between *z* sets of dynamics parameters at data-driven switch times; the emission parameters are shared across all discrete states. When *z* equals 1, rSLDS becomes equivalent to an LDS. For clarity, we will refer to “latent state” as “continuous state” to distinguish it from “discrete state” hereafter.

**Fig. 4:**
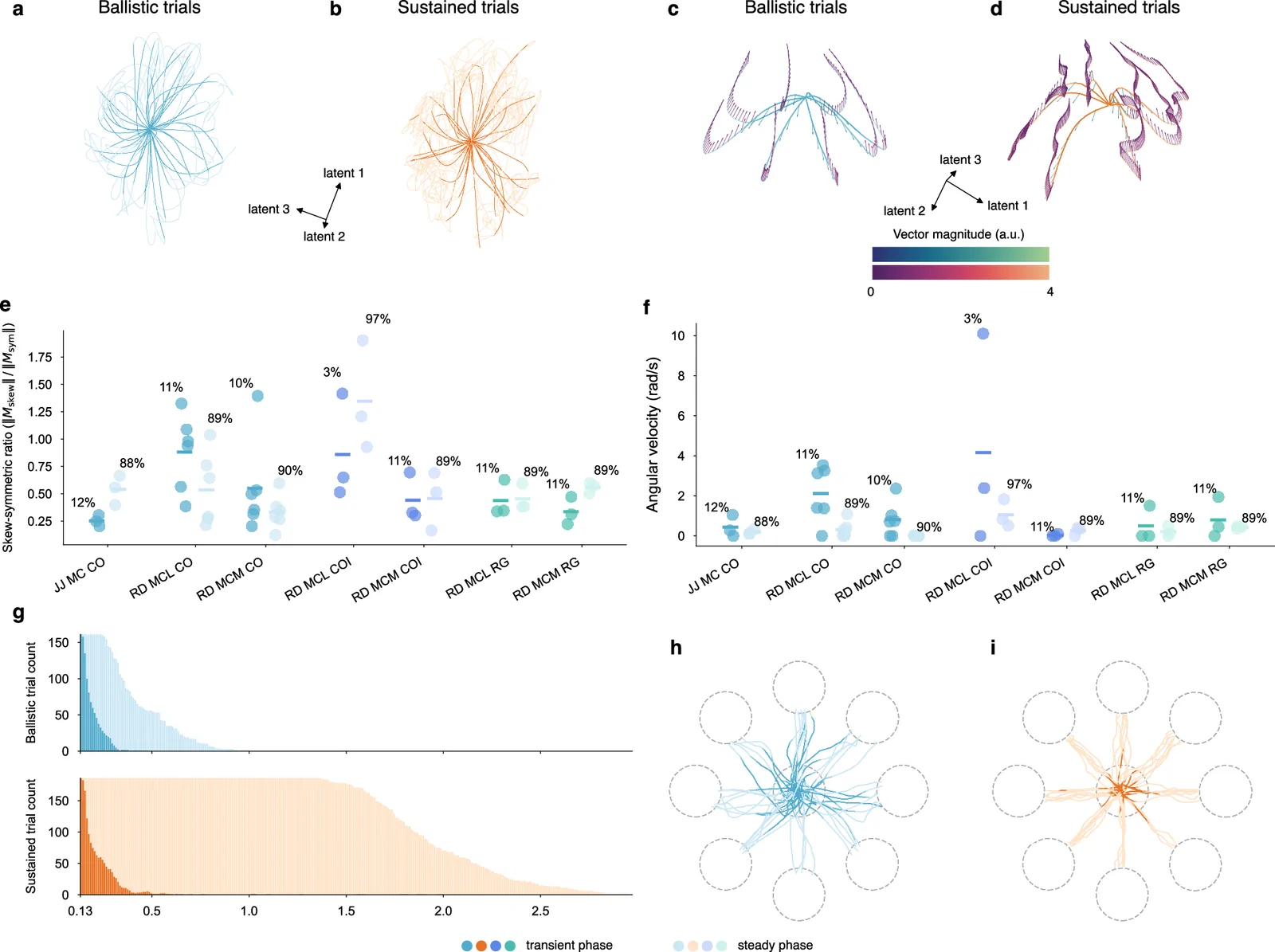
Time and discrete state visualization in rSLDS (C3D2) results. Latent neural trajectories from fitting a 3-dimensional 2-discrete-state recurrent switching linear dynamical system (rSLDS) to ballistic (**a**; *n* = 52) and sustained (**b**; *n* = 52) trials jointly. The trajectories are color-coded by discrete states. Each trajectory represents a trial. **c**, **d**, Same as (**a**, **b**), but from a different viewing angle and without single trials. In addition, flow field vectors learned by rSLDS are overlaid on the trajectories. Transient phase and steady phase flow field vectors are represented by cold and warm color maps, respectively. **e** Skew-symmetric ratio (see “Methods”) of the fitted rSLDS dynamics matrices, quantifying the relative strength of rotational versus non-rotational dynamics for each discrete state. Discrete states are ordered by the temporal center of mass and classified as transient (early) or steady-state (late). Fractional occupancy is annotated above each group. Individual dots represent individual sessions. There are *n* = 3 center-out sessions for participant JJ and *n* = 6 center-out, *n* = 3 interleaved center-out, and *n* = 3 radial-grid sessions for participant RD. Horizontal lines indicate the cross-session mean. Because the model was trained jointly on sustained and ballistic trials, the dynamics matrices are shared across conditions, and results are combined. **f** Dominant angular velocity (see “Methods”) of the fitted rSLDS dynamics, reflecting the fastest rotational frequency in the latent state evolution for each discrete state. Visualization follows the conventions of (**e**). **g** Trial counts of rSLDS (C3D2) discrete states over time grouped by task condition. *n* = 162 ballistic and *n* = 186 sustained trials from participant JJ's center-out sessions (*n* = 3) are shown. Behavioral cursor trajectories during ballistic (**h**; *n* = 52) and sustained (**i**; *n* = 52) trials overlaid with discrete states. Each trajectory represents a trial. Circles with dashed lines represent the targets.

Fitting an rSLDS with three continuous states and two discrete states (C3D2) yields trajectories (Fig. 4a–d) that closely mirror the LDS solution (Fig. 3a–d). When colored by discrete state, the turning of each rSLDS trajectory aligns with the transient-to-steady transition identified earlier (Fig. 4a, b). Overlaying target-averaged trajectories with the learned flow-field vectors reveals that steady-phase vectors have smaller magnitudes than transient-phase vectors under both conditions, indicating that the population enters a slow-moving attractor once the movement passes the transient phase. This attractor is not quiescent or unstructured: the steady-phase PCA and target decoding analyses (Fig. 2i, j) confirm that it maintains a target-separable, goal-directed representation throughout sustained control. Notably, steady-phase data points cluster more tightly in sustained trials than in ballistic trials (Fig. 4c, d, and Supplementary Fig. 8a–n), even though their flow field vectors have similar magnitudes. This mismatch suggests that condition-specific inputs have a significant influence on the slow-moving attractor in the C3D2 rSLDS.

Unlike the single-matrix LDS, the two-state rSLDS assigns a separate dynamics matrix to each discrete state, enabling a phase-specific quantification of rotational structure. We computed the skew-symmetric ratio and dominant angular velocity for each discrete state’s dynamics matrix (Fig. 4e, f). The relationship between transient-phase and steady-phase rotational metrics varied across sessions, participants, cortical subregions, and tasks: in some cases, the transient-phase discrete state exhibited a substantially higher skew-symmetric ratio than the steady-phase state, while in others the two were comparable or reversed. The fractional occupancy of each discrete state also varied across these factors (Fig. 4e, f, annotations). This variability suggests that rotational structure in the fitted dynamics is not a fixed, phase-locked property of MC but is modulated by task and recording context. Notably, the absence of a robust phase-dependent shift in rotational metrics contrasts with the consistent phase dependence observed in neural speed and cross-condition discrepancy (Fig. 2a, g), indicating that the transient-to-steady transition is better characterized by a change in the rate of state evolution than by a collapse of rotational dynamics.

The fraction of trials in each discrete state over time (Fig. 4g and Supplementary Fig. 9) shows a consistent pattern: most trials under both conditions begin in the transient phase, switch after similar durations, and then remain in the steady phase. Because sustained trials are longer, the steady phase dominates their time course. Overlaying discrete states on individual cursor traces (Fig. 4h, i) shows how the latent state maps onto each trial: the transient phase spans the high-acceleration segment of the reach, while the steady phase corresponds to low-acceleration hold and correction. The transient-state durations are similar in ballistic and sustained trials (participant JJ: mean Δ = − 0.012 s, 95% CI [−0.023, −0.000] s, *p* = 0.164, rank biserial *r* = 0.086; participant RD MCL: mean Δ = 0.018 s, 95% CI [0.007, 0.028] s, *p* < 0.001, rank biserial *r* = − 0.136; participant RD MCM: mean Δ = − 0.007 s, 95% CI [−0.016, 0.001] s, *p* = 0.259, rank biserial *r* = 0.044; Mann–Whitney *U* test), meaning movements under different gains undergo similar accelerations.

Could additional nonlinearity improve the model’s fit? To find out, we varied the number of discrete states while sweeping the continuous-state dimension. To evaluate the models’ performance, we used four metrics: position error decoding^54^, dynamical state update process (DSUP) ratio^28^, forecast inference, and dimensional entropy difference (see “Methods”)(Supplementary Fig. 10). The decoding and inference performance are largely influenced by the number of continuous states, rather than the number of discrete states (nonlinearity) (Supplementary Fig. 10a, c, e, g). Increasing the nonlinearity helps the model explain more dynamics in the data represented by the DSUP ratio (Supplementary Fig. 10b, f). However, the resulting trial-level distributions of discrete states fail a critical consistency test: with *D* ≥ 3, the discrete states are assigned sporadically and lack a stable time-course across trials as quantified by the dimensional entropy difference (Supplementary Fig. 10d, h). Thus, additional nonlinear segments do not carve out separate gain-specific attractors. Because rSLDS approximates but does not exhaust all nonlinear autonomous systems, this result does not categorically rule out nonlinear autonomy. However, the residual cross-condition discrepancies are consistent with condition-specific inputs that modulate traversal speed and curvature within the shared two-state flow.

### Interleaved gain-scaling and distance-scaling tasks produced similar dynamics

The main task was block-interleaved, allowing participants to anticipate the cursor’s gain for the next movement before trial onset. This anticipation could, in principle, lead to different preparatory strategies and neural dynamics between conditions. To rule out anticipatory strategy differences as a source of the observed cross-condition effects, we tested an interleaved version of the ballistic-sustained center-out task in which gain varied unpredictably from trial to trial, while leaving the gain parameters themselves unchanged. The participant acquired the targets consistently (ballistic trial acquisition time: 0.84 s ± SD 0.20 s; sustained trial acquisition time: 1.69 s ± SD 0.16 s; 95% CI of mean difference: [0.82, 0.87] s) (Supplementary Fig. 1d–f). As designed, the durations of ballistic and sustained trials were largely separable.

Furthermore, the main task assumes that the explanatory variable is the duration of movements, but it does not decouple duration from the cursor gain. To determine whether the result is correlated with duration or with gain, we experimented with a distance-scaling BMI task (see “Methods”) involving near and far reaches with a unified cursor gain (Fig. 1c). We also refer to this task as the radial-grid task. The two types of reaches correspond to two eight-target sets, each with a different distance from the center cue. The participant acquired the targets consistently (near trial acquisition time: 0.75 s ± SD 0.15 s; far trial acquisition time: 1.55 s ± SD 0.40 s; 95% CI of mean difference: [0.76, 0.86] s) (Supplementary Fig. 1g–i).

Similar to the non-interleaved gain-scaling task, the interleaved gain-scaling and distance-scaling tasks show multiphasic neural dynamics seen in cross-validated neural speed (Supplementary Fig. 2c–f) and crossnobis distance (Supplementary Fig. 2i–l) with an initial peak during movement onset and subsequent return to baseline. The peak in neural speed shares similar behaviors where peak onset time and peak time in ballistic or near trials slightly precede those in sustained or far trials (Supplementary Fig. 2m, n) while peak magnitude and duration differ by task condition and brain area (Supplementary Fig. 2o, p).

Mid-movement cross-condition discrepancy were observed in interleaved gain-scaling and distance-scaling tasks, both in single neuron (Fig. 2e and Supplementary Fig. 3d–g) and population analyses (Fig. 2f, g), as well as inference results (Supplementary Fig. 7c–f). Condition-specific dwell times in the steady phase were observed in time-colored LDS trajectories (Supplementary Fig. 6o–z) as well as discrete-state colored rSLDS trajectories and flow fields (Supplementary Fig. 8o–z).

The three task variants were designed so that the confounds accompanying movement duration differ across tasks. In the gain-scaling task, duration covaries with decoder gain at fixed target distance; in the distance-scaling task, duration covaries with target distance at fixed gain; and in the interleaved gain-scaling task, the participant cannot anticipate the upcoming gain condition. Despite these different task structures, the same qualitative pattern emerges: multiphasic dynamics with a shared initial transient of comparable timing and magnitude (Fig. 2a, b, and Supplementary Fig. 2), and cross-condition discrepancies arising predominantly after the transient peak at both the single-neuron (Fig. 2e and Supplementary Fig. 3) and population levels (Fig. 2f, g, and Supplementary Fig. 4). This shared pattern cannot be explained by gain sensitivity alone (absent in the distance-scaling task), target distance encoding alone (absent in the gain-scaling task), or anticipatory strategy differences (absent in the interleaved task), indicating that the common factor is the requirement for sustained control beyond the initial ballistic transient.

## Discussion

The motor cortex’s precise mechanisms have been debated for decades. Recent studies have highlighted how the motor cortex (MC) can be modeled as an autonomous pattern generator during arm movement^13–19^. Given the similarities between able-bodied movements and BMI control^31,42,43,59^, an open question was whether autonomous dynamics would also generalize to MC activity during BMI control.

We found that the two participants generated MC activity during BMI control beyond the constraints of the autonomous dynamical systems hypothesis (aDSH). During able-bodied-like ballistic movements, MC latent trajectories qualitatively resembled previously described dynamics (Fig. 3a, c, and Supplementary Fig. 6)^13^. However, formal quantification via skew-symmetric decomposition of the fitted dynamics matrices revealed variable rotational structure across sessions, participants, and tasks, with no consistent phase-dependent shift between the transient and steady epochs (Fig. 4e, f). The transient-to-steady transition is therefore better characterized by a change in the rate of state evolution than by a collapse of rotational dynamics. When necessary for sustained BMI movements, the participant could easily sustain MC activity (Fig. 3b, d, and Supplementary Fig. 6) rather than strictly following autonomous dynamics. Interestingly, the MC activity was shared between movements of different conditions, despite the aDSH hypothesis that different initial conditions would lead to different movements^14,19^.

Our results indicate that an autonomous dynamical system is too strict a model for the wide temporal scales of possible BMI movements. A similar interpretation was previously reported, although without data^60^.

These results should not be construed to mean that MC has absolutely no constraints on neural activity. A series of studies have examined how the covariance structure of MC during natural movement (also known as a “neural manifold”) constrains one’s ability to generate arbitrary activity^61–63^. Neural activity patterns within this natural covariance structure are much easier to generate than outside it. However, our results suggest that there are no constraints on the dynamical trajectories to reach the different points on this manifold.

Because BMI control decouples MC activity from the limb’s biomechanics and natural proprioceptive loop, it enables the question of whether the autonomous dynamics seen in ballistic reaches persist when the temporal demands change. In other words, could the dynamics observed during ballistic reaches simply reflect the behavioral task and its timescale, rather than an intrinsic property of the motor cortex? What may seem like intrinsic dynamics may appear more like behavioral dynamics at different timescales. As shown here, dynamics learned from one behavioral timescale may not apply to similar behaviors performed at shorter or longer timescales. This was also one of the original pitfalls of standard RM; RM found neurons tuned to as many variables as were tested because oftentimes variables are highly correlated. Similarly, without joint behavioral-neural dynamics modeling^64^ or without higher-dimensional tasks^65^, it can be difficult to disentangle intrinsic neural dynamics from behavioral dynamics.

A related challenge is that movement duration covaries with other task parameters in goal-directed motor paradigms, including the non-human primate reaching tasks that established the aDSH. To mitigate this, we designed three task variants in which the confounds accompanying duration differ. The shared pattern across all three tasks argues against gain or distance as the primary driver of the observed effects. Accordingly, the central claim of this study concerns what happens when movements must be sustained beyond the initial ballistic transient, regardless of which task parameter produces the longer duration, rather than treating duration as an independently manipulable neural variable.

Across all three tasks, neural trajectories under different conditions share similar states at movement onset and similar evolution throughout the visual delay and transient phase (Supplementary Fig. 2m–p). Because our tasks lack an instructed delay period, these early epochs constitute the window in which condition-specific planning would first manifest. The evidence against the aDSH therefore rests not on this early similarity alone but on the subsequent condition-dependent divergence that emerges only during the steady phase (Figs. 2f, g, and 3k–n). The distance-scaling task makes this point particularly clear: near and far reaches require different movement distances, yet latent trajectories originate from comparable regions of state space at movement onset (Fig. 3e–h and Supplementary Fig. 6), contradicting the aDSH prediction that distinct movements should arise from distinct initial states^14,19^. Rather than a uniform time-warping of a shared trajectory, near trials are completed during or shortly after the transient, while far trials continue into a prolonged steady phase, indicating that distance-dependent modulation emerges during ongoing movement rather than being set by the initial state.

The two participants showed qualitatively consistent effects, including multiphasic neural dynamics, population-level cross-condition discrepancies, and failure of autonomous models to account for condition-specific trajectories, but differed in specific patterns. In participant RD, single-neuron cross-condition discrepancies were biased toward the steady phase (Fig. 2e and Supplementary Fig. 3), whereas in participant JJ the discrepancies were more evenly distributed across epochs, and steady-phase target decodability was stronger (Fig. 2j). These differences may reflect the participants’ distinct motor imagery strategies (see “Methods”). A position-oriented strategy, as used by participant RD, may sustain richer condition-dependent modulation throughout the movement, because the neural representation must continuously track the evolving cursor-target relationship. Conversely, the directional strategy used by participant JJ may produce a more stable steady-phase representation, consistent with his stronger target decodability during that phase. Despite these differences, the core finding that autonomous dynamics alone cannot account for sustained BMI movements held for both participants.

Most previous demonstrations of neural prosthetics have used stationary algorithms like linear regression to decode movement velocity^33^. Our results both explain the success of stationary algorithms, like linear regression, as well as outline paths forward for better decoding. Due to the difficulty of controlling modern BMIs, BMI trajectory control often spans multiple seconds, in which sustained neural activity takes precedence over shorter dynamical transients. In this regime, real-time sensitivity^49,53^ and fast error correction^66^ are more important than perfect first-time decoding. As BMIs begin to better approximate able-bodied precision^67–69^, the sustained component of neural activity becomes shorter, and the dynamical transients take up larger proportions of the movement duration.

To further improve decoding, decoders may want to take temporal structure into account, for example, explicitly modeling the different phases of BMI movement as motor intent switches from a resting state to a moving state^70,71^. Our results suggest that decoders should be sensitive to a minimum of two temporal contexts during movement execution: movement onset (transient phase) and sustained intent (steady phase). An additional movement hold state^44,70^ or rest state^72^ may also be useful for certain applications.

Under a dynamical systems framework, the divergence in dynamics between sustained and ballistic movements indicates that the recorded population receives different inputs between the two movement conditions. An open question is where these inputs originate from. During able-bodied reaches, the motor cortex receives rich proprioceptive inputs from the primary somatosensory cortex (S1), which can exhibit similar rotational dynamics^27^, as well as transformed visual information^73^, potentially from the posterior parietal cortex (PPC).

Other recent studies have also challenged the autonomous dynamical systems hypothesis by showing that somatosensory input can drive much of the motor cortical activity observed during movement execution^27,48^. For BMI users moving a cursor with a paralyzed effector, as in our study, somatosensory feedback is severed. Are the observed inputs to the motor cortex during sustained activity the direct result of visual feedback or something else? A BMI perturbation study found that visuomotor feedback is initially isolated from the BMI in an output-orthogonal dimension^74^, suggesting that such inputs would occupy a different subspace until the movement goal was updated otherwise. Furthermore, visual feedback of cursor position only weakly affects MC activity in the absence of movement^56^. However, a more targeted study would be necessary to rule out visual feedback as the direct modulation of MC activity.

Feedback is a core part of flexible movement generation, but the aDSH largely disregards it. A complementary framework, optimal feedback control (OFC)^75–78^, computes movement controls on the fly based on the difference between the current and desired state, accounting for behaviors that cannot be predicted at preparation time^33,36,74,79–81^. Many OFC models posit MC as a feedback controller integrating somatosensory, cerebellar, and other inputs^82,83^.

OFC and the dynamical systems literature have often stood in isolation from each other, yet both frameworks provide utility in understanding motor control. Recent work has begun to unify them under the idea of dynamical feedback control^84^. Kalidindi and Crevecoeur developed a formal framework linking OFC models to low-dimensional neural trajectories in motor cortex, showing that feedback control of random networks can reproduce key features of MC dynamics across tasks^85^. Notably, their model suggests that the dimensionality of neural trajectories is largely determined by the dimensionality of inputs to the network, including task and effector parameters, through the dominance of low-rank input into a high-dimensional system. Testing this prediction across tasks with varying control dimensionality is an interesting direction for future work.

## Methods

### Study participant

The study participant JJ is a right-handed, tetraplegic man. Approximately three years before this study, he sustained a spinal cord injury at the cervical level C4–C5. He has residual movement in his upper arms, but he is unable to move or feel his hands.

The study participant RD is a right-handed, tetraplegic man. Approximately five years before this study, he sustained a spinal cord injury at the cervical level C3–C4. He shows weak residual movements (twitches) of the wrists and thumbs. He is able to feel tingling sensations when he touches something with his hands, but he can not pinpoint the location of the sensation.

Both participants are part of a BMI clinical study (ClinicalTrials.gov Identifier: NCT01958086). They consented to this study after understanding its nature, objectives, and potential risks, as well as to the publication of their de-identified data. All procedures were approved by the Institutional Review Boards of the California Institute of Technology, Casa Colina Hospital and Centers for Healthcare and the University of California, Los Angeles. All ethical regulations relevant to human research participants were followed.

### Multielectrode array implant location

Participant JJ was implanted with two 96-channel NeuroPort Utah electrode arrays (Blackrock Neurotech) (Fig. 1a) about 20 months after injury. One array was implanted near the hand knob area of the left motor cortex (MC). A second array was implanted in the superior parietal lobule (SPL) of the left posterior parietal cortex (PPC).

Participant RD was implanted with four 64-channel NeuroPort Utah electrode arrays (Fig. 1b) about 60 months after injury. Two arrays were implanted near the hand knob area of the left MC. The other two arrays were implanted in the SPL and supramarginal gyrus (SMG) of the left PPC.

More details regarding the methodology for functional localization and implantation can be found in ref. ^34^. All arrays were used for online BMI decoding, although our analyses here only describe data from the MC implants.

### Neural data preprocessing

Using the NeuroPort system (Blackrock Neurotech), neural signals were recorded from the electrode arrays, amplified, and analog bandpass-filtered (0.3 Hz to 7.5 kHz) before being digitized (30 kHz, 250 nV resolution). A digital high-pass filter (250 Hz) was then applied to each electrode.

Threshold crossings were detected at a threshold of −3.5 × RMS (root-mean-square of an electrode’s voltage time series). Threshold crossings were used as features for participant JJ’s in-session BMI control during the ballistic-sustained center-out task. For participant RD’s in-session BMI control during both the ballistic-sustained center-out task and the radial-grid tasks, we used neural network-extracted features per electrode^86^.

For offline analyses, we used k-medoids clustering on each electrode to spike-sort the threshold-crossing waveforms. The first *n* ∈ {2, 3, 4} principal components were used as input features to k-medoids, where *n* was selected for each electrode to account for 95% of waveform variance. The gap criteria^87^ were used to determine the number of waveform clusters for each electrode.

Trials were filtered based on cursor behaviors, examined using two metrics: path efficiency and distance-to-target slope. Path efficiency is defined as $\frac{{\sum }_{t=0}^{t=T-1}\parallel {c}_{t+1}-{c}_{t}{\parallel }_{2}}{\parallel {c}_{T}{\parallel }_{2}}$, where *c*_*t*_ is the position of the cursor at time *t*, and *T* is the first time point where the cursor overlaps with the target. Path efficiency measures the straightness of the cursor trajectory during a trial. We discarded trials with path efficiency >1.5. On the other hand, the distance-to-target slope reveals if the cursor is heading toward the target. We discarded trials with positive distance-to-target slopes that occur more than 0.05 screen units (where a screen unit of 1 denotes the length of the shorter side of the screen) away from the origin (starting position of the cursor in each trial). The proportion of trials discarded per session is reported in Supplementary Table 1.

We processed the spike-sorted neural data into nonoverlapping bins of 10 ms, then applied Gaussian smoothing with a standard deviation (std) of 100 ms and a border factor of 10 to account for edge effects.

When fitting dynamical models (LDS and rSLDS), we trimmed the data to remove visual delay periods at the beginning of each trial. We removed 139 ms from participant JJ’s trials, 290 ms from participant RD’s trials recorded with the lateral MC array, and 275 ms from participant RD’s trials recorded with the medial MC array. This is done before binning the spike-sorted neural data. We also removed time stamps after target acquisition.

To visualize more detailed information in crossnobis analyses, we processed the spike-sorted neural data into nonoverlapping bins of 1 ms, then applied Gaussian smoothing with an std of 30  ms.

### Recording sessions

Experiments were conducted in recording sessions at Casa Colina Hospital and Centers for Healthcare. All tasks were performed with the participants seated in their motorized wheelchairs with their hands resting on their laps or an adjacent armrest. The participants viewed text cues on a 27-inch LCD monitor that occupied approximately 40 degrees of visual angle.

Each session consisted of a series of 2–5 min, uninterrupted “runs” of the task. The participants rested for several minutes between runs as needed. Supplementary Table 1 summarizes all session data used.

### Motor imagery strategies

The two participants employed different motor imagery strategies for BMI control. Participant JJ imagined moving his right thumb to control a joystick in the direction of the target, producing commands that were more directional and binary in their onset and offset. Participant RD imagined pointing his right finger at the cursor while moving toward the target location, yielding a more graded, position-like control signal. Each participant arrived at their strategy through experience, as the approach they found most effective for controlling the cursor. These self-reported strategies were consistent across sessions and are relevant to interpreting cross-participant differences in neural dynamics (see “Discussion”).

### Center-out-and-back calibration task

The center-out-and-back task (e.g.,^28,88^) was used to calibrate the decoder for each session. First, a computer-controlled cursor moved out to targets and back to the center. The participants simultaneously attempted to move their thumb or index finger as though they were controlling the cursor via an analog game controller stick. We refer to this process as open-loop calibration. Using this calibration data, we trained a decoder to predict cursor velocity from neural activity.

After open-loop calibration, the participants performed the center-out-and-back task with a relatively low gain. Partial computer assistance was sometimes applied to the cursor trajectories. The purpose of this follow-up task was to collect more data to train the decoder, as recommended by ref. ^88^.

In some sessions, the participants repeated the task with a small number of repetitions so they could familiarize themselves with the decoder behavior^89^.

### Center-out brain-machine interface task with variable gain-scaling

The participants used cursor BMIs to complete the center-out task^88,90^ under two decoder gain parameters: high gain for ballistic reaches and low gain for sustained reaches. We refer to this task as the ballistic-sustained center-out task. The reach duration was tuned to complete in approximately 500 ms (ballistic) or 2 s (sustained) for participant JJ using a velocity gain parameter of the neural decoder^89^. Because participant RD is not able to consistently perform ballistic reaches with completion times as short as 500 ms, we instead tuned his ballistic reaches to complete in approximately 750 ms. The ballistic-movement condition is designed to resemble prior NHP reaching experiments. Since closed-loop decoders cannot match the speed and accuracy of NHP behavior, we add computer assistance (see below “Methods”) to allow ballistic movements without the need for online corrections.

### Interleaved center-out brain-machine interface task with variable gain-scaling

The interleaved center-out task is identical to the center-out BMI task with variable gain scaling, except that its trials are interleaved. Only participant RD attempted this task. The goal of this task is to control for the effect of anticipation since the participant can no longer anticipate the cursor gain of the upcoming trial. We occasionally change the number of trials under different conditions, which the participant did not realize.

### Near-far center-out brain-machine interface task with variable distance

We performed an additional center-out task variant to simulate sustained versus ballistic reaches. We refer to this variant as the radial-grid task. In this task, targets were positioned at two different distances from the center (Fig. 1c). The participant was instructed to move the cursor to the target as soon as the target appeared and then relax once the target was reached. Near targets resulted in shorter movement durations, whereas far targets resulted in longer movement durations. This is functionally similar to modulating the decoder gain directly^89^, as was done in the previous ballistic-sustained task. Only Participant RD attempted this task. This task was only tested with interleaved trials.

### Computer-assisted control: weighted average

Weighted-average computer assistance blends the decoded velocity with a vector directly toward the target. This type of computer assistance was previously described as “attraction assistance.”^91^ Assistance values ranged from 0 (full BMI control) to 0.5 (assisted BMI control), where a value of 1 corresponds to full computer control.

This type of computer-assisted control was used by participant RD during both the gain-scaling ballistic-sustained center-out task and the variable distance radial-grid task.

### Computer-assisted control: error rail

Error rail attenuates the components of the decoded velocity that are not directly toward the target. (We denote the direct cursor-to-target vector as $\vec{d}$). This is accomplished by decomposing the decoding cursor velocity $\vec{v}$ into the component parallel to the target direction ${\vec{v}}_{\parallel }$ and the component orthogonal to the target direction ${\vec{v}}_{\perp }$. With assistance value *K*, the assisted cursor velocity $\widehat{v}$ is

$$\begin{array}{rcl}{\widehat{v}}_{\perp } & = & (1-K){\vec{v}}_{\perp }\qquad\quad\quad\qquad \\ {\widehat{v}}_{\parallel } & = & \left\{\begin{array}{ll}{\vec{v}}_{\parallel } \hfill & \,{{\rm{if}}}\,{\vec{v}}_{\parallel }\cdot \vec{d} > 0\\ (1-K){\vec{v}}_{\parallel } & \,{{\rm{otherwise}}}\end{array}\right.\\ \widehat{v} & = & {\widehat{v}}_{\perp }+{\widehat{v}}_{\parallel }\ \ \quad\quad\qquad\qquad\end{array}$$

The assistance value *K* was specified for each run and ranged from 0 (full BMI control) to 0.3, where a value of 1 restricts the decoder to motion directly towards the target. Error rail is similar in some ways to ortho-impedance^33,36^ but also attenuates parallel-component velocities in the reverse direction.

This type of computer-assisted control was used by participant JJ.

### Neural dynamical filter

During participant JJ’s center-out sessions, we preprocessed the neural activity by binning spike counts at nonoverlapping 30-ms bins, z-scoring the firing rates for each channel, and reducing the dimensionality to the first 15 principal components. We decoded movement intent from the reduced-dimensionality population activity using the neural dynamical filter (NDF)^28^ with a 10-dimensional latent state. NDF learns a latent-state LDS of the neural population activity. For online decoding, NDF linearly predicts kinematics from the dynamics-filtered latent states. The NDF is described in detail in ref. ^28^.

### Linear decoder with deep-learning-based feature extraction

In later tasks, recordings yielded relatively few high-SNR waveforms, so we switched from threshold-crossing rates to broadband features. We used a temporal convolutional neural network (denoted “FENet") to extract features from 30kHz-sampled raw voltage time series^86^.

Before inputting the FENet features into the linear decoder, we preprocessed them in a series of steps. First, we z-scored input features. Next, to prevent unexpected channel noise from disproportionately degrading decoding, we bound the z-scored values between [−3, 6] (s). Because FENet generates multiple features (*K* = 8) per electrode, we used partial least squares regression to reduce this number to two informative features (*K* = 2) for each electrode. Next, we reduced feature dimensionality by using partial least squares to predict the input features, which were smoothed using an 800-ms minimum-jerk kernel; this approach is analogous to a linear autoencoder and helps denoise data that is expected to be autocorrelated. Finally, the firing rates were smoothed with an exponential filter (time-constant = 585 ms)

### Cross-validated neural speed and crossnobis distance

Kao et al. define neural population speed as ∥*r*_*k* + 1_ − *r*_*k*_∥_2_^28^. We use a similar definition here, but address one statistical drawback in the original formulation: noise biases Euclidean distances upward^92^. In other words, adding independent Gaussian noise to the firing rates increases ∥*r*_*k* + 1_ − *r*_*k*_∥_2_, even though the neural population speed doesn’t appear to change otherwise. To find an unbiased estimate of the neural speed, it is useful to cross-validate speed estimates across independent partitions of trial repetitions^92^:

8 $$\parallel {r} ^{\cdot }{\parallel }_{cv}^{2}={\left[\frac{{r}_{k+1}-{r}_{k}}{\Delta t}\right]}_{A}{\left(\frac{{\Sigma }_{A}+{\Sigma }_{B}}{2}\right)}^{-1}{\left[\frac{{r}_{k+1}-{r}_{k}}{\Delta t}\right]}_{B}^{T}$$

where *A* and *B* indicate independent partitions of the trials, *Σ* is the (regularized^93^ noise covariance matrix, *r*_*k*_ are the firing rate vectors for time index *k* stacked across the respective partition’s trials, and *Δt* is the time difference between consecutive time indices. The units of $\parallel {r}^{\cdot }{\parallel }_{cv}^{2}$ are (Hz/s)^2^. Although one can take the signed square root to obtain more meaningful units, here we prioritize the unbiased properties of the metric^94,95^.

The cross-validated neural population speed $\parallel {r}^{\cdot }{\parallel }_{cv}^{2}$ is a modified version of the cross-validated Mahalanobis distance (or crossnobis distance)^92^. It estimates the noise-normalized magnitude of the neural velocity that is consistent across trial repetitions. Here, we often abbreviate this metric as “neural speed”.

We also use the unmodified crossnobis distance without the concept of time to analyze the differences in tuning across phases of a trial:

9 $$\parallel \Delta r{\parallel }_{cv}^{2}={\left[{r}_{{k}_{1}}-{r}_{{k}_{2}}\right]}_{A}{\left(\frac{{\Sigma }_{A}+{\Sigma }_{B}}{2}\right)}^{-1}{\left[{r}_{{k}_{1}}-{r}_{{k}_{2}}\right]}_{B}^{T}$$

where ${r}_{{k}_{1}}$ and ${r}_{{k}_{2}}$ are firing rate vectors at two time indices that are not necessarily consecutive. The units of $\parallel \Delta r{\parallel }_{cv}^{2}$ are *Hz*^2^.

For analyses that require time-aligned traces across trials (neural speed, crossnobis distance), trials within each condition were truncated to the duration of the shortest trial in that condition. To prevent outlier-short trials from excessively limiting the analysis window, the shortest 10% of trials within each condition were excluded before truncation.

In visualizations of cross-validated neural speed (Fig. 2a and Supplementary Fig. 2a–f) and crossnobis distance (Fig. 2b and Supplementary Fig. 2g–l), we preprocessed spike-sorted data by applying Gaussian smoothing with an std of 30 ms and border factor of 10 (to account for edge effects).

We define the peak time as the time (after trial start) at which the initial peak of cross-validated neural speed occurs. We use the full width at half maximum to define the duration of this peak. We define the onset time of the initial peak as the last timestamp where the cross-validated neural speed exceeds 1.5 times the baseline standard deviation in each trial. The baseline is defined as the last 200 ms of the inter-trial interval (ITI).

### Visual delay period

We refer to the goal-directed motor responses to visual signals defined in ref. ^82^ as the visual delay period. We compute the visual delay period as the time interval between the trial start and the peak onset in cross-validated neural speed.

### Dynamical models

We modeled the neural population dynamics using recurrent switching linear dynamical systems (rSLDS)^58^, a probabilistic state-space model that approximates complex, nonlinear dynamics by composing simpler linear components. The model posits three layers of structure at each time step *t*: a discrete state *z*_*t*_ ∈ {1,…,*D*}, a continuous latent state **x**_*t*_, and observed neural activity **y**_*t*_.

Each discrete state indexes a distinct set of linear dynamics. Conditioned on the active discrete state *z*_*t*_, the continuous state evolves according to Gaussian dynamics:

10 $${{{\bf{x}}}}_{t+1}={{{\bf{A}}}}_{{z}_{t}}{{{\bf{x}}}}_{t}+{{{\bf{b}}}}_{{z}_{t}}+{{{\bf{v}}}}_{t},\,{{{\bf{v}}}}_{t} \sim {{\mathcal{N}}}({{\bf{0}}},{{{\bf{Q}}}}_{{z}_{t}})$$

where ${{{\bf{A}}}}_{{z}_{t}}$ is the dynamics matrix, ${{{\bf{b}}}}_{{z}_{t}}$ is a bias vector, and ${{{\bf{Q}}}}_{{z}_{t}}$ is the process noise covariance, all specific to the active discrete state. The term “Gaussian dynamics” refers to this conditionally linear Gaussian state evolution: within each discrete state, the continuous state update is a linear function of the previous state plus Gaussian noise. Observations are generated from the continuous state via a linear emission model:

11 $${{{\bf{y}}}}_{t}={{\bf{C}}}{{{\bf{x}}}}_{t}+{{\bf{d}}}+{{{\bf{w}}}}_{t},\,{{{\bf{w}}}}_{t} \sim {{\mathcal{N}}}({{\bf{0}}},{{\bf{S}}})$$

where **C** is the emission matrix, **d** is the emission bias, and **S** is the observation noise covariance. Because we used the single-subspace option in SSM, the emission parameters **C** and **d** are shared across all discrete states; only the dynamics parameters (${{{\bf{A}}}}_{{z}_{t}}$, ${{{\bf{b}}}}_{{z}_{t}}$, ${{{\bf{Q}}}}_{{z}_{t}}$) switch with *z*_*t*_.

The “recurrent” aspect distinguishes the rSLDS from a standard SLDS. In a standard SLDS, transitions between discrete states follow a fixed transition matrix *p*(*z*_*t* + 1_∣*z*_*t*_) that is independent of the continuous state. In the rSLDS, transition probabilities additionally depend on the current continuous state through a stick-breaking logistic regression:

12 $$p({z}_{t+1}| {z}_{t},{{{\bf{x}}}}_{t})={\pi }_{{{\rm{SB}}}}({{{\bf{R}}}}_{{z}_{t}}{{{\bf{x}}}}_{t}+{{{\bf{r}}}}_{{z}_{t}})$$

where ${{{\bf{R}}}}_{{z}_{t}}$ is a weight matrix and ${{{\bf{r}}}}_{{z}_{t}}$ is a bias vector that together linearly project the continuous state into logits, and *π*_SB_ is a stick-breaking transformation that converts these logits into a categorical distribution over discrete states. The stick-breaking process sequentially allocates probability mass: for each discrete state in order, a logistic function determines the fraction of remaining probability assigned to that state. This recurrent dependence allows the model to learn that discrete state switches should occur in particular regions of the continuous latent space—for example, transitioning from a transient movement-onset state to a steady-state regime when the continuous trajectory crosses a learned boundary.

When *D* = 1, the rSLDS reduces to a standard LDS with a single dynamics matrix. We used the SSM package^58,96^ implementation with recurrent transitions, Gaussian dynamics, single-subspace orthogonal Gaussian emissions, and autoregressive hidden Markov model (ARHMM) initialization.

We swept the number of continuous states (C ∈ {2, 3,…,20}) and discrete states (*D* ∈ {1, 2, 3, 4, 6, 8, 12, 16}) with five-fold cross-validation. To evaluate the test performance, we used four metrics (Supplementary Fig. 10): (1) absolute angle error of decoded position error^54^, (2) DSUP ratios^28^, (3) variance explained in forecast inference, and (4) dimensional entropy difference.

The position error is an intention estimation method. Since the participants move the cursor to one of eight targets in each trial, a ground truth direction exists for the movement from each cursor position. We can decode the direction at each time point and compare its absolute difference to the ground truth.

The dynamical state update process (DSUP) ratio^28^ measures the proportion of state updates explained by the dynamical model as:

13 $${r}_{t}=\frac{\parallel ({{{\bf{A}}}}_{{z}_{t-1}}-{{\bf{I}}}){\widehat{{{\bf{x}}}}}_{t-1}+{{{\bf{b}}}}_{{z}_{t-1}}\parallel }{\parallel ({{{\bf{A}}}}_{{z}_{t-1}}-{{\bf{I}}}){\widehat{{{\bf{x}}}}}_{t-1}+{{{\bf{b}}}}_{{z}_{t-1}}\parallel +\parallel {{{\bf{K}}}}_{t}({{{\bf{y}}}}_{t}-{{\bf{C}}}({{{\bf{A}}}}_{{z}_{t-1}}{\widehat{{{\bf{x}}}}}_{t-1}+{{{\bf{b}}}}_{{z}_{t-1}})-{{\bf{d}}})\parallel }$$

where $\parallel ({{{\bf{A}}}}_{{z}_{t-1}}-{{\bf{I}}}){\widehat{{{\bf{x}}}}}_{t-1}+{{{\bf{b}}}}_{{z}_{t-1}}\parallel$ is the contribution by the dynamical model and the other quantity in the denominator is called the innovations process which models what cannot be explained in the neural data. Also, **K**_*t*_ and ${\widehat{{{\bf{x}}}}}_{t}$ are the Kalman gain and Kalman filter estimates of the continuous state, respectively. ***Σ***_*t*_, **N**, and **R** are the covariance matrices of the continuous states, dynamics, and observations, respectively.

Forecast inference predicts the next observation ${\widehat{{{\bf{y}}}}}_{t+1}$ from the current continuous state **x**_*t*_ by applying the learned dynamics and emission parameters as:

14 $${\widehat{{{\bf{x}}}}}_{t+1}={{{\bf{A}}}}_{{z}_{t}}{{{\bf{x}}}}_{t}+{{{\bf{b}}}}_{{z}_{t}}$$

15 $${\widehat{{{\bf{y}}}}}_{t+1}={{\bf{C}}}{\widehat{{{\bf{x}}}}}_{t+1}+{{\bf{d}}}$$

We can then compute the R2 difference between the predicted observation and the data.

We devised the dimensional entropy difference metric to quantify whether the discrete states learned by rSLDS consistently switch from one to another across trials. The metric favors having a single dominant state at each time point (i.e., low time-wise entropy) and penalizes the case where a single state dominates an entire trial (i.e., low trial-wise entropy). In other words, the metric rewards the scenario where: (1) at each time point *t*, most trials use the same state (yielding low entropy across trials at that time), and (2) within each trial *i*, the trial visits multiple states (yielding a high entropy over time within that trial). Mathematically, this means minimizing the average time-wise entropy while maximizing the average trial-wise entropy:

16 $${H}_{i}=-\frac{1}{ln{n}_{z}}\mathop{\sum }\limits_{z=0}^{{n}_{z}}{p}_{z}(i)ln({p}_{z}(I))$$

17 $${H}_{t}=-\frac{1}{ln2}[{\mathop{\max }\limits_{z}} \, {p}_{z}(t)ln({\mathop{\max }\limits_{z}} \, {p}_{z}(t))+(1-{\mathop{\max }\limits_{z}} \, {p}_{z}(t))ln(1-{\mathop{\max }\limits_{z}} \, {p}_{z}(t))]$$

18 $$H=\frac{1}{{n}_{trials}}\mathop{\sum }\limits_{i}{H}_{i}-\frac{1}{{n}_{times}}\mathop{\sum }\limits_{t}{H}_{t}$$

where *p*_*z*_(*i*) is the fraction of time steps in trial *i* spent in state *z*, and *p*_*z*_(*t*) is the fraction of trials in which state *z* appears at time *t*. Because each entropy term (*H*_*i*_ and *H*_*t*_) is normalized to [0, 1], the dimensional entropy difference *H* falls in [−1, 1].

Overall, position error and forecast inference (Supplementary Fig. 10) favor larger numbers of continuous states while being insensitive to discrete states. For DSUP ratios, having more discrete states is beneficial, but the performance gain diminishes as the number of discrete states increases, with the gain from D1 (LDS) to D2 being the most dramatic. For dimensional entropy difference, having two discrete states is significantly more favorable. We chose C3D2 for our analyses as this combination is simplistic, unlikely to overfit, facilitates visualization, and performs reasonably well in the metrics. Compared to LDS (D1), rSLDS (D2) performs similarly on position error as well as forecast and better on DSUP ratios. Compared to rSLDS (*D* ≥ 3), rSLDS (D2) performs significantly better on dimensional entropy difference.

### Skew-symmetric ratio and dominant angular velocity

To quantify the rotational structure of the fitted dynamics, we decomposed each discrete-time dynamics matrix **A** (from LDS) or **A**_*z*_ (from rSLDS) into its continuous-time equivalent **M** = **A** − **I** and then into symmetric and skew-symmetric components:

19 $${{{\bf{M}}}}_{{{\rm{sym}}}}=\frac{1}{2}({{\bf{M}}}+{{{\bf{M}}}}^{T})$$

20 $${{{\bf{M}}}}_{{{\rm{skew}}}}=\frac{1}{2}({{\bf{M}}}-{{{\bf{M}}}}^{T})$$

The skew-symmetric ratio is defined as ∥**M**_skew_∥_*F*_/∥**M**_sym_∥_*F*_, where ∥⋅∥_*F*_ denotes the Frobenius norm. Values greater than 1 indicate dynamics dominated by rotational (oscillatory) structure, whereas values less than 1 indicate predominantly non-rotational (decaying or amplifying) dynamics^13^.

The dominant angular velocity is defined as ${\max }_{i}| \,{{\rm{Im}}}\,({\lambda }_{i})|$, where *λ*_*i*_ are the eigenvalues of **M**, converted from rad/bin to rad/s using the bin width (10 ms). This quantity reflects the fastest rotational frequency present in the latent state evolution.

Because all models were fit jointly across speed conditions, a single dynamics matrix (LDS) or set of dynamics matrices (rSLDS) governs both ballistic and sustained trials. For the LDS, these metrics therefore characterize the overall rotational content of MC dynamics across the entire movement, and results are combined across conditions. For the rSLDS, the metrics are computed separately for each discrete state’s dynamics matrix **A**_*z*_, enabling comparison of rotational structure between movement phases. Discrete states were ordered by their temporal center of mass across trials and classified as transient (early) or steady (late); fractional occupancy is reported alongside each group.

### Statistics and reproducibility

Nonparametric tests were used throughout: Mann-Whitney U tests for unpaired comparisons and Wilcoxon signed-rank tests for paired comparisons. Most tests were two-sided; one-sided (greater) tests were used when the hypothesis was directional, specifically when testing whether peak neural speed, crossnobis distance, or model performance metrics exceeded baseline or chance levels. When multiple comparisons were performed across time bins, *p*-values were corrected using the false discovery rate (FDR) method. Effect sizes are reported as rank-biserial *r* for Mann–Whitney *U* tests and Cohen’s *d* for Wilcoxon signed-rank tests. Exact *n* values, test statistics, effect sizes, confidence intervals, and *p*-values are reported in the main text and figure captions. No covariates were tested.

Sample sizes were determined by the number of trials and recording sessions that each participant completed during clinical BMI sessions. Sessions independent recording days. No statistical method was used to predetermine sample size. Two participants were studied across 15 total sessions (participant JJ: 3 sessions, 407 neurons; participant RD: 12 sessions, 1809 neurons). Session-level trial counts and the percentage of trials discarded by pre-specified quality criteria are reported in Supplementary Table 1; exclusion criteria are described in the Neural data preprocessing subsection. For time-aligned analyses, the shortest 10% of trials per condition were additionally excluded to preserve the analysis window (see Neural population analyses). Each session constitutes an independent recording with a distinct neural population; within each session, individual trials served as the unit of replication. Key findings, including multiphasic neural dynamics, mid-movement cross-condition discrepancies, and two-state switching dynamics, were replicated across both participants. In participant RD, these findings were additionally replicated across cortical subregions (lateral and medial MC) and across all three task variants. All dynamical models (LDS and rSLDS) were fitted using a random seed of 42 for reproducibility. The experiments were not randomized, as task conditions were defined by decoder gain or target distance parameters. Investigators were not blinded to condition allocation during experiments or outcome assessment, as the experimental design required real-time adjustment of task parameters.

### Reporting summary

Further information on research design is available in the Nature Portfolio Reporting Summary linked to this article.

## Supplementary information

Transparent Peer Review File

Supplementary Information

Description of Additional Supplementary Files

Supplementary Movie 1

Reporting Summary

## Data Availability

The data supporting the findings of this study, including source data underlying statistical figures, are openly available via Zenodo at https://doi.org/10.5281/zenodo.19445138^97^. De-identified raw data are available from the corresponding authors on reasonable request.

## Code availability

Custom analysis code used in this study is openly available on GitHub at https://github.com/ynffsy/DurationModulatedDynamics and archived on Zenodo at https://doi.org/10.5281/zenodo.19933473^98^.
