# Supplementary material for: Duration-modulated neural population dynamics in humans during BMI controls
Source: Commun Biol. 2026 Jun 26;9:1354. doi: 10.1038/s42003-026-10369-8 (PMC13669311; doi:10.1038/s42003-026-10369-8)
Supplement: Supplementary file 1 [file 42003_2026_10369_MOESM1_ESM.pdf]

nature portfolio

Peer Review File

Duration-modulated neural population dynamics in humans during BMI controls

Corresponding Author: Ms Fei Yin

This manuscript has been previously submitted at another journal. This document only contains information relating to versions considered at Communications Biology.

This file contains all reviewer reports in order by version, followed by all author rebuttals in order by version.

Version 0:

Reviewer comments:

Reviewer #1

(Remarks to the Author)
One influential recent idea about motor cortex (MC) is the autonomous dynamical systems hypothesis (aDSH), which posits that, once movement is initiated, population activity evolves largely according to intrinsic dynamics, with different movements primarily specified by distinct preparatory (initial) states within a shared flow field. This study challenges the sufficiency of this view in intracortical BCI cursor control, where natural proprioceptive feedback from the limb is largely absent and control relies heavily on visual feedback. Using a clever manipulation of movement time scale (ballistic vs. sustained; achieved primarily via decoder gain and complemented by a distance-scaling control task), the authors show that neural trajectories start from similar regions of state space at movement onset but diverge later during the movement, particularly in the sustained/steady phase. Importantly, these mid-movement discrepancies persist even when fitting switching dynamical models intended to approximate nonlinear autonomous dynamics, suggesting that additional condition-dependent inputs modulate MC activity beyond a fixed autonomous flow.

I find this study valuable in experimentally demonstrating that motor cortical activity cannot be fully captured by a purely autonomous dynamical model. The results strongly suggest that activity patterns during sustained control are influenced by condition-dependent neuronal inputs, although the specific sources of these inputs were not identified. This interpretation is consistent with recent findings emphasizing input-driven or feedback-based dynamics in motor cortex (e.g., Sauerbrei et al., 2020; Kalidindi et al., 2021; Stavisky et al., 2017).

The manuscript is clearly written and the data analyses are thorough, employing several advanced modeling approaches. Nevertheless, several conceptual and methodological issues require further clarification, as detailed below.

1. Radial-grid task and preparatory states.
In the radial-grid task, the required behaviors differed not only in movement duration but also in target distance (near vs. far). If movement distance is considered a distinct movement parameter, the aDSH framework would predict that distinct movements arise from distinct preparatory (initial) states within a shared flow field. One might therefore expect differences in neural trajectories during the preparatory period between near and far targets. However, the reported trajectories appear to originate from overlapping regions of latent state space at movement onset.

Could the authors clarify how this observation is reconciled with the predictions of aDSH? Does this imply that movement distance is not encoded at the level of preparatory state, that distance-related modulation emerges later during movement execution, or that the difference is primarily reflected in temporal scaling along a shared trajectory? A more explicit discussion of these possibilities would help situate the findings within the dynamical framework.

2. Implications for duration encoding and the initial-condition hypothesis.
Related to the first point, if movement distance does not appear to be reflected in the preparatory neural state, this raises a question regarding the interpretation of the ballistic versus sustained conditions. Specifically, if distance-related differences are not encoded at movement onset, is it possible that movement duration is likewise not represented in the initial state? If so, the later divergence of trajectories may not, in itself, constitute evidence against the initial-condition hypothesis, but

instead reflect modulation during movement execution. Clarification on this issue would strengthen the logical link between the radial-grid results and the broader claim regarding aDSH.

3. Interpretation of onset similarity under gain manipulation.
The sustained condition was implemented by reducing the gain of the neural decoder, thereby altering the mapping between neural activity and cursor velocity. Under this manipulation, the plant dynamics of the closed-loop BCI system were changed, but the participants may not have needed to fundamentally alter their internal control strategy at movement onset. If the neural control policy remained similar across gain conditions, it would be expected that trajectories at movement initiation would appear similar.

In this case, the similarity of trajectories at movement onset may not necessarily reflect a shared preparatory state in the sense assumed by aDSH, but rather a shared control policy operating under different plant dynamics. It would be helpful if the authors could clarify whether the onset similarity genuinely tests the initial-condition hypothesis, or instead reflects an unchanged neural controller across gain manipulations.

Reviewer #2

(Remarks to the Author)

The authors present evidence that motor cortex activity during BMI control in humans varies during movement execution across timing conditions, while the initial neural state was the same. These observations are partially inconsistent with the idea that motor cortex activity is generated by an autonomous dynamical system whose trajectories are determined solely by their initial states. After highlighting the limitations of the aDSH, the authors further tested whether switching systems with different state transition matrices or nonlinear fits improved the model. Their conclusion was somewhat rushed, and several arguments seemed relegated to the Supplementary Material. The main conclusion put forward is that the data most likely reflect neural trajectories influenced by external inputs.

I must disclose that I am not an expert in BMI and therefore cannot comment on the technical aspects of the experimental data. Nevertheless, the study is interesting, and the conclusions help advance our current understanding of neural trajectories in primary motor cortex as a system driven by inputs rather than as an autonomous pattern generator. The study is important and timely. I only have suggestions for improvement, although some may be major if feasible.

• It is stated that only data from MC were analyzed. Could data from other areas be informative? In particular, if data from parietal areas display similar characteristics, this would further support the idea that MC is not an autonomous pattern generator, especially if it is qualitatively similar to sensory associative areas. Although activity in PPC is likely strongly impacted by the loss of limb afferent feedback, visual information and internal priors may form an amodal estimate in these regions, making comparison with motor cortex activity potentially insightful.

• Specific comment: I had substantial difficulty following the figures. Font sizes are very small, information is sparse, and the diagrams are not very intuitive. This applies specifically to Fig. 4a–c and Fig. 5e.

• Methods, point 4.1.3: Could the authors add the proportion of trials discarded?

• Section on dynamical models: I was under the impression that this section did not provide sufficient information to understand what was actually done. What is meant by Gaussian dynamics? How are the discrete states defined? How do they relate to the state transition matrix? I believe that a more extended presentation of the mathematical tools employed here would help the interested but non-expert reader.

• Although still available as a preprint, the following reference is in press (Cell Reports) and presents developments that are highly relevant to this study (Kalidindi and Crevecoeur, doi: https://doi.org/10.1101/2025.05.15.654192). This paper develops a formal framework linking optimal feedback control models of behavior to low-dimensional aspects of neural trajectories in motor cortex. It also suggests that the dimensionality of neural trajectories is determined by the dimensionality of the task or plant parameters, through the dominance of low-rank input into a high-dimensional system. Could the authors test this prediction? If there are trials with one- versus two-dimensional cursor control, how does the dimensionality of neural trajectories vary?

Reviewer #3

(Remarks to the Author)
Summary
The manuscript probes whether human motor cortex (MC) activity during BMI cursor control adheres to the autonomous dynamical systems hypothesis (aDSH) across ballistic versus sustained movements that differ in their durations. The authors argue that while initial conditions are shared across durations, mid movement trajectories diverge, with a steady phase (low neural speed) in sustained trials that purportedly reflects input driven maintenance of intent—contradicting a strictly autonomous account. While the dataset is valuable and the modeling technically strong, several interpretational and

design issues weaken the strength of the central claim. My general assessment is that this is a valuable dataset with careful modeling that reveals a compelling transient to steady distinction in BMI control. However, to support the strongest theoretical claims, the manuscript should visualize late phase intent directly (not infer it), and temper the autonomy vs. input framing to reflect modeling limits and BMI specific constraints. With these revisions, the paper would make a clearer, more rigorous contribution. Recommendation: Major Revision.

Figure 1 (Task, Behavior, and Trial Structure)
1. Clarity of “peak onset” terminology (methods/labels).
The term “peak onset” is ambiguous; it seems to denote the start of motor activation / end of visual delay rather than the time of the peak itself. Please rename (e.g., “onset of transient peak”) and define once, consistently across panels and text.
2. Behavioral traces: request for velocity profiles.
To better connect neural phases to behavior, please include cursor velocity profiles for ballistic and sustained conditions (and near vs. far in radial grid), ideally overlaid per condition. This will help interpret the timing of transient vs. steady neural epochs relative to movement kinematics.
3. Potential design confound: duration vs. gain vs. kinematics.
A core limitation is that movement duration is not decoupled from decoder gain in the main center–out task (longer/shorter durations are achieved by gain manipulation). As a result, neural differences may reflect gain sensitivity rather than duration per se. The radial grid control keeps gain fixed but changes in target distance, adding new confounds. Thus, across tasks it remains unclear whether effects are driven by duration, or gain, and distance.

Figure 2 (Neural Speed, Crossnobis Distance, Single Neuron Discrepancies)
2A/B Neural speed & crossnobis distance—interpretation of “steady” phase.
The steady phase (low cross validated neural speed) is central to the input driven claim. However, explicit evidence of late phase “intent” is not shown. As currently presented, the reader must infer that low neural speed still carries goal directed information. Please add a steady phase only PCA (or dPCA) showing target separable structure in late windows (e.g., final 200–300 ms). This would visualize “intent manifolds” and rule out the alternative that MC is idling, drifting, or reflecting only nonspecific arousal/visual signals.
2A (right; acquisition aligned) second transient.
In the acquisition aligned panel, there appears to be a secondary abrupt change in neural speed near target contact. Please clarify its origin (e.g., movement termination, task state change, or assistance logic). If it reflects a post movement reset, labeling the phase transition would aid interpretation.
2C/2D Example units vs. 2E population counts.
Figures 2C–D illustrate unit wise differences across conditions, but Figure 2E indicates that typically <5% of neurons show significant discrepancies (and premovement/post peak differences are not striking). Please clarify. In this context we have two requests:
Quantify whether the subset of discrepant neurons materially drives the steady phase effects (e.g., re compute neural speed and crossnobis after removing those units).
Report session wise statistics and subject wise heterogeneity (Subject JJ appears to show weaker effects; please discuss). These checks would link single unit significance to the population level claim.

Figure 3 (LDS Latent Trajectories; Divergence Before/After Peak; % Dimensions with Discrepancy)
3E/3F Single latent dimensions: strength and generality.
The divergence after peak onset is not consistently significant across all targets/conditions, and no statistics appear for 3G. Moreover, Subject JJ often shows null/weak effects here and in 2E, raising heterogeneity concerns. Please:
Provide target wise and session wise stats for the divergence.
Explicitly contrast JJ vs. RD to contextualize variability (e.g., differences in implants, assistance levels, training history) in the discussion.
Rotational structure not formally tested.
Earlier work identified rotational dynamics at onset via skew symmetric fits/jPCA. Here, rotation is mentioned qualitatively for ballistic trials, but no formal rotational quantification is provided. Including jPCA or skew symmetric fits on onset vs. steady epochs—and reporting variance explained/angular velocity/eigen spectra—would reveal whether rotation collapses in the steady phase and strengthen the argument that the system transitions from autonomous like onset to input dominated sustain.

Figure 4 (Same vs Cross Condition Inference, LDS Performance)
4A–D Model mismatch and time dependence.
The same vs cross condition gap is a key piece of evidence for condition specific inputs, especially in the steady phase. However, Supplementary Fig. S4 shows instances where cross condition inference briefly outperforms same condition (negative differences) at low latent dimensionalities early in the movement (≤0.5 s; one panel 0.5–1 s). Please explain why this occurs (e.g., over regularization, insufficient state complexity early on, shared onset structure). A short analysis varying regularization/latent dimension with confidence intervals would clarify when and why cross condition generalization breaks.
Does LDS fit equally well across conditions?
Please report goodness of fit (variance explained / forecast R^2) by condition for LDS, with statistics. If LDS fits the ballistic transient but degrades in the sustained steady phase, that directly supports the two regime interpretation.

Figure 5 (rSLDS; Discrete States; Flow Fields)
Phase segmentation vs. autonomy.
rSLDS nicely reveals a transient to steady segmentation and reduced flow field magnitudes in the steady state. However,

the inference that residual discrepancies must be input driven is too strong given model choices. With limited discrete states and a fixed emission structure, rSLDS may underspecify smooth nonlinear attractors; failure of higher D models to find stable states does not disprove nonlinear autonomy. Please temper claims to “consistent with input driven modulation” rather than a categorical rejection of autonomous nonlinear explanations.
Link to “intent”: steady phase latent structure.
To substantiate that the steady phase carry’s goal directed control, add a steady phase PCA/dPCA or a simple “progress to target” neural axis (ridge readout of cursor error), and plot its trajectory in the last 200–300 ms. This would demonstrate explicit motor intent when rotational/transient signatures have subsided.

Minor Points & Presentation
The term “pause” seems misleading since the neural activity is assumed to be structured, not silent. The authors could consider consider “low velocity steady state.” Instead.
Some figure elements are dense; adding schematics and consistent color/label keys per phase/condition would help. (In Fig 1 the brain schematics appeared inverted; please check figure orientation.)

Version 1:

Reviewer comments:

Reviewer #1

(Remarks to the Author)
Thank you for the clear and helpful response. I now understand that the key point is the divergence following shared initial states, rather than the onset similarity itself. This addresses my concern, and I find the explanation convincing.

Reviewer #2

(Remarks to the Author)
The authors have addressed my comments and I have no further recommendation.

Reviewer #3

(Remarks to the Author)
The authors have made major revisions to their manuscript and have addressed the concerns I had earlier. I think this manuscript reads well from my perspective and I have no further comments to add.

As for Fig 1 Brain schematic orientation. The confusion for me was in the particular orientation and aspect ratio. Now that the authors have shown the location of their arrays, the figure looks perceptually easier to understand.

Open Access This Peer Review File is licensed under a Creative Commons Attribution 4.0 International License, which

permits use, sharing, adaptation, distribution and reproduction in any medium or format, as long as you give appropriate credit to the original author(s) and the source, provide a link to the Creative Commons license, and indicate if changes were made.
In cases where reviewers are anonymous, credit should be given to 'Anonymous Referee' and the source.
The images or other third party material in this Peer Review File are included in the article's Creative Commons license, unless indicated otherwise in a credit line to the material. If material is not included in the article's Creative Commons license and your intended use is not permitted by statutory regulation or exceeds the permitted use, you will need to obtain permission directly from the copyright holder.
To view a copy of this license, visit https://creativecommons.org/licenses/by/4.0/

Referee expertise:

Referee #1: BMI, motor cortex

Referee #2: neural trajectories, PPC

Referee #3: human motor cortex

Reviewers' comments:

Reviewer #1 (Remarks to the Author):

One influential recent idea about motor cortex (MC) is the autonomous dynamical systems hypothesis (aDSH), which posits that, once movement is initiated, population activity evolves largely according to intrinsic dynamics, with different movements primarily specified by distinct preparatory (initial) states within a shared flow field. This study challenges the sufficiency of this view in intracortical BCI cursor control, where natural proprioceptive feedback from the limb is largely absent and control relies heavily on visual feedback. Using a clever manipulation of movement time scale (ballistic vs. sustained; achieved primarily via decoder gain and complemented by a distance-scaling control task), the authors show that neural trajectories start from similar regions of state space at movement onset but diverge later during the movement, particularly in the sustained/steady phase. Importantly, these mid-movement discrepancies persist even when fitting switching dynamical models intended to approximate nonlinear autonomous dynamics, suggesting that additional condition-dependent inputs modulate MC activity beyond a fixed autonomous flow.

I find this study valuable in experimentally demonstrating that motor cortical activity cannot be fully captured by a purely autonomous dynamical model. The results strongly suggest that activity patterns during sustained control are influenced by condition-dependent neuronal inputs, although the specific sources of these inputs were not identified. This interpretation is consistent with recent findings emphasizing input-driven or feedback-based dynamics in motor cortex (e.g., Sauerbrei et al., 2020; Kalidindi et al., 2021; Stavisky et al., 2017).

The manuscript is clearly written and the data analyses are thorough, employing several advanced modeling approaches. Nevertheless, several conceptual and methodological issues require further clarification, as detailed below.

1. Radial-grid task and preparatory states.
In the radial-grid task, the required behaviors differed not only in movement duration but also in target distance (near vs. far). If movement distance is considered a distinct movement parameter, the aDSH framework would predict that distinct movements arise from distinct preparatory (initial) states within a shared flow field. One might therefore expect differences in neural trajectories during the preparatory period between near and far targets. However, the reported trajectories appear to originate from overlapping regions of latent state space at movement onset.

Could the authors clarify how this observation is reconciled with the predictions of aDSH? Does this imply that movement distance is not encoded at the level of preparatory state, that distance-

related modulation emerges later during movement execution, or that the difference is primarily reflected in temporal scaling along a shared trajectory? A more explicit discussion of these possibilities would help situate the findings within the dynamical framework.

> We thank the reviewer for this thoughtful question. We agree that the overlapping trajectories at movement onset in the radial-grid task deserve more explicit discussion, and we have revised the manuscript accordingly **(Discussion Section 3.1, lines 470-478)**.
>
> The reviewer correctly identifies three possible interpretations for the overlapping initial states: (1) distance is not encoded preparatorily, (2) distance-related modulation emerges later, or (3) the difference is primarily temporal scaling along a shared trajectory. Our data are most consistent with interpretation (2) and argue against interpretation (3).
>
> Regarding temporal scaling, near and far trajectories do not differ by a uniform time-warping of a shared trajectory shape (see Figure 3e-h and Figure S7). Rather, the transient peaks in cross-validated neural speed align temporally across conditions — both near and far trials exhibit a similarly timed initial transient of comparable magnitude. Near trials complete during or shortly after this transient, while far trials continue into a prolonged low-velocity steady phase. This pattern is qualitatively different from what temporal scaling would predict (i.e., a common trajectory shape traversed at different speeds). Instead, the conditions share a common transient and differ in the presence and duration of a subsequent steady phase —the same multiphasic structure observed in the ballistic vs. sustained center-out task.
>
> We note that our tasks do not include an instructed delay period: participants begin moving as soon as the target appears. This differs from the classic aDSH paradigm, where a delay period provides a clear preparatory epoch during which movement parameters are specified before a go cue. In our paradigm, "preparation" and "execution onset" are temporally overlapping, and the most natural operationalization of the initial condition is the neural state at movement onset (peak onset). The overlapping states at this time are consistent with distance not being fully specified preparatorily, with distance-dependent modulation emerging during ongoing movement.

2. Implications for duration encoding and the initial-condition hypothesis.
Related to the first point, if movement distance does not appear to be reflected in the preparatory neural state, this raises a question regarding the interpretation of the ballistic versus sustained conditions. Specifically, if distance-related differences are not encoded at movement onset, is it possible that movement duration is likewise not represented in the initial state? If so, the later divergence of trajectories may not, in itself, constitute evidence against the initial-condition hypothesis, but instead reflect modulation during movement execution. Clarification on this issue

would strengthen the logical link between the radial-grid results and the broader claim regarding aDSH.

> We appreciate the opportunity to clarify the logical structure of our argument. We do not claim that movement duration is encoded in the preparatory state. In fact, the shared initial conditions are a central part of our argument rather than a complication for it.
>
> Under a strict initial-condition hypothesis, trajectories initialized at the same state should evolve identically — the initial state fully determines the subsequent trajectory through autonomous dynamics (Equation 3). The observation that trajectories sharing a common initial state subsequently diverge in a condition-dependent manner, therefore, requires explanation. As discussed in our response to Point 1, this divergence is not temporal scaling: the transient peaks in cross-validated neural speed align temporally across conditions, with peak onset times largely comparable (Supplementary Figure 2m–p). Rather than traversing the same trajectory at different speeds, the system conditionally enters or bypasses an entire dynamical regime (the steady phase) depending on ongoing task demands (target distance in the radial-grid task, decoder gain in the center-out task).
>
> The reviewer raises the possibility that this divergence could reflect execution-phase modulation that is still compatible with aDSH. We acknowledge this possibility in principle, but note that accommodating it within the aDSH framework requires broadening the definition of "initial condition" to include information that only becomes available during movement execution — such as sensory feedback on the cursor's progress toward the target or the perceived change in decoder gain. At that point, the framework effectively incorporates ongoing external inputs, which is the central claim of our paper. The distinction between "a richer initial condition updated by sensory feedback" and "input-driven modulation during execution" becomes difficult to maintain.
>
> Moreover, the condition-dependent divergence persists even under a recurrent switching linear dynamical system (rSLDS), which approximates a broad family of nonlinear autonomous dynamics via piecewise-linear segments (Figure 4; Supplementary Figure 10). The rSLDS's inability to capture these discrepancies with additional discrete states suggests that the divergence is unlikely to be explained by unexplored nonlinearity alone.
>
> We have revised the discussion to make this reasoning more explicit and to clarify that the radial-grid results serve as converging evidence alongside the center-out results **(Discussion Section 3.1, lines 479-493)**. Both tasks independently show the same pattern of shared transients followed by condition-dependent steady-phase behavior rather than as a standalone refutation of the initial-condition hypothesis.

3. Interpretation of onset similarity under gain manipulation.

The sustained condition was implemented by reducing the gain of the neural decoder, thereby

altering the mapping between neural activity and cursor velocity. Under this manipulation, the plant dynamics of the closed-loop BCI system were changed, but the participants may not have needed to fundamentally alter their internal control strategy at movement onset. If the neural control policy remained similar across gain conditions, it would be expected that trajectories at movement initiation would appear similar.

In this case, the similarity of trajectories at movement onset may not necessarily reflect a shared preparatory state in the sense assumed by aDSH, but rather a shared control policy operating under different plant dynamics. It would be helpful if the authors could clarify whether the onset similarity genuinely tests the initial-condition hypothesis, or instead reflects an unchanged neural controller across gain manipulations.

> We agree that the onset similarity under gain manipulation does not, by itself, constitute a test of the initial-condition hypothesis. The participant may indeed employ a similar neural control policy at movement initiation regardless of decoder gain.
>
> However, our argument against the aDSH does not rest on the onset similarity alone. Rather, it rests on the combination of shared initial states followed by condition-dependent divergence in the subsequent trajectory. Under a strict initial-condition hypothesis, trajectories initialized at the same neural state should evolve identically through autonomous dynamics (Equation 3), regardless of the downstream decoder mapping. The observation that they diverge, specifically that sustained trials enter a prolonged low-velocity steady phase while ballistic trials terminate during or shortly after the transient, requires an explanation beyond autonomous dynamics. Whether the shared onset reflects a shared preparatory state (as aDSH would frame it) or a shared control policy (as the reviewer suggests) does not change this conclusion: something beyond the initial condition must account for the divergence. We have revised the discussion to clarify this reasoning **(Discussion Section 3.1, lines 479-493)**.

Reviewer #2 (Remarks to the Author):

The authors present evidence that motor cortex activity during BMI control in humans varies during movement execution across timing conditions, while the initial neural state was the same. These observations are partially inconsistent with the idea that motor cortex activity is generated by an autonomous dynamical system whose trajectories are determined solely by their initial states. After highlighting the limitations of the aDSH, the authors further tested whether switching systems with different state transition matrices or nonlinear fits improved the model. Their conclusion was somewhat rushed, and several arguments seemed relegated to the Supplementary Material. The main conclusion put forward is that the data most likely reflect neural trajectories influenced by external inputs.

I must disclose that I am not an expert in BMI and therefore cannot comment on the technical aspects of the experimental data. Nevertheless, the study is interesting, and the conclusions help advance our current understanding of neural trajectories in primary motor cortex as a system driven by inputs rather than as an autonomous pattern generator. The study is important and timely. I only have suggestions for improvement, although some may be major if feasible.

1. It is stated that only data from MC were analyzed. Could data from other areas be informative? In particular, if data from parietal areas display similar characteristics, this would further support the idea that MC is not an autonomous pattern generator, especially if it is qualitatively similar to sensory associative areas. Although activity in PPC is likely strongly impacted by the loss of limb afferent feedback, visual information and internal priors may form an amodal estimate in these regions, making comparison with motor cortex activity potentially insightful.

> We thank the reviewer for this insightful suggestion. Both participants were indeed implanted with arrays in the posterior parietal cortex (PPC) in addition to MC. However, the autonomous dynamical systems hypothesis (aDSH) is specifically a theory about motor cortex — it posits that MC acts as an autonomous pattern generator whose dynamics are determined by initial conditions. Our analyses were therefore designed to test this MC-specific claim, and including PPC would be tangential to the central question of the paper.

To avoid implying that PPC data were part of the present analyses, we have revised the manuscript to clarify that only MC recordings were analyzed. Specifically, we removed the mention of PPC from the opening Results sentence **(Results Section 2.1, lines 118-120)** and updated **Figure 1a,b** to show non-analyzed implant locations as dashed circles with an explanatory note in the caption. The Methods Section 4.1.2 continues to describe all implant locations for completeness.

Updated Figure 1a,b:

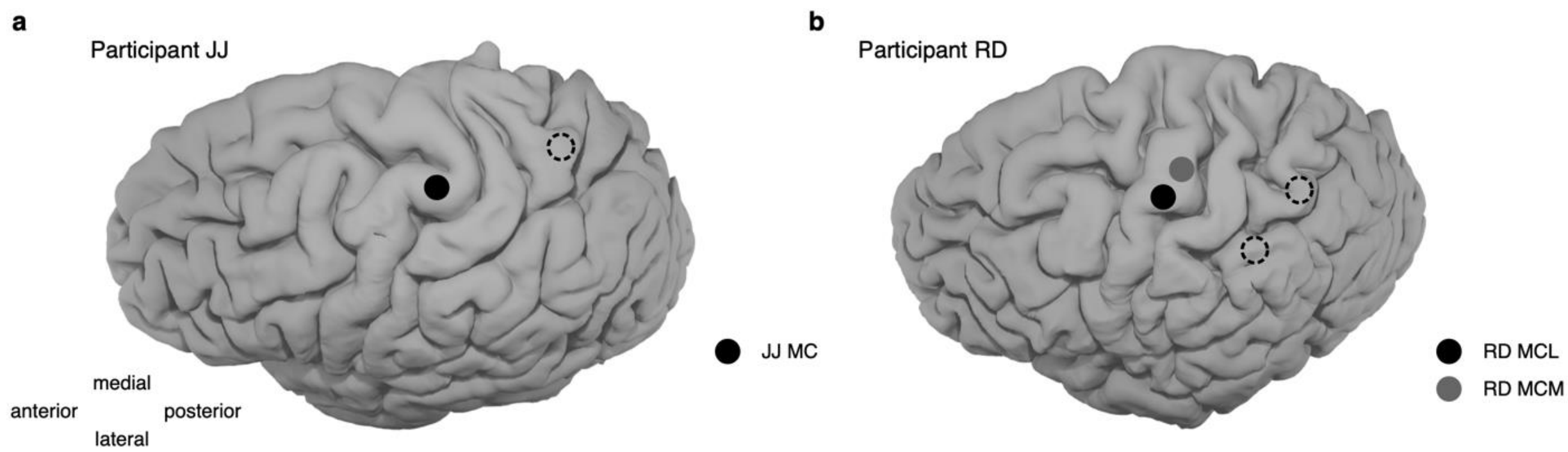

2. Specific comment: I had substantial difficulty following the figures. Font sizes are very small, information is sparse, and the diagrams are not very intuitive. This applies specifically to Fig. 4a–c and Fig. 5e.

We have made the following changes:

The previous Figure 4a-c (now **Figure 3k-m**) has been redesigned. The original waterfall plots and line plots have been replaced with heatmaps that display LDS inference performance as a function of both latent dimensionality and time. This format conveys the same information more compactly and makes the temporal pattern of same- versus cross-condition inference differences immediately visible. We have also added **Figure 3n**, which summarizes the transient-versus-steady comparison across all participants, brain areas, and tasks.

The previous Figure 5e (now Figure 4g) has been retained, as we were unable to identify specific sources of confusion. We have increased font sizes throughout the figures to meet the minimum 5 pt requirement specified by Communications Biology and improved readability where possible.

New Figure 3k-n:

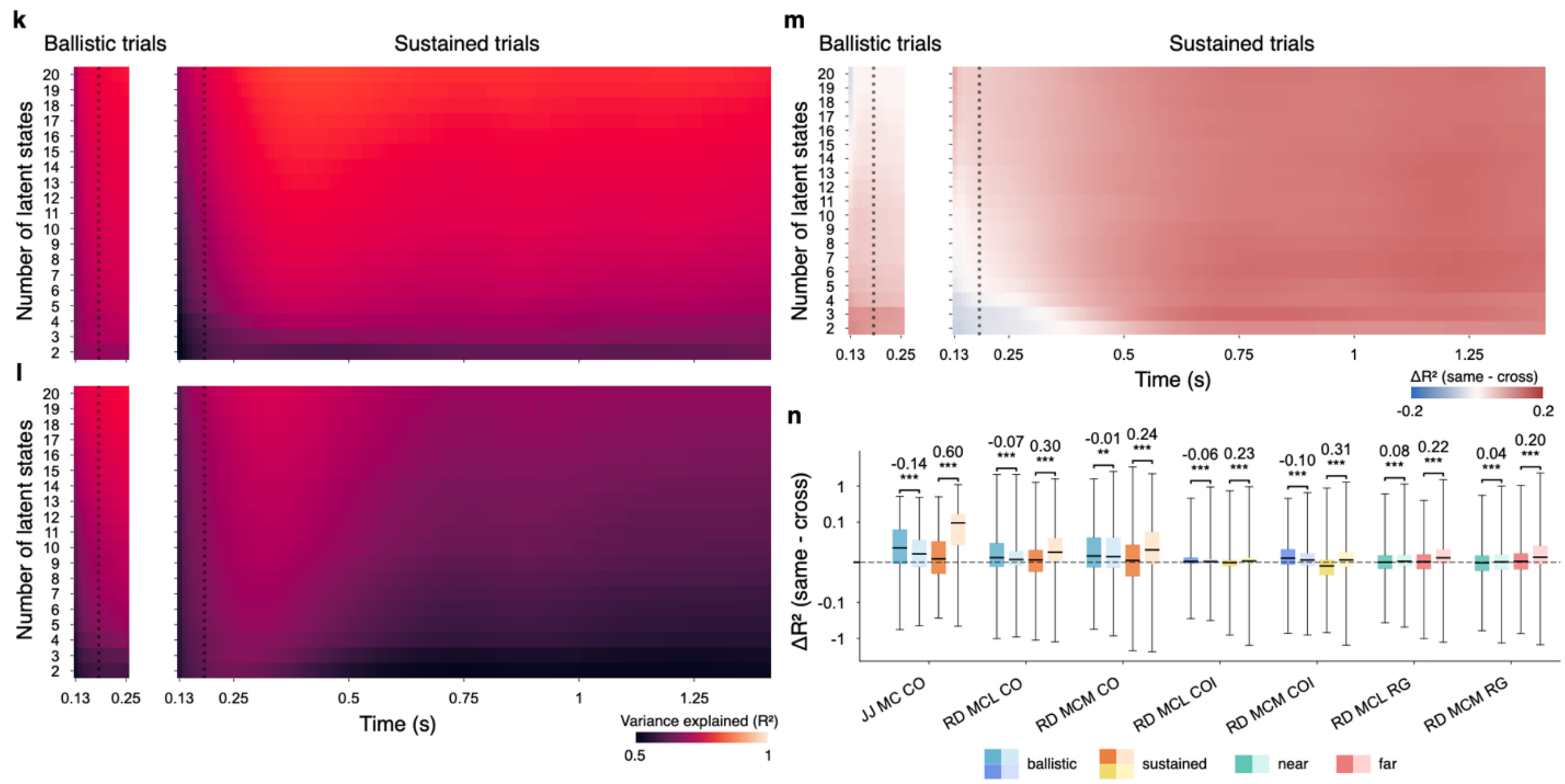

3. Methods, point 4.1.3: Could the authors add the proportion of trials discarded?

> We have updated **Table S1** to include the percentage of trials discarded for each session and condition and added a sentence in the **Methods (Section 4.1.3, lines 618-619)** directing readers to this information.

4. Section on dynamical models: I was under the impression that this section did not provide sufficient information to understand what was actually done. What is meant by Gaussian dynamics? How are the discrete states defined? How do they relate to the state transition matrix? I believe that a more extended presentation of the mathematical tools employed here would help the interested but non-expert reader.

> We have substantially expanded the "Dynamical models" section in the **Methods (Section 4.5, lines 783-815)** to provide a self-contained description of the rSLDS framework. The revised text now defines the three layers of the generative model (discrete states, continuous latent states, and observations) and their relationships, with the dynamics and emission equations presented explicitly (Eqs. 10, 11). We define "Gaussian dynamics" as the conditionally linear-Gaussian state evolution, where the continuous state update within each discrete state is a linear function of the previous state plus Gaussian noise. We explain how discrete states are defined as switching variables that each index a distinct set of linear dynamics parameters (dynamics matrix, bias, and process noise covariance), while the emission parameters are shared across all discrete states. Finally, we describe the recurrent state-transition mechanism, a stick-breaking logistic regression over the continuous state, that governs how

and where discrete state switches occur, thereby distinguishing the rSLDS from a standard SLDS with fixed transition probabilities.

5. Although still available as a preprint, the following reference is in press (Cell Reports) and presents developments that are highly relevant to this study (Kalidindi and Crevecoeur, doi: https://doi.org/10.1101/2025.05.15.654192). This paper develops a formal framework linking optimal feedback control models of behavior to low-dimensional aspects of neural trajectories in motor cortex. It also suggests that the dimensionality of neural trajectories is determined by the dimensionality of the task or plant parameters, through the dominance of low-rank input into a high-dimensional system. Could the authors test this prediction? If there are trials with one- versus two-dimensional cursor control, how does the dimensionality of neural trajectories vary?

We thank the reviewer for bringing this work to our attention. Kalidindi and Crevecoeur present a compelling framework linking optimal feedback control to low-dimensional neural dynamics, and their finding that the dimensionality of neural trajectories is shaped by the dimensionality of inputs to the network resonates with our emphasis on the role of external inputs to the motor cortex. We have added a discussion of their work in the "Unifying neural dynamics and flexible feedback" subsection **(Discussion Section 3.5, lines 557-564)**, noting their prediction about input dimensionality as an interesting direction for future work.

Our current dataset uses two-dimensional cursor control across all conditions and does not include a one-dimensional control comparison, so we are unable to directly test this specific prediction here, but we agree it is a valuable question for future experiments.

Reviewer #3 (Remarks to the Author):

Summary

The manuscript probes whether human motor cortex (MC) activity during BMI cursor control adheres to the autonomous dynamical systems hypothesis (aDSH) across ballistic versus sustained movements that differ in their durations. The authors argue that while initial conditions are shared across durations, mid movement trajectories diverge, with a steady phase (low neural speed) in sustained trials that purportedly reflects input driven maintenance of intent—contradicting a strictly autonomous account. While the dataset is valuable and the modeling technically strong, several interpretational and design issues weaken the strength of the central claim. My general assessment is that this is a valuable dataset with careful modeling that reveals a compelling transient to steady distinction in BMI control. However, to support the strongest theoretical claims, the manuscript should visualize late phase intent directly (not infer it), and temper the autonomy vs. input framing to reflect modeling limits and BMI specific constraints. With these revisions, the paper would make a clearer, more rigorous contribution. Recommendation: Major Revision.

Figure 1 (Task, Behavior, and Trial Structure)

1. Clarity of "peak onset" terminology (methods/labels).

The term "peak onset" is ambiguous; it seems to denote the start of motor activation / end of visual delay rather than the time of the peak itself. Please rename (e.g., "onset of transient peak") and define once, consistently across panels and text.

> We note that the term "peak onset" does not appear in Figure 1. It is first introduced in the context of Figure 2. The term most often appears as "peak onset time". It refers to a specific quantity defined in Methods — the time at which cross-validated neural speed first exceeds 1.5× the baseline standard deviation. We have added a brief clarifying note at its first use in **Figure 2's caption** to clarify this without requiring the reader to consult Methods. We prefer to retain "peak onset time" rather than "onset of transient peak" because the transient/steady phase distinction is derived from observing this peak — labeling it "transient" at this stage would be circular.

2. Behavioral traces: request for velocity profiles.

To better connect neural phases to behavior, please include cursor velocity profiles for ballistic and sustained conditions (and near vs. far in radial grid), ideally overlaid per condition. This will help interpret the timing of transient vs. steady neural epochs relative to movement kinematics.

We have added cursor speed profiles overlaid by condition for the center-out task **(Figure 1f)** and for all tasks and participants in **Supplementary Figure 1 (panels b, e, h)**. We have also expanded the text **(Results Section 2.2, lines 154-161)** to describe these speed profiles.

New Figure 1f:

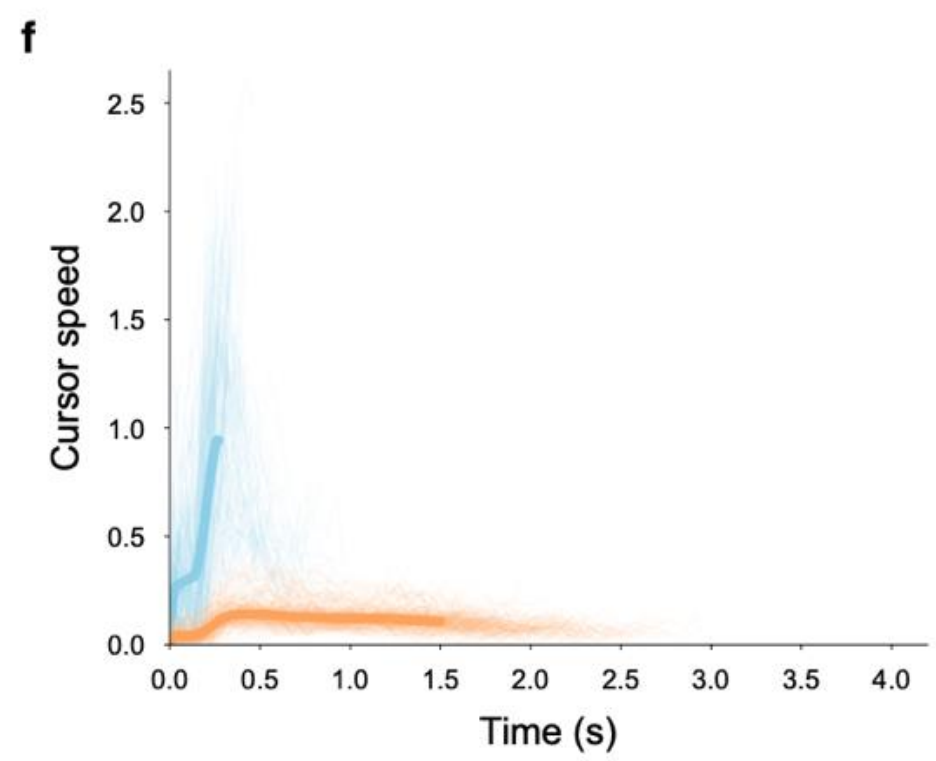

New Figure S1b,e,h:

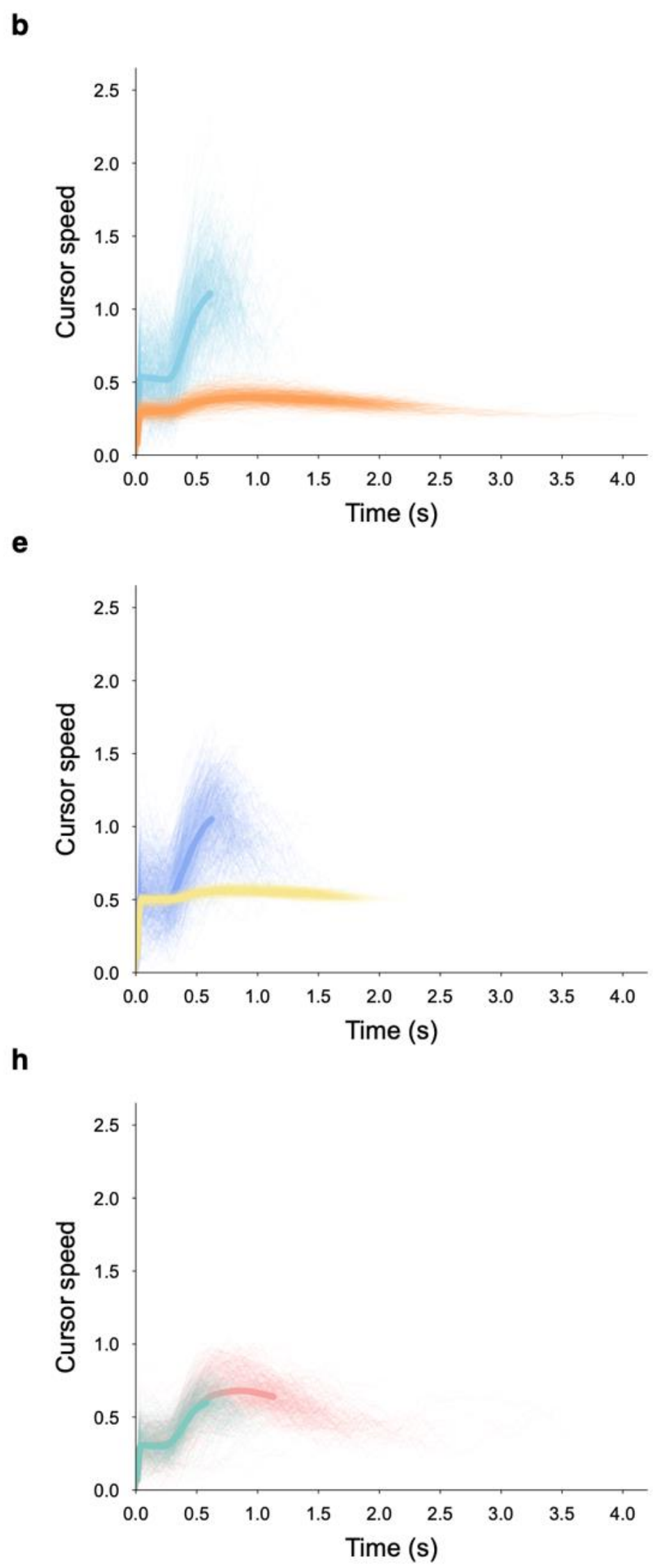

3. Potential design confound: duration vs. gain vs. kinematics.

A core limitation is that movement duration is not decoupled from decoder gain in the main center–out task (longer/shorter durations are achieved by gain manipulation). As a result, neural differences may reflect gain sensitivity rather than duration per se. The radial grid control keeps gain fixed but changes in target distance, adding new confounds. Thus, across tasks it remains unclear whether effects are driven by duration, or gain, and distance.

> We thank the reviewer for raising this point. Movement duration covaries with other task parameters in goal-directed motor paradigms, including the non-human primate reaching tasks that established the aDSH, and we designed our study with this consideration in mind. The three task variants each produce duration differences through a different mechanism: decoder gain at fixed target distance (gain-scaling task), target distance at fixed gain (distance-scaling task), and unpredictable gain condition (interleaved gain-scaling task). Despite these

different task structures, the same qualitative pattern emerges across all three: multiphasic dynamics with a shared initial transient of comparable timing and magnitude (Figure 2a,b; Figure S2), and cross-condition discrepancies arising predominantly after the transient peak at both the single-neuron (Figure 2e; Figure S3) and population levels (Figure 2f,g; Figure S4). This pattern is difficult to reconcile with gain sensitivity alone (absent in the distance-scaling task), target distance encoding alone (absent in the gain-scaling task), or anticipatory strategy differences (absent in the interleaved task). The common factor across tasks is the requirement for sustained goal-directed control beyond the initial ballistic transient, regardless of which task parameter produces the longer duration.

We have revised **Results Section 2.6 (lines 410-423)** to make this converging-evidence logic explicit and added a paragraph to the first Discussion subsection **(Discussion Section 3.1, lines 470-478)** clarifying that the central claim concerns what happens when movements must be sustained beyond the ballistic transient, rather than treating duration as an independently manipulable neural variable.

Figure 2 (Neural Speed, Crossnobis Distance, Single Neuron Discrepancies)

4. 2A/B Neural speed & crossnobis distance—interpretation of "steady" phase.

The steady phase (low cross validated neural speed) is central to the input driven claim. However, explicit evidence of late phase "intent" is not shown. As currently presented, the reader must infer that low neural speed still carries goal directed information. Please add a steady phase only PCA (or dPCA) showing target separable structure in late windows (e.g., final 200–300 ms). This would visualize "intent manifolds" and rule out the alternative that MC is idling, drifting, or reflecting only nonspecific arousal/visual signals.

We have added two new panels to Figure 2 that directly address this concern. **Figure 2i** shows PCA trajectories of MC population activity restricted to the final 300 ms before target acquisition in sustained trials. The top two principal components reveal clear target-separable clustering, demonstrating that steady-phase activity maintains structured, goal-directed representations rather than reflecting nonspecific arousal, drift, or neural idling. **Figure 2j** complements this visualization with a quantitative decoding analysis: a linear discriminant analysis (LDA) classifier trained on time-averaged firing rate vectors from the same 300 ms window decoded target direction well above chance level across both participants and tasks. Together, these analyses confirm that the low-neural-speed steady phase carries an active, target-specific representation of motor intent. Corresponding text has been added to **Results Section 2.3 (lines 202-211)**.

New Figure 2i,j:

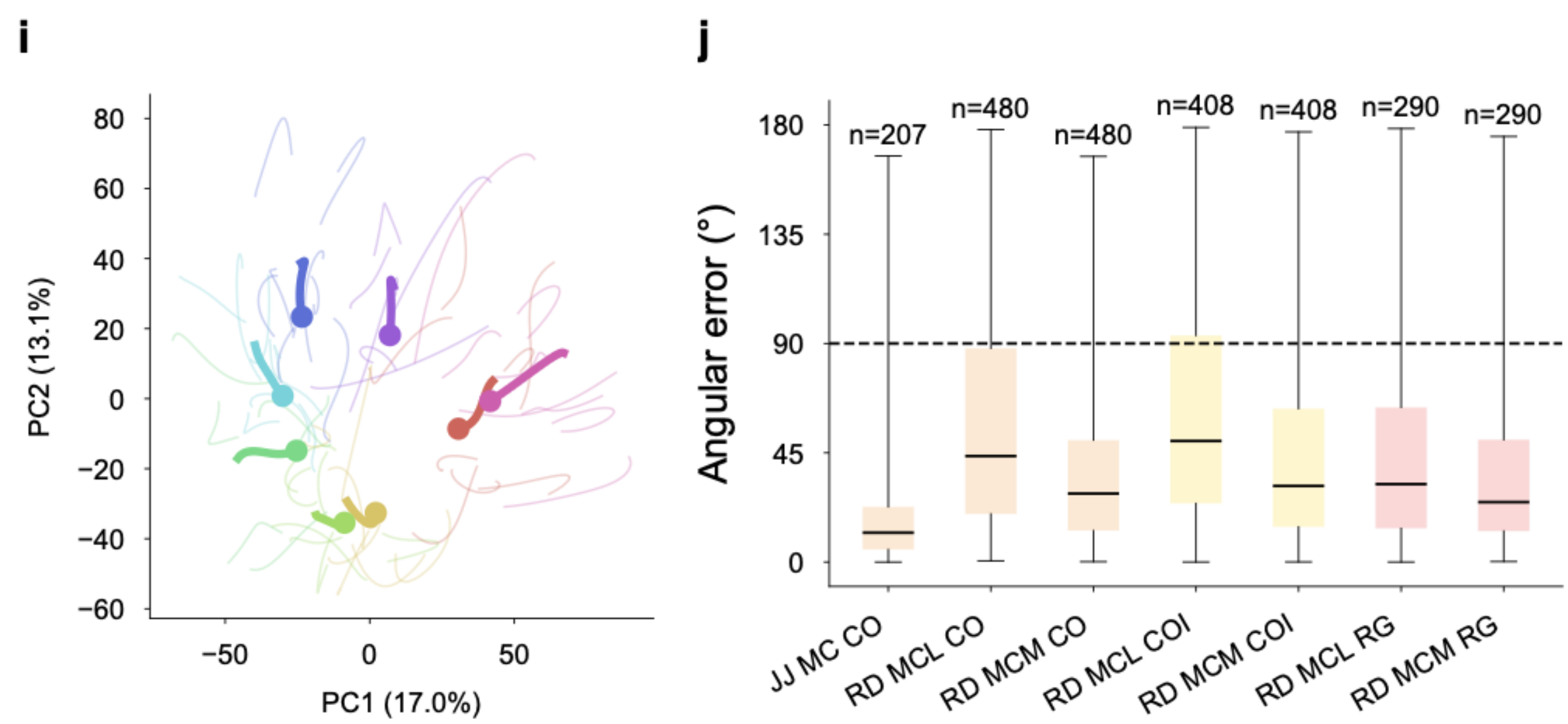

5. 2A (right; acquisition aligned) second transient.

In the acquisition aligned panel, there appears to be a secondary abrupt change in neural speed near target contact. Please clarify its origin (e.g., movement termination, task state change, or assistance logic). If it reflects a post movement reset, labeling the phase transition would aid interpretation.

> The secondary abrupt change in neural speed near target contact reflects movement offset — the transition from active BMI control to relaxation upon target acquisition. This feature is most prominent in Participant JJ, whose motor imagery strategy (imagined joystick deflections) produces more binary onset–offset commands, resulting in a sharper neural transient at movement termination. Participant RD employs a more graded, position-like imagery strategy, which yields a less abrupt offset (see **Methods Section 4.2.2, lines 643-651**, "Motor imagery strategies"; **Discussion Section 3.2, lines 495-509**, "Cross-participant heterogeneity").
>
> Regarding labeling: the acquisition-aligned panels in Figure 2a (right) and Supplementary Figure 2a–f (right halves) already align all trials to the target acquisition time (marked by the red dotted line), making the movement offset and its associated neural transient directly visible. We have added a sentence in the **Results (Section 2.3, lines 189-194)** to explicitly note that this secondary abrupt change reflects movement offset and to link it to the participants' differing motor imagery strategies.

6. 2C/2D Example units vs. 2E population counts.

Figures 2C–D illustrate unit wise differences across conditions, but Figure 2E indicates that typically <5% of neurons show significant discrepancies (and premovement/post peak differences are not striking). Please clarify. In this context we have two requests:

Quantify whether the subset of discrepant neurons materially drives the steady phase effects (e.g., re

compute neural speed and crossnobis after removing those units).
Report session wise statistics and subject wise heterogeneity (Subject JJ appears to show weaker effects; please discuss). These checks would link single unit significance to the population level claim.

We thank the reviewer for this important suggestion. We have revised the framing and added new analyses to clarify the relationship between single-neuron discrepancies and population-level effects.

First, we clarified the single-neuron results **(Results Section 2.4, lines 221-229)**. When tested per target direction, approximately 5% of neurons show significant cross-condition discrepancies during the steady phase (Figure 2e). However, the identity of these neurons varies across targets. When we instead ask whether a neuron shows a discrepancy for *any* target direction, the fraction rises to upward of 40% (Supplementary Figure 3, ALL columns). This indicates that mid-movement input-related modulation is broadly distributed across the recorded population rather than concentrated in a specialized subpopulation. Session-wise and target-wise breakdowns are shown in Supplementary Figure 3b–h, where each dot represents a single session's percentage of discrepant neurons for a given target direction and epoch.

To directly test whether these individually significant neurons drive the population-level effects, we performed the robustness check the reviewer suggested: we removed all neurons with significant firing rate differences between conditions (in any epoch or target direction) and recomputed within-condition neural speed **(Results Section 2.4, lines 235-242)**. For Participant JJ, the changes were not significant **(Figure 2h)**. For Participant RD, some epoch-level differences reached significance, but the overall pattern was preserved, with neural speed changing by at most 20% **(Supplementary Figure 5)**. This confirms that the population-level cross-condition discrepancy is not driven by the subset of individually significant neurons, consistent with a broadly distributed signal.

To provide a more direct population-level measure, we added cross-condition crossnobis distance computed at matched time points between ballistic and sustained trials **(Figure 2f,g; Supplementary Figure 4)**. Individual target traces are plotted in each panel, with data pooled across sessions for each participant–task–region combination. The cross-condition crossnobis distance is low during the transient phase and rises significantly above the pre-movement baseline during the steady phase (Mann–Whitney U test, FDR-corrected; Figure 2g). Significance was assessed by pooling across targets and sessions, which provides the most statistically meaningful test of the population-level effect.

Regarding cross-participant heterogeneity: the two participants showed qualitatively consistent effects but differed in specific patterns. In Participant RD, single-neuron discrepancies were biased toward the steady phase, whereas in Participant JJ they were more evenly distributed across epochs. Correspondingly, removing significant neurons had no effect for Participant JJ but produced some epoch-level changes for Participant RD. Meanwhile, steady-phase target decodability was stronger in Participant JJ (Figure 2j). We attribute these differences primarily to the participants' distinct motor imagery strategies: Participant JJ imagined directional joystick deflections (more binary onset–offset commands), whereas Participant RD imagined pointing toward the target (a more graded, position-like signal). We have added a description of these strategies to the **Methods (Section 4.2.2, lines 643-651)** and **Discussion (Section 3.2, lines 495-509)**.

New Figure 2h:

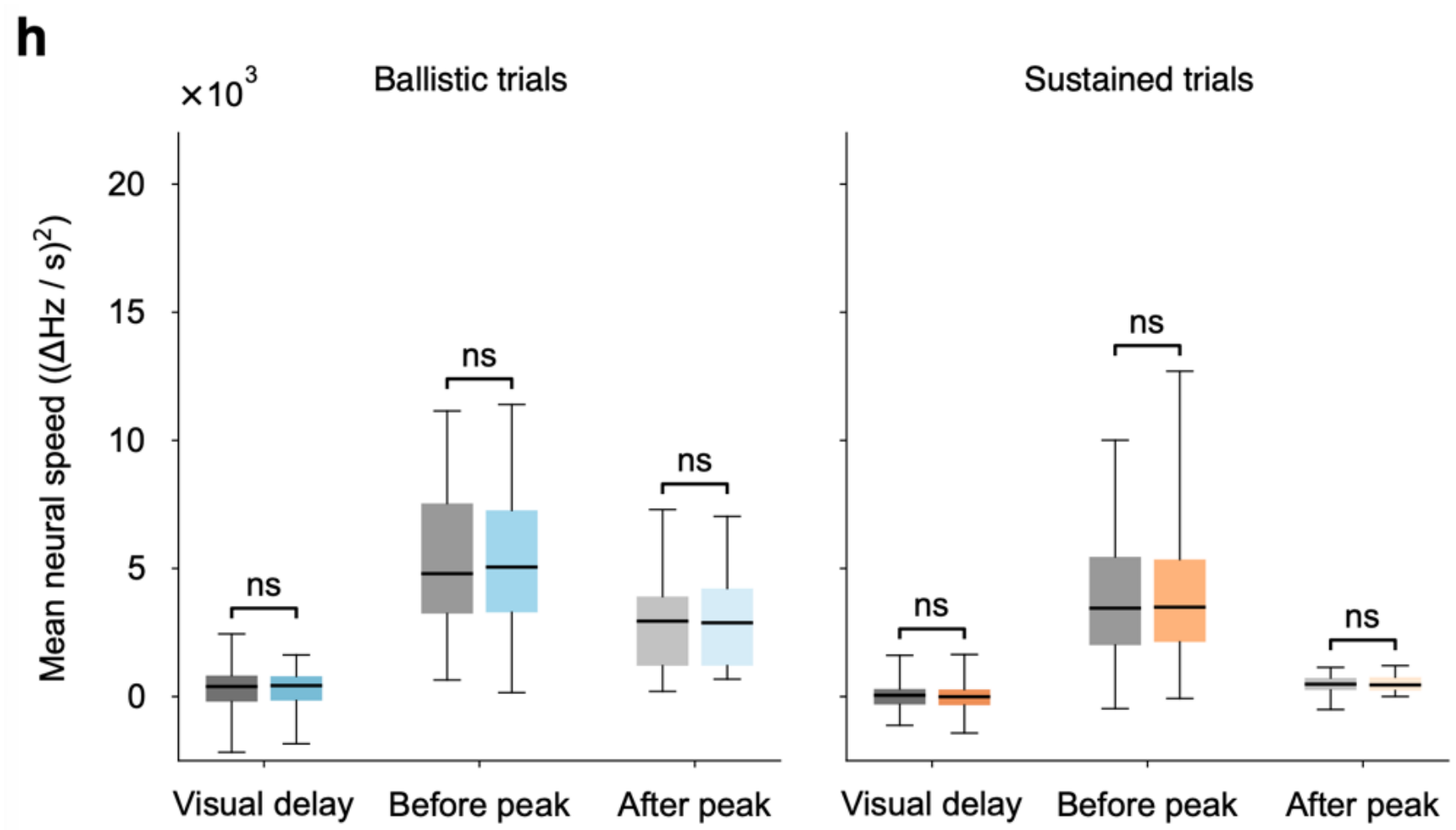

New Figure S5:

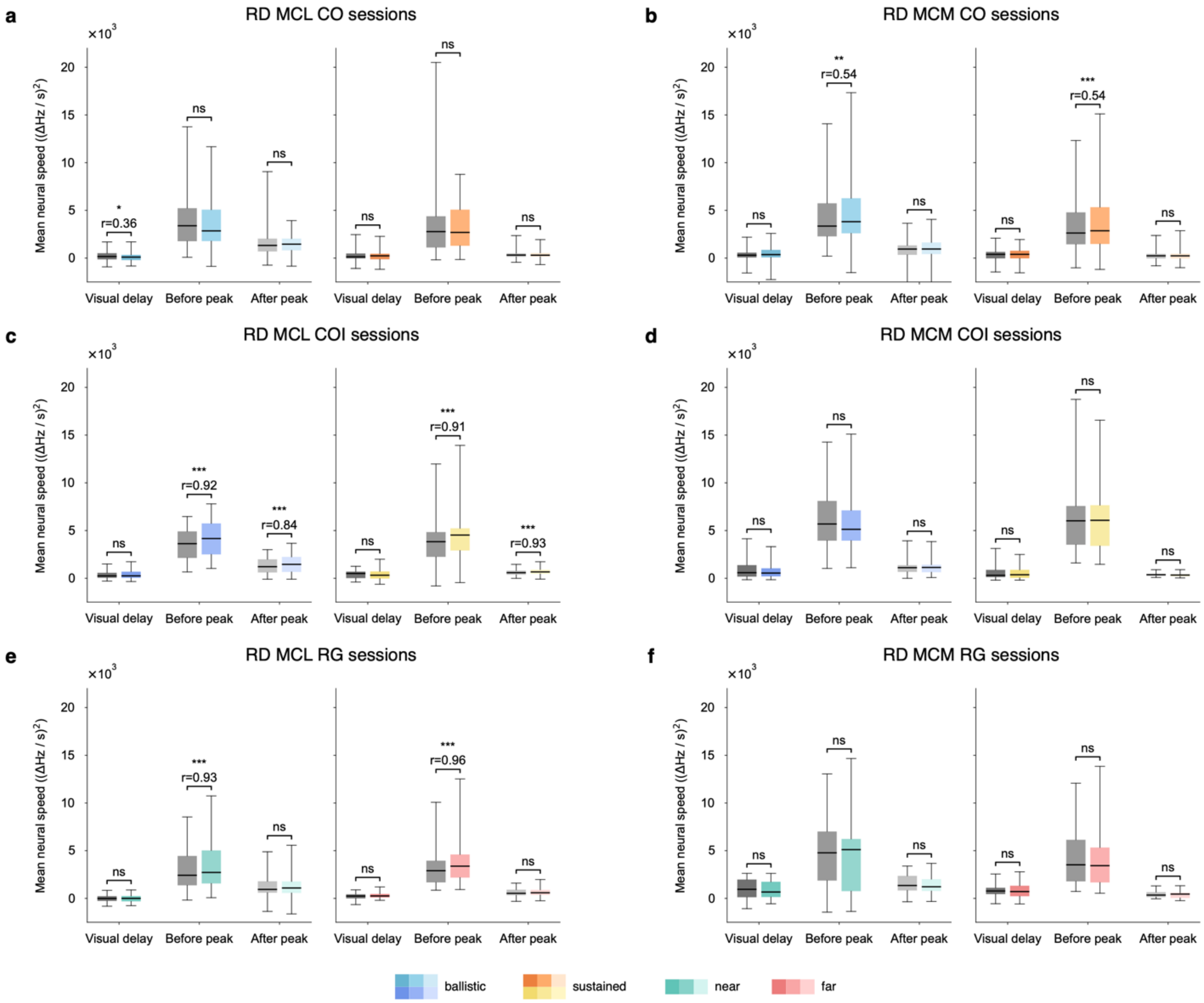

Figure 3 (LDS Latent Trajectories; Divergence Before/After Peak; % Dimensions with Discrepancy)

7. 3E/3F Single latent dimensions: strength and generality.

The divergence after peak onset is not consistently significant across all targets/conditions, and no statistics appear for 3G. Moreover, Subject JJ often shows null/weak effects here and in 2E, raising heterogeneity concerns. Please:

Provide target wise and session wise stats for the divergence.

Explicitly contrast JJ vs. RD to contextualize variability (e.g., differences in implants, assistance levels, training history) in the discussion.

> We agree with the reviewer that the original per-dimension significance tests (old Figures 3e–g) were difficult to interpret and inconsistently significant across targets. We have replaced this analysis with a population-level approach that directly addresses the reviewer's concerns.

The population-level cross-condition discrepancy is now quantified by the cross-condition crossnobis distance **(Figure 2f,g; Supplementary Figure 4)**, which measures the separation between ballistic and sustained neural states at matched time points. This provides a single, interpretable time-varying curve with per-target traces shown in each panel. Data are analyzed per participant, task, and brain region (each panel in Figure 2g and Supplementary Figure 4 corresponds to one participant–task–region combination, pooling across sessions). Significance was assessed by pooling across targets and sessions (Mann–Whitney U test, FDR-corrected), which is the most statistically meaningful way to evaluate the population-level effect given that individual target-by-session combinations have limited trial counts.

As additional converging evidence, the revised Figure 3 includes a cross-condition LDS inference test (panels K–N; Supplementary Figure 6): we train LDS models on one condition and evaluate inference on the other. Same-condition models consistently outperform cross-condition models, and this advantage is concentrated in the steady phase, particularly for sustained trials, where it persists even as model complexity increases. These results are shown per participant and task.

Regarding JJ vs. RD heterogeneity: as detailed in our response to the previous comment, the two participants show qualitatively consistent effects with differences in specific patterns attributable to their distinct motor imagery strategies (see **Methods Section 4.2.2, lines 643-651** and **Discussion Section 3.2, lines 495-509**). We note that despite differences in control strategy, both participants show the core finding that autonomous dynamics cannot fully account for sustained BMI movements.

New Figure 2f,g:

f

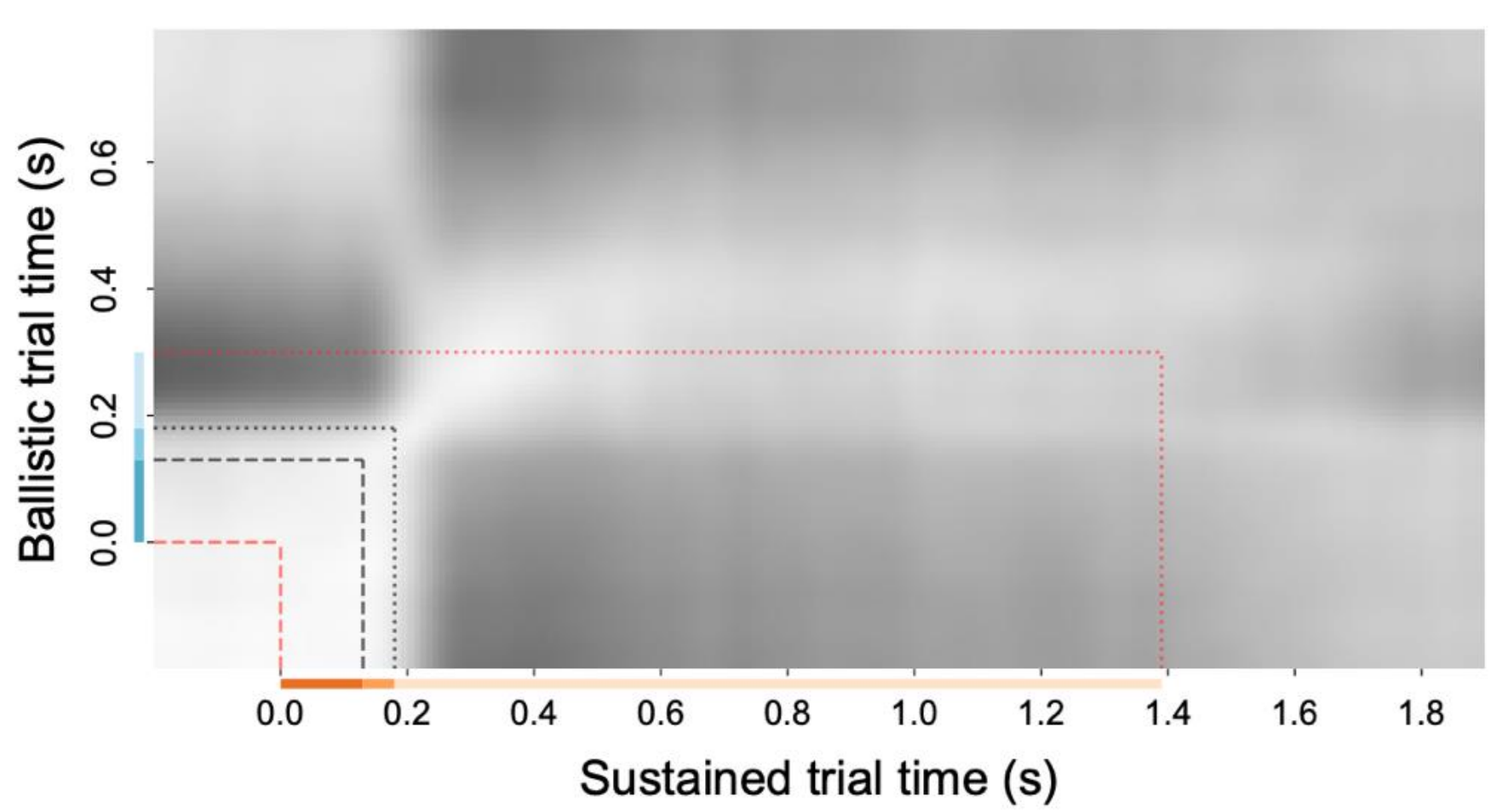

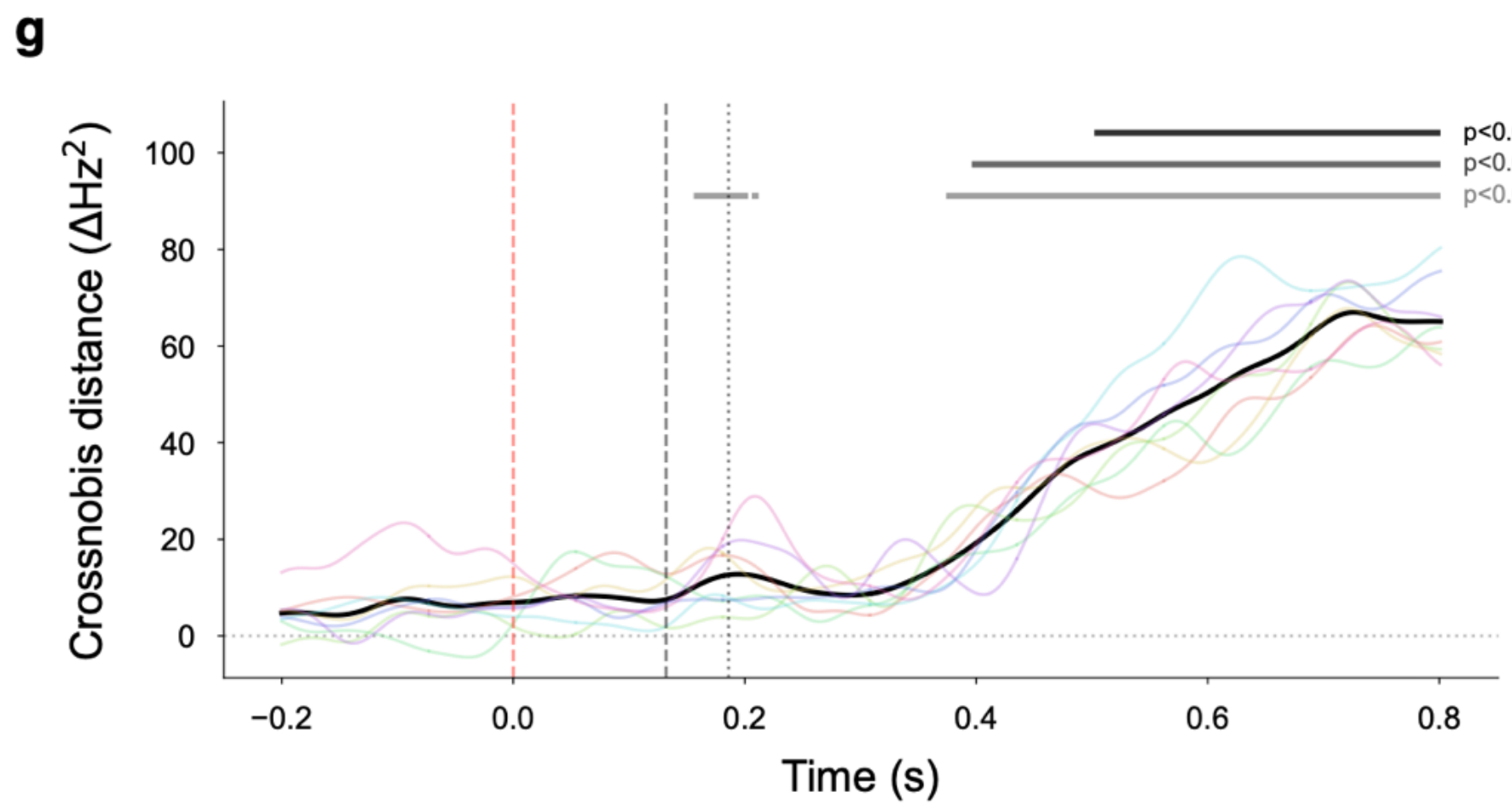

New Figure S4:

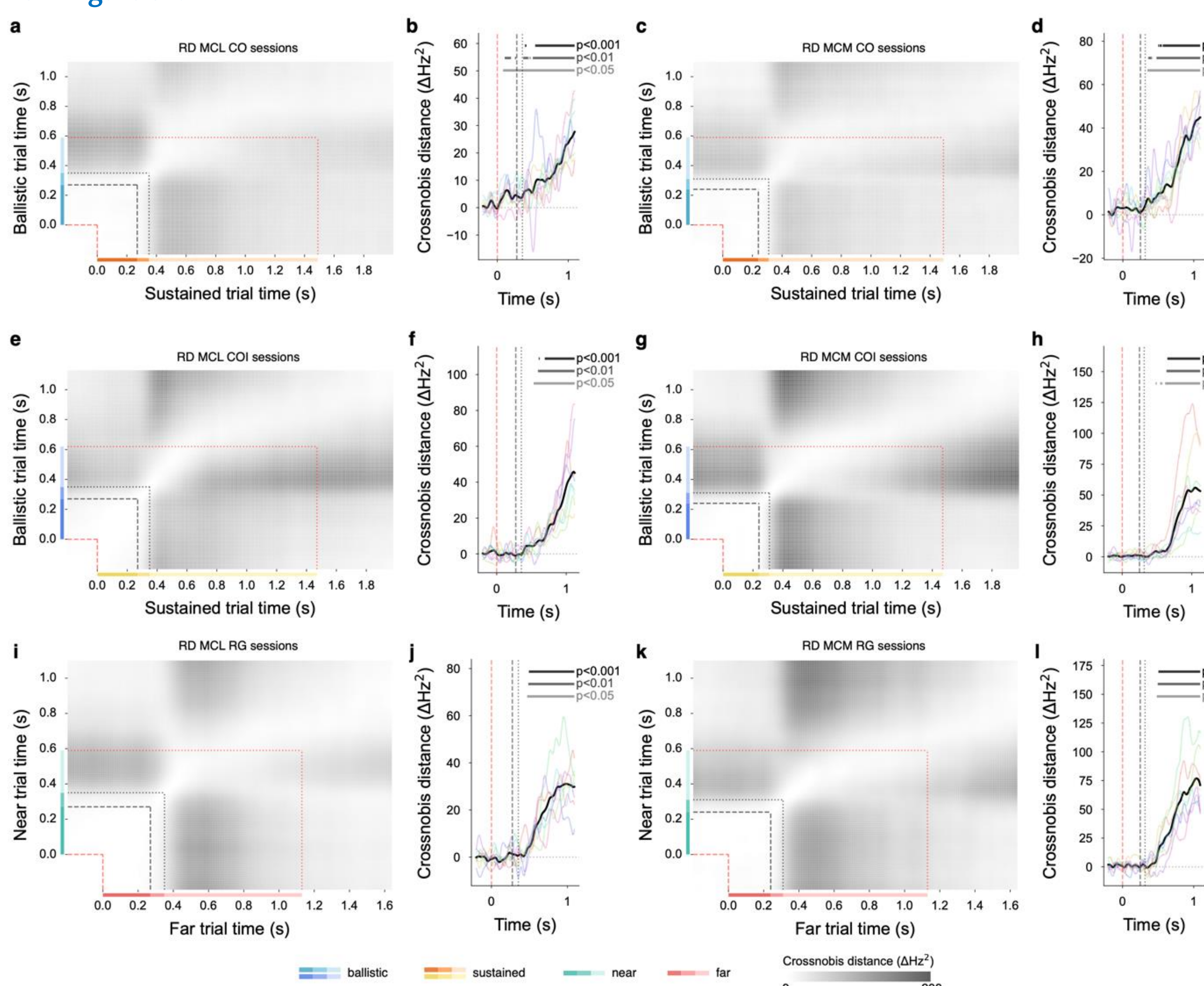

8. Rotational structure not formally tested.

Earlier work identified rotational dynamics at onset via skew symmetric fits/jPCA. Here, rotation is

mentioned qualitatively for ballistic trials, but no formal rotational quantification is provided. Including jPCA or skew symmetric fits on onset vs. steady epochs—and reporting variance explained/angular velocity/eigen spectra—would reveal whether rotation collapses in the steady phase and strengthen the argument that the system transitions from autonomous like onset to input dominated sustain.

We agree that formal quantification of rotational dynamics strengthens the manuscript and have now added this analysis.

Rather than applying jPCA, which has been shown to impose rotational structure on the data by constraining the optimization to skew-symmetric solutions (Lebedev et al. 2019), we quantified rotational content directly from the dynamics matrices of our already-fitted LDS and rSLDS models. Specifically, we decomposed the continuous-time dynamics matrix M = A – I into its symmetric and skew-symmetric components and computed the skew-symmetric ratio (‖M_skew‖_F / ‖M_sym‖_F), where values greater than 1 indicate rotationally dominated dynamics. We also computed the dominant angular velocity from the eigenvalues of M. These metrics are now reported for the LDS **(Figure 3i,j)** and separately for each discrete state of the rSLDS **(Figure 4e,f)**, with full methodological details in **Methods Section 4.5.6 (lines 866-885)**.

For the LDS, which fits a single time-invariant dynamics matrix jointly across conditions, the skew-symmetric ratio and angular velocity varied across sessions, participants, cortical subregions, and tasks (Figure 3i,j). For the rSLDS, the two-state model enables a phase-specific comparison: the transient-phase and steady-phase discrete states did not show a consistent difference in rotational metrics across sessions. In some cases, the transient state exhibited higher rotational content; in others, the relationship was comparable or reversed.

We interpret the variability in these rotational metrics as reflecting the complexity of the dynamical structure captured by our models. The latent trajectories encode not only rotational dynamics but also target-specific structure, producing flower-shaped manifolds (Figures 3a–d, 4a–d) rather than the simple circular orbits that rotational metrics are designed to summarize. A single skew-symmetric ratio computed from the full dynamics matrix, therefore, conflates rotational and non-rotational components of this richer structure. Despite this limitation, the analysis reveals that the transient-to-steady transition, the central finding of this study, is more consistently characterized by a change in the rate of neural state evolution (Figures 2a, 4c,d) than by a systematic shift in rotational content. This is consistent with our broader argument that the steady phase reflects input-modulated dynamics rather than a purely autonomous regime.

The additions appear in **Results (Section 2.4, lines 280-289; Section 2.5, lines 331-345)**, **Methods Section 4.5.6 (lines 866-885)**, and **Discussion (Section 3.1, line 436-441)**.

New Figure 3i,j:

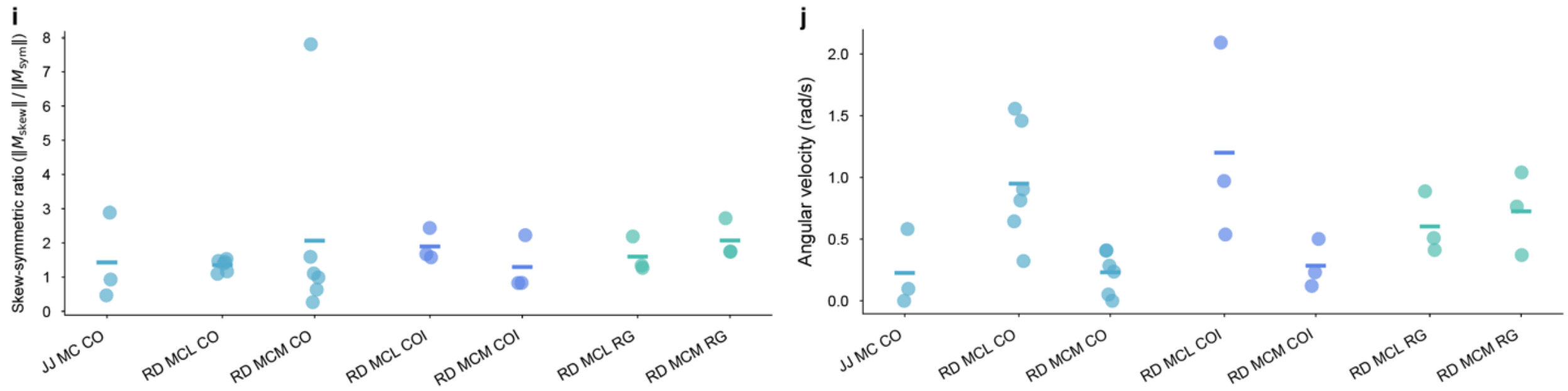

New Figure 4e,f:

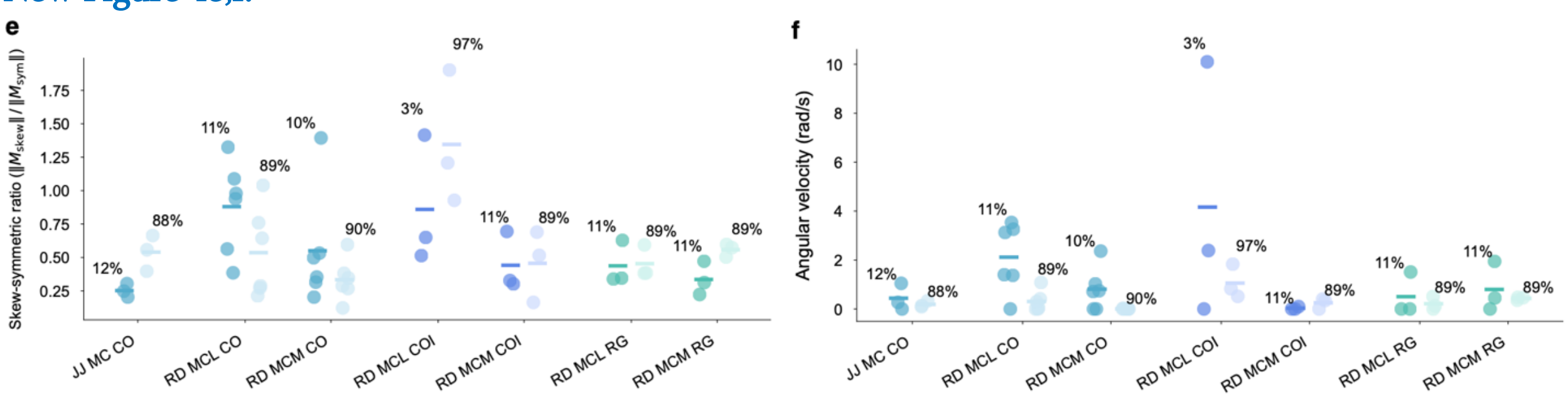

Figure 4 (Same vs Cross Cross-Condition Inference, LDS Performance)

9. 4A–D Model mismatch and time dependence.

The same vs cross condition gap is a key piece of evidence for condition specific inputs, especially in the steady phase. However, Supplementary Fig. S4 shows instances where cross condition inference briefly outperforms same condition (negative differences) at low latent dimensionalities early in the movement (≤0.5 s; one panel 0.5–1 s). Please explain why this occurs (e.g., over regularization, insufficient state complexity early on, shared onset structure). A short analysis varying regularization/latent dimension with confidence intervals would clarify when and why cross condition generalization breaks.

Does LDS fit equally well across conditions?

Please report goodness of fit (variance explained / forecast R^2) by condition for LDS, with statistics. If LDS fits the ballistic transient but degrades in the sustained steady phase, that directly supports the two regime interpretation.

We thank the reviewer for this observation. We have revised the main text **(Results Section 2.4, lines 262-279)** to address both points.

Cross-condition inference and the negative ΔR^2 values. The instances where cross-condition inference briefly outperforms same-condition inference (negative ΔR^2 in **Figure 3m; Supplementary Figure S6**) occur at low latent dimensionalities during the transient phase. This reflects the asymmetric temporal composition of the training data. Ballistic trials are

shorter and therefore contribute proportionally more transient-phase data to their training set. The resulting models capture transient dynamics well and can generalize to the early portion of sustained trials, where the dynamics are similar across conditions. Conversely, sustained-trained models spread their capacity across a much longer steady phase. At low latent dimensionalities, this imbalance allows a cross-condition (ballistic-trained) model to briefly outperform a same-condition (sustained-trained) model during the transient phase of sustained trials. As the number of latent dimensions increases, both models gain sufficient capacity to represent the transient, and the effect disappears, while the same-condition advantage in the steady phase persists.

LDS goodness of fit by condition. We have added **Figure 3n**, which partitions ΔR^2 (same-condition minus cross-condition inference R^2) into transient and steady phases for every participant, brain area, and task combination. For sustained trials, the steady-phase ΔR^2 is consistently and significantly larger than the transient-phase ΔR^2 (two-sided Mann-Whitney U tests with rank-biserial effect sizes reported in the figure). For ballistic trials, the transient-to-steady difference is smaller and less consistent. We note that this partition uses peak time as the phase boundary, and multiple factors could contribute to the observed asymmetry: the limited amount of steady-phase training data in the brief ballistic trials, genuine condition-specific dynamics in the steady phase, or the possibility that ballistic trials terminate before fully transitioning to a distinct steady-state regime. The LDS partition alone cannot disambiguate these factors. However, the pattern is consistent with the broader, partition-independent evidence that condition-specific structure emerges predominantly after the initial transient, as shown by cross-condition crossnobis divergence over continuous time (Figure 2f,g), single-neuron discrepancies (Figure 2e), and rSLDS discrete-state assignments (Figure 4g).

New Figure 3m:

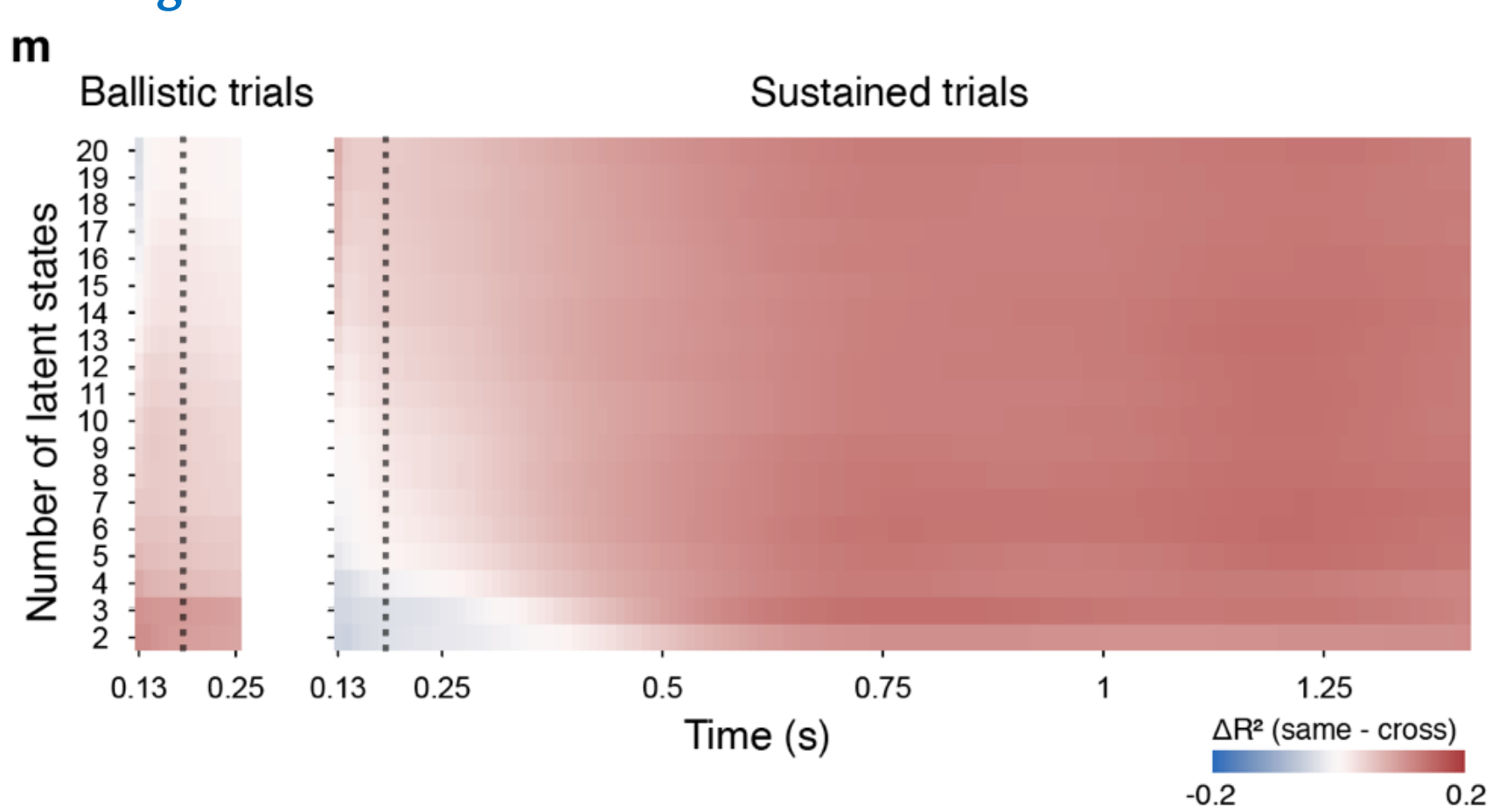

New Figure 3n:

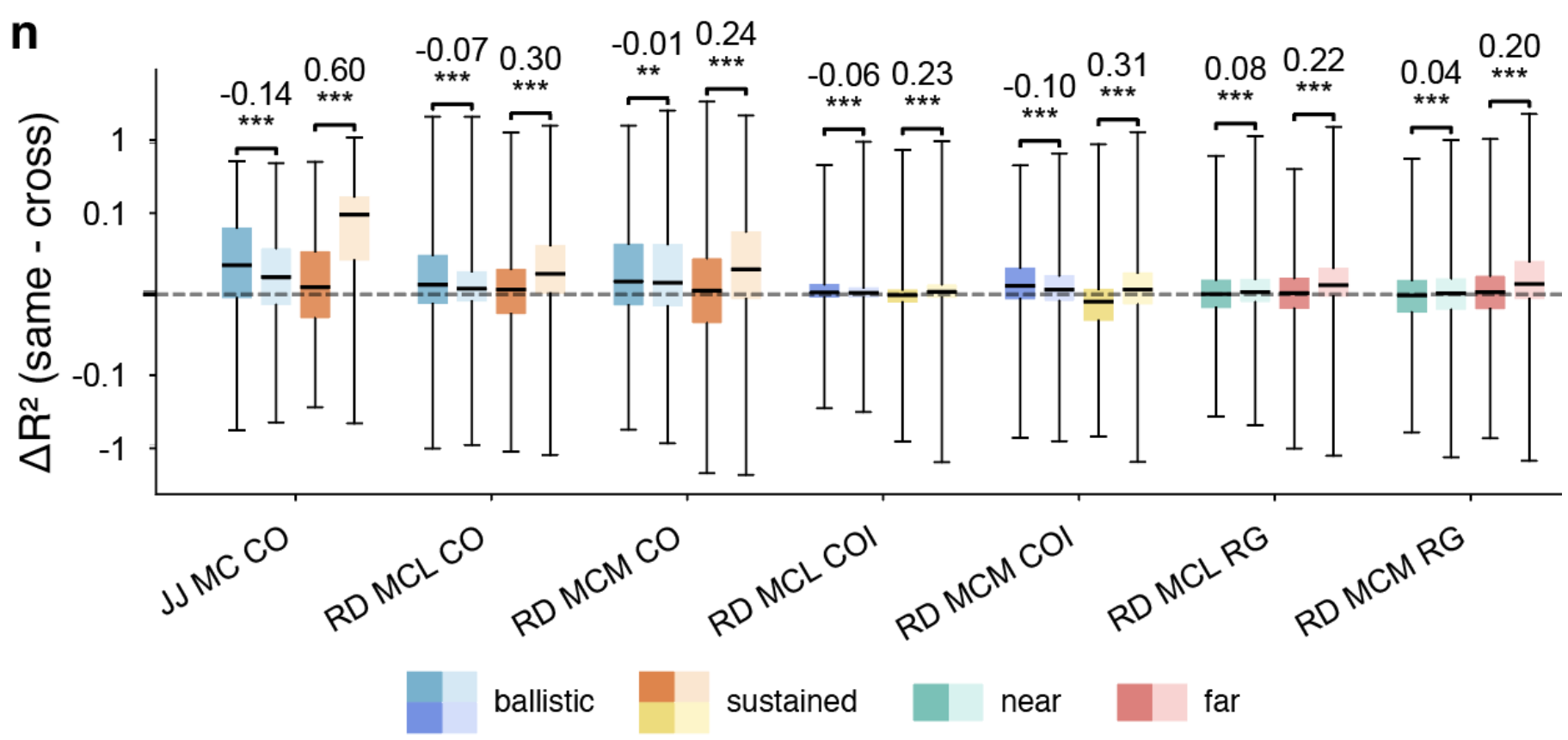

New Figure S6:

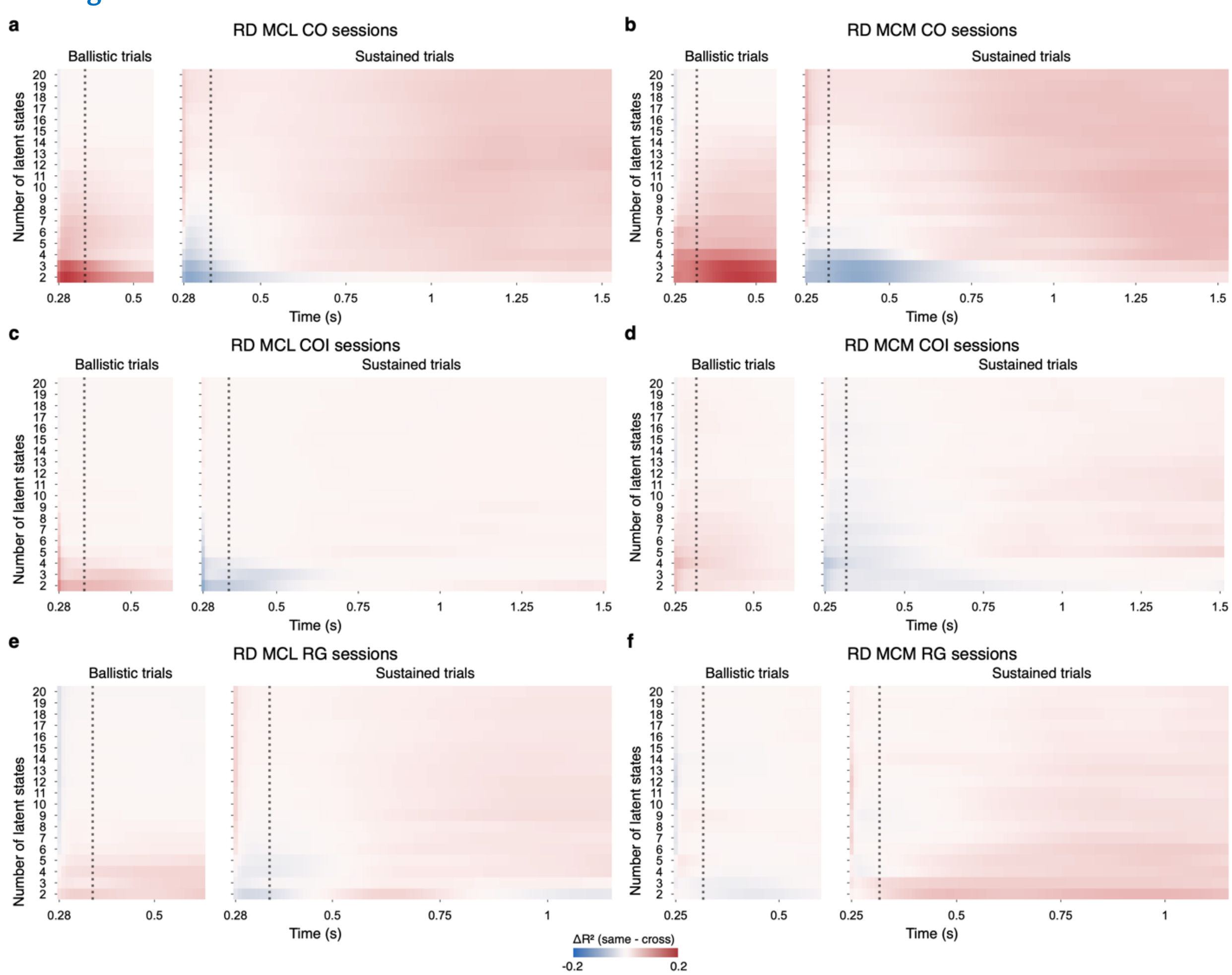

Figure 5 (rSLDS; Discrete States; Flow Fields)

10. Phase segmentation vs. autonomy.

rSLDS nicely reveals a transient to steady segmentation and reduced flow field magnitudes in the steady state. However, the inference that residual discrepancies must be input driven is too strong

given model choices. With limited discrete states and a fixed emission structure, rSLDS may underspecify smooth nonlinear attractors; failure of higher D models to find stable states does not disprove nonlinear autonomy. Please temper claims to "consistent with input driven modulation" rather than a categorical rejection of autonomous nonlinear explanations.

> We agree that rSLDS, while approximating a broad family of nonlinear autonomous systems via piecewise-linear segments, does not exhaust all possible nonlinear formulations. We have revised the language in **Results Section 2.5 (lines 300-306, 371-374)** accordingly, replacing categorical phrasing such as "must be captured by external inputs" with "consistent with modulation by external inputs," and adding an explicit acknowledgment that rSLDS approximates but does not exhaust all nonlinear autonomous systems.

11. Link to "intent": steady phase latent structure.
To substantiate that the steady phase carry's goal directed control, add a steady phase PCA/dPCA or a simple "progress to target" neural axis (ridge readout of cursor error), and plot its trajectory in the last 200–300 ms. This would demonstrate explicit motor intent when rotational/transient signatures have subsided.

> We thank the reviewer for this suggestion, which closely parallels their earlier request regarding Figure 2. As described in our response to the Figure 2 comment above, we have added two new panels **(Figure 2i,j)** that directly address this concern. Figure 2i shows PCA trajectories of MC population activity restricted to the final 300 ms before target acquisition in sustained trials, revealing clear target-separable clustering in the top two principal components. Figure 2j shows that an LDA classifier trained on time-averaged firing rates from this window decodes target direction well above chance across participants and tasks.
>
> Because the rSLDS latent trajectories (Figure 4) are derived from the same MC firing rates, the steady-phase attractor identified by the switching model corresponds to the same structured, goal-directed neural representation demonstrated in Figure 2i,j. We have added an explicit cross-reference in the rSLDS results section **(Section 2.5, lines 324-330)** to make this connection clear. Together, these analyses confirm that when transient signatures have subsided, motor cortex activity in the steady phase maintains an active, target-specific representation of motor intent consistent with ongoing input-driven control rather than neural idling.
>
> We also note that the reviewer's phrasing "rotational/transient signatures" implies that rotational structure is linked to the transient phase specifically. Our phase-specific rotational analyses (Figure 4e,f) do not support this pairing: the skew-symmetric ratio and dominant

angular velocity showed no consistent phase-dependent shift across sessions, participants, or tasks. The transient-to-steady transition is better characterized by a change in the rate of state evolution than by a collapse of rotational dynamics **(Results Section 2.5, lines 341-345)**. We have revised the language in the **Discussion (Section 3.1, lines 436-441)** to avoid inadvertently reinforcing this association.

12. Minor Points & Presentation

The term “pause” seems misleading since the neural activity is assumed to be structured, not silent. The authors could consider consider “low velocity steady state.” Instead.
Some figure elements are dense; adding schematics and consistent color/label keys per phase/condition would help. (In Fig 1 the brain schematics appeared inverted; please check figure orientation.)

"Pause" terminology. We agree that "pause" is misleading. We have replaced it with "low-velocity steady state" **(Abstract line 31; Introduction line 112)**.

Figure density and color/label consistency. We have revised the color scheme in **Figure 2** to remove non-meaningful color distinctions. Regarding schematics and consistent color/label keys: could the reviewer clarify what specific elements they find lacking? The phase definitions (transient, steady) and condition labels (ballistic, sustained) are described in each figure caption, but we are happy to add a shared visual legend or schematic if the reviewer can point to specific panels where the conventions are unclear.

New Figure 2:

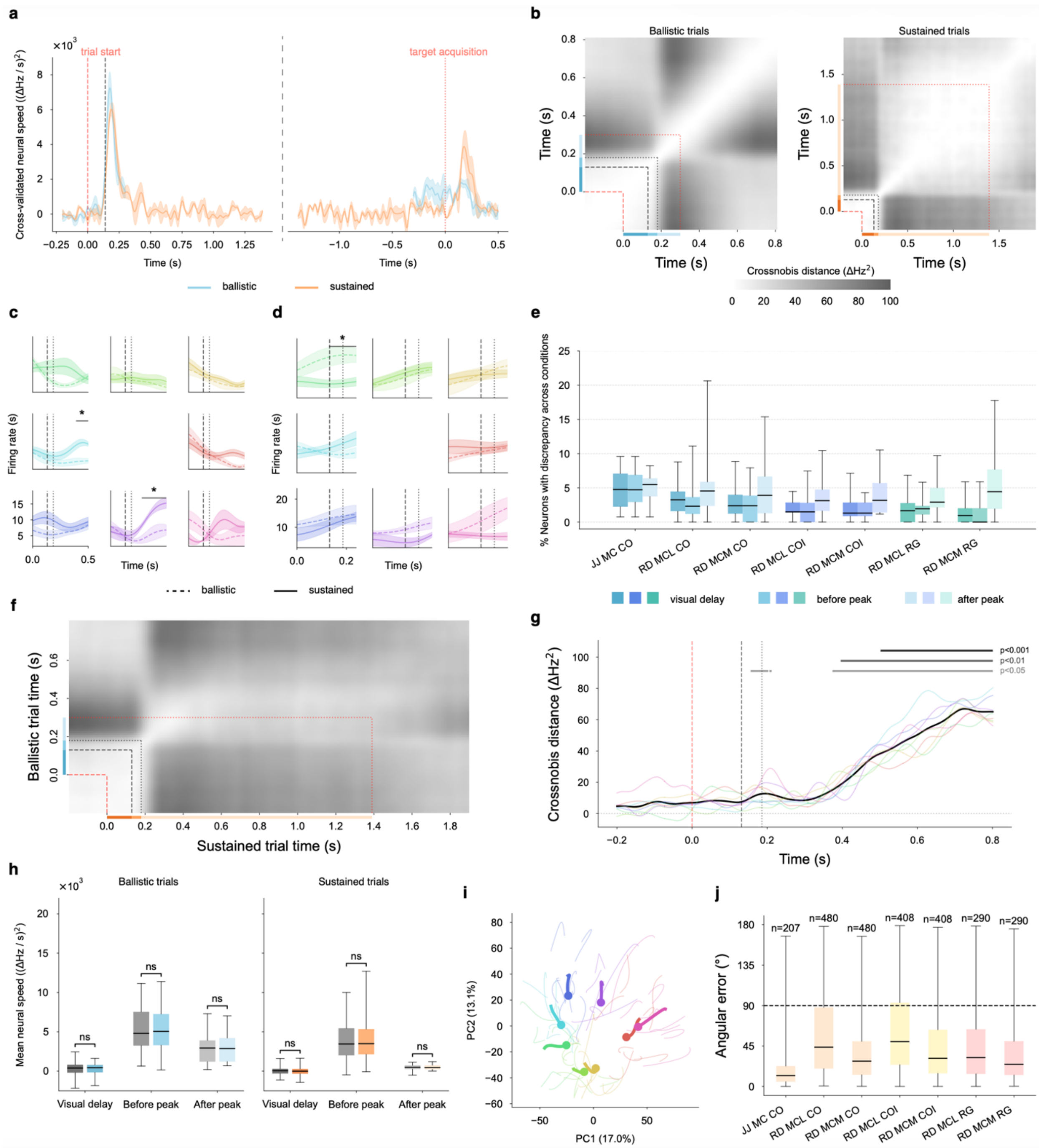

Brain schematic orientation (Figure 1). We have reviewed the brain schematics in Figure 1 and believe the orientation is correct. Could the reviewer clarify which specific element appears inverted? We will adjust accordingly if a specific concern is identified.

Response to Reviewer Comments

We thank all three reviewers for their constructive feedback throughout the review process. Reviewers #1, #2, and #3 each indicated they had no further requests for changes. We are grateful for their time and thoughtful engagement, which has substantially improved the manuscript.
