# Supplementary material for: Duration-modulated neural population dynamics in humans during BMI controls
Source: Commun Biol. 2026 Jun 26;9:1354. doi: 10.1038/s42003-026-10369-8 (PMC13669311; doi:10.1038/s42003-026-10369-8)
Supplement: Supplementary file 2 [file 42003_2026_10369_MOESM2_ESM.pdf]

Supplementary Information

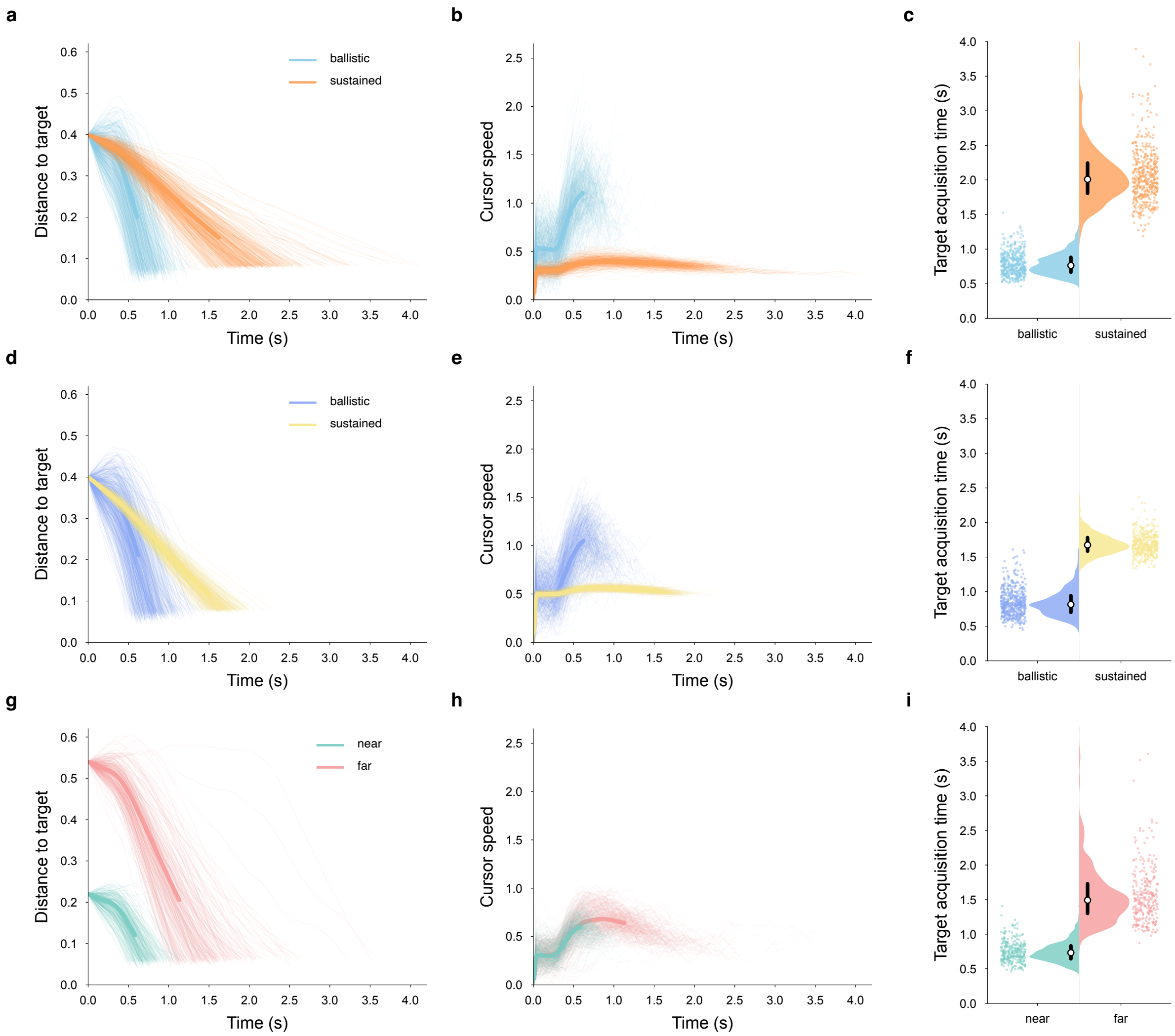

Supplementary Figure 1. Behavioral data across Participant RD's center-out, interleaved center-out, and radial-grid sessions. a,d,g, Distance to target over time for Participant RD's center-out task (**a**; $n = 473$ ballistic and $n = 480$ sustained trials, pooled across 6 sessions), interleaved center-out task (**d**; $n = 528$ ballistic and $n = 408$ sustained trials, pooled across 3 sessions), and radial-grid task (**g**; $n = 282$ near and $n = 290$ far trials, pooled across 3 sessions). Each light-colored trace represents a trial. The dark colored traces represent condition averages. **b,e,h**, Cursor speed over time for Participant RD's center-out task (**b**), interleaved center-out task (**e**), and radial-grid task (**h**) trials (same trials as in **a,d,g**, respectively). Each light-colored trace represents a trial. The dark colored traces represent condition averages. **c,f,i**, Target acquisition times for Participant RD's center-out task (**c**), interleaved center-out task (**f**), and radial-grid task (**i**) trials (same trials as in **a,d,g**, respectively). Each half-violin shows the condition-specific kernel density estimate of the distribution across all trials. The central vertical bar spans the interquartile range (25th–75th percentile); the white circle indicates the median. Individual trials are shown as dots.

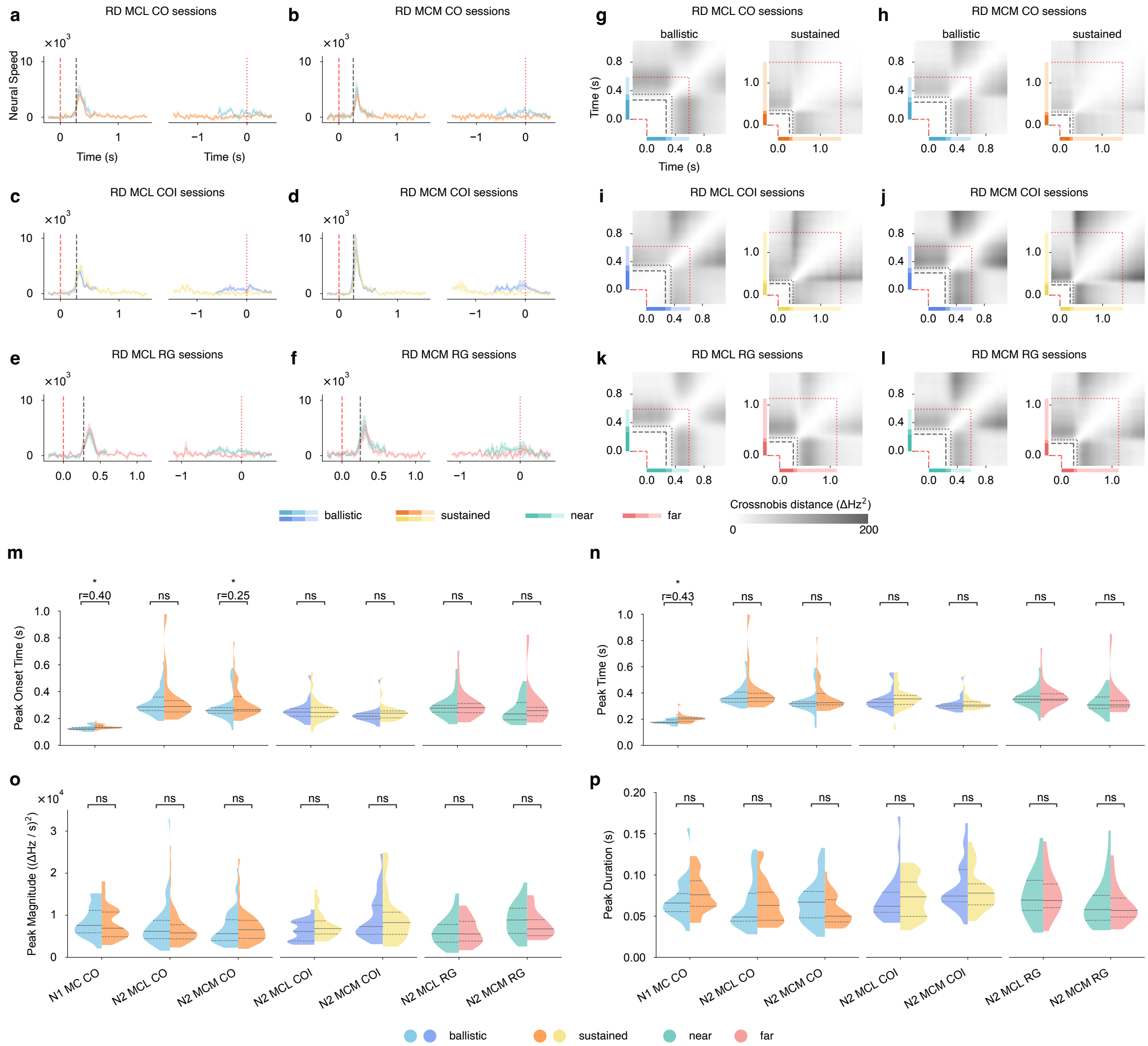

Supplementary Figure 2. Cross-validated neural speed, crossnobis distance, and peak statistics across all sessions, participants, and tasks. a-f, Cross-validated neural speed during trials under different conditions in Participant RD's center-out task (**a,b**; $n = 426$ ballistic and $n = 429$ sustained trials), interleaved center-out task (**c,d**; $n = 476$ ballistic and $n = 366$ sustained trials), and radial-grid task (**e,f**; $n = 252$ near and $n = 260$ far trials) sessions. Trials of each condition are truncated to the shortest trial duration observed for that condition. The error bands show 95% confidence intervals across trials. Left (in each panel): Trials are aligned to the start time marked by the red dashed line. The black dashed line (left) marks the peak onset time (see Methods). Data includes 200 ms before trial start. Right (in each panel): Trials are aligned to the target acquisition time marked by the red dotted line. Data includes 500 ms after target acquisition. **g-l**, Crossnobis distance across time during trials under different conditions in Participant RD's center-out task (**g,h**), interleaved center-out task (**i,j**), and radial-grid task (**k,l**) sessions. Same trials as in **a-f** are used. Data includes 200 ms before trial start and 500 ms after target acquisition. Trials of each condition are truncated to the shortest trial duration observed for that condition. The red dashed lines mark the trial start time. The red dotted lines mark the target acquisition time of the shortest trial of each condition. The black dashed lines and dotted lines mark the peak onset times and peak times (see Methods), respectively. The horizontal and vertical bars beside axis ticks mark the phases within each trial. Lighter to darker shades of each color correspond to the visual delay (see Methods), before-peak, and after-peak windows, respectively. **m-p**, Peak onset time (**m**), peak time (**n**), peak magnitude (**o**), and peak duration (**p**) of cross-validated neural speed for all sessions, participants, brain areas, and task conditions. Participant JJ results summarize $n = 162$ ballistic $n = 186$ sustained trials. Participant RD results come from the same trials as in **a-f**. Significance between conditions for each target-task pair is shown above each pair of violins (Mann-Whitney U test, $^*p < 0.05$, ns: not significant). Effect size (rank-biserial r) is shown for significant pairs.

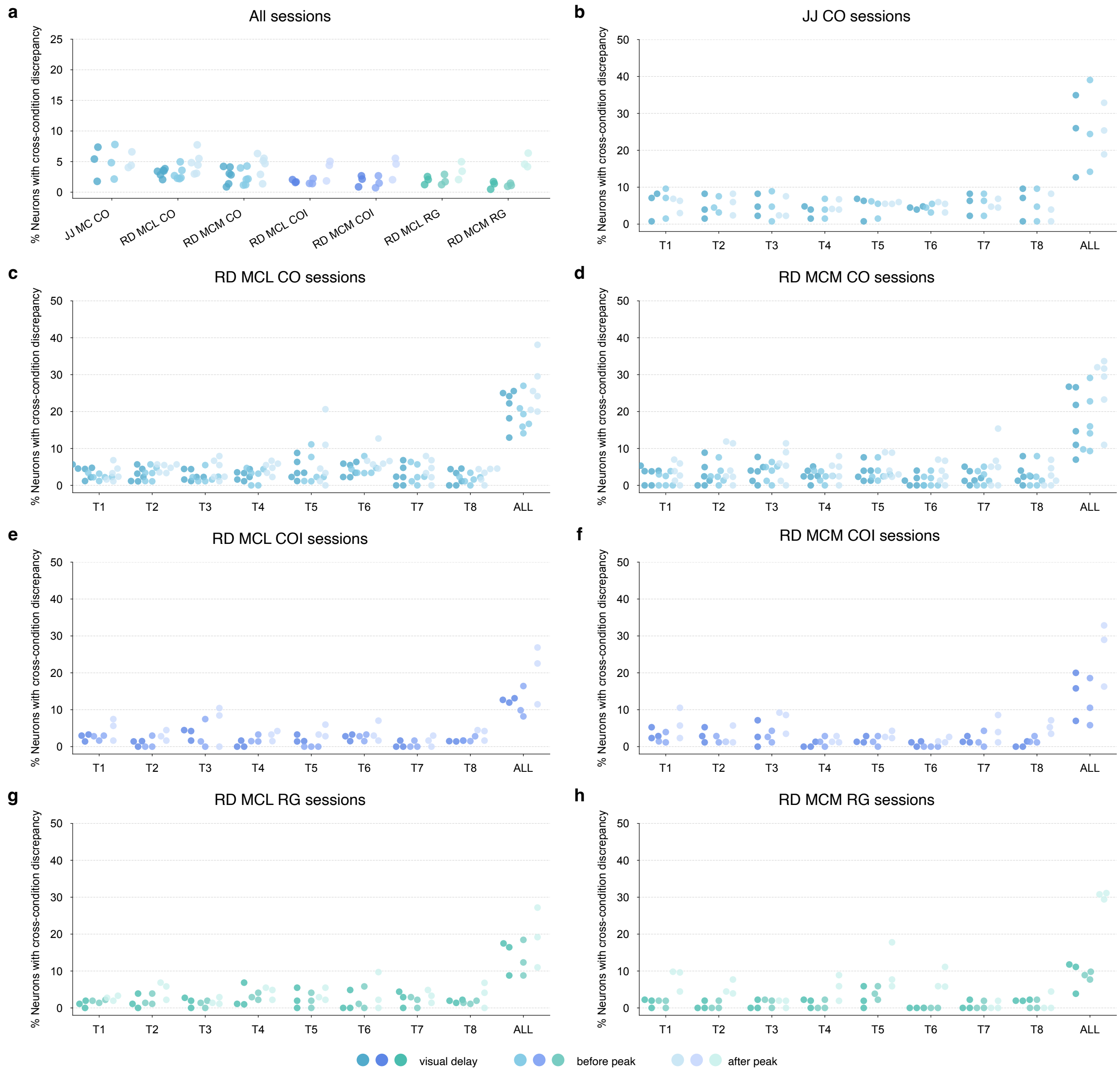

Supplementary Figure 3. Percent of neurons with significant cross-condition discrepancy, broken down by target direction and movement epoch. Blues, indigos, and greens indicate the center-out, interleaved center-out, and radial-grid tasks, respectively. Within each task color, lighter to darker shades indicate the visual-delay, before-peak, and after-peak epochs (see Methods). **a**, Cross-session summary. Each group on the horizontal axis is a subject–task combination. Each dot represents one session's mean cross-condition discrepancy averaged across target directions for a given epoch. **b-h**, Per-session results. For each target direction, dots show the percentage of neurons with cross-condition discrepancy in each epoch and each session, yielding $n_{\text{sessions}} \times 3$ dots per target column. The ALL column reports the fraction of neurons showing cross-condition discrepancy toward at least one target direction. **b**, Results from Participant JJ's center-out sessions ($n = 3$). **c,d**, Results from Participant RD's center-out sessions ($n = 6$). **e,f**, Results from Participant RD's interleaved center-out sessions ($n = 3$). **g,h**, Results from Participant RD's radial-grid sessions ($n = 3$).

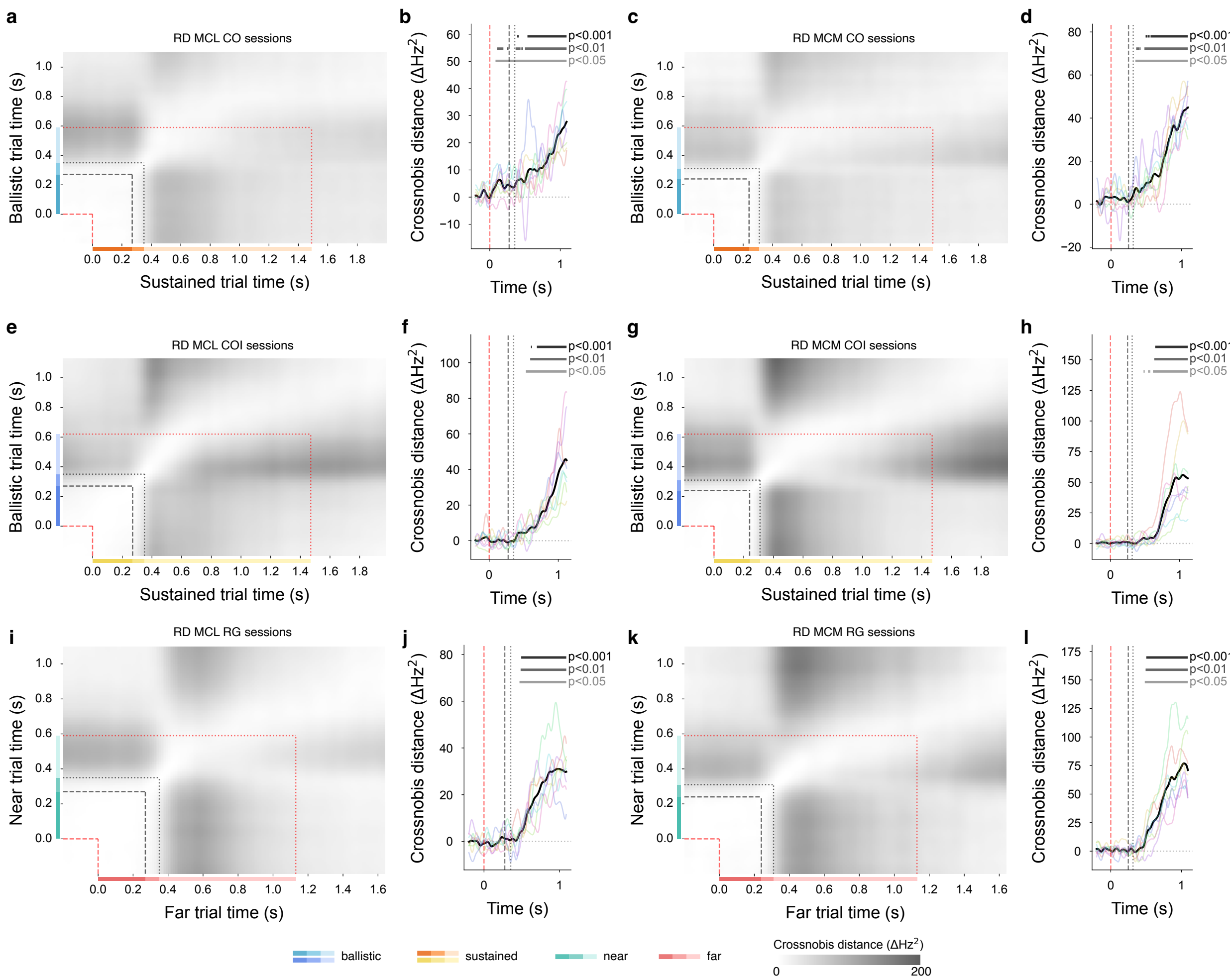

Supplementary Figure 4. Cross-condition crossnobis distance across Participant RD's center-out, interleaved center-out, and radial-grid sessions. **a,c,e,g,i,k**, Crossnobis distance across time between trials of different conditions in Participant RD's center-out (**a,c**; $n = 426$ ballistic and $n = 429$ sustained trials), interleaved center-out (**e,g**; $n = 476$ ballistic and $n = 366$ sustained trials), and radial-grid (**i,k**; $n = 252$ near and $n = 260$ far trials) tasks. Data includes 200 ms before trial start and 500 ms after target acquisition. Trials of each condition are truncated to the shortest trial duration observed for that condition. Red dashed lines mark the trial start time. Red dotted lines mark the target acquisition time of the shortest trial of each condition. Black dashed lines and dotted lines mark the peak onset times and peak times (see Methods), respectively. Horizontal and vertical bars beside axis ticks mark phases within each trial. Lighter to darker shades of each color correspond to visual delay (see Methods), before-peak, and after-peak windows, respectively. **b,d,f,h,j,l**, Cross-condition crossnobis distance along the diagonal (same time point across conditions) for trials of different conditions in Participant RD's center-out (**b,d**), interleaved center-out (**f,h**), and radial-grid (**j,l**) sessions. Same data are used as in **a,c,e,g,i,k**. The black trace shows the mean across sessions and target directions. Thin colored traces show per-target averages. Horizontal bars above the traces indicate time bins where the crossnobis distance is significantly greater than the pre-movement baseline (Mann-Whitney U test, FDR-corrected; bar shading indicates $p < 0.05$, $p < 0.01$, and $p < 0.001$). Vertical lines follow the conventions in the crossnobis distance plots (**a,c,e,g,i,k**).

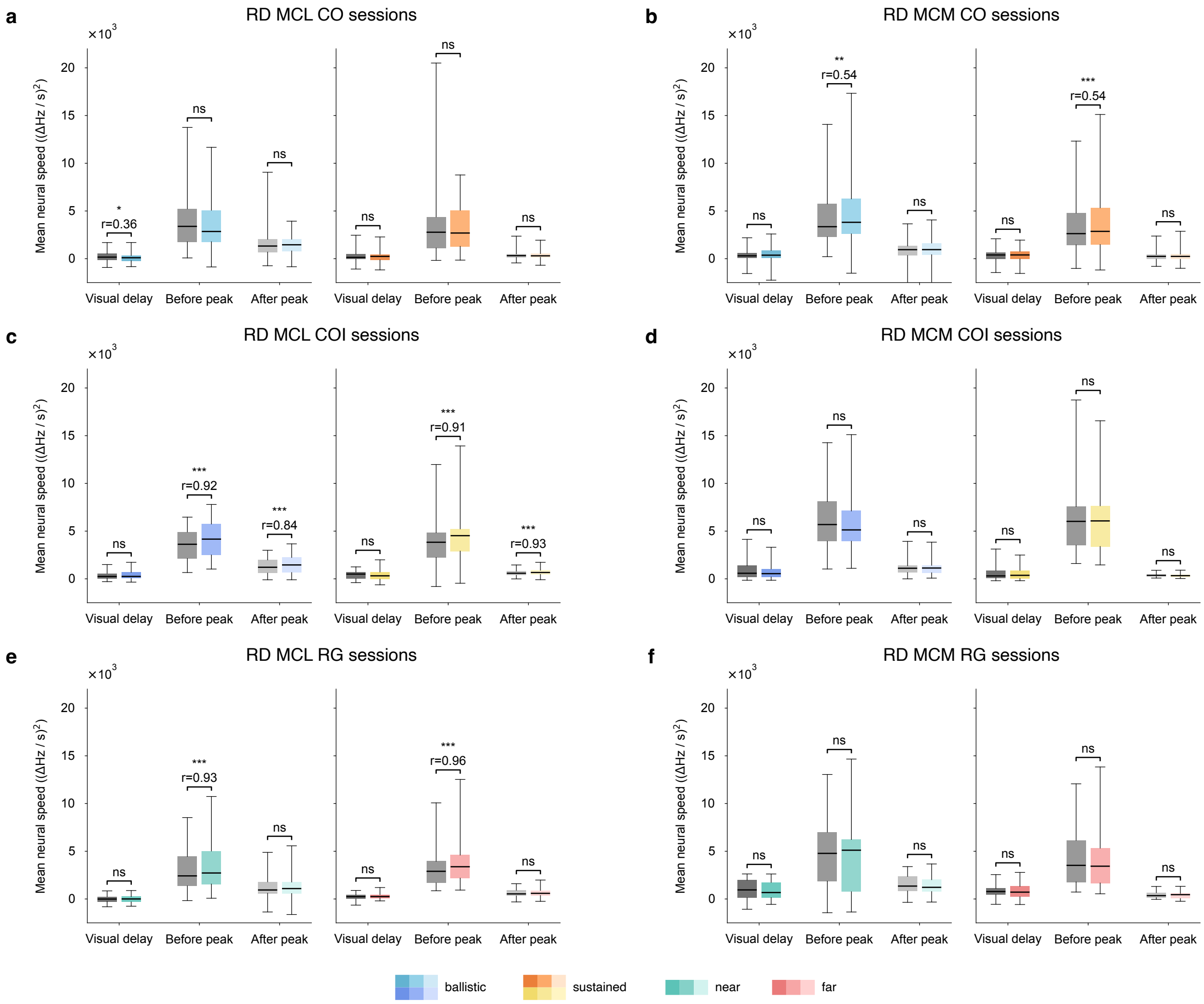

Supplementary Figure 5. Robustness check for within-condition crossnobis distance after removing neurons with significant firing rate differences between trials of different conditions. For each neuron and target direction, a Mann-Whitney U test was applied at each time bin spanning the visual delay, before-peak, and after-peak epochs; FDR correction was applied jointly across all movement-epoch bins. Neurons significant in any movement epoch or target direction were removed. Bar plots show neural speed averaged within each movement epoch (visual delay, before-peak, after-peak) before (gray) and after (condition color) neuron removal. Error bars indicate s.e.m. Brackets indicate paired Wilcoxon signed-rank tests between the original and reduced neural populations (*$p < 0.05$, **$p < 0.01$, ***$p < 0.001$, ns: not significant). Effect size (rank-biserial r) is shown for significant pairs. **a,b**, Results from Participant RD's center-out sessions ($n = 6$ sessions; $n = 426$ ballistic and $n = 429$ sustained trials). **c,d**, Results from Participant RD's interleaved center-out sessions ($n = 3$ sessions; $n = 476$ ballistic and $n = 366$ sustained trials). **e,f**, Results from Participant RD's radial-grid sessions ($n = 3$ sessions; $n = 252$ near and $n = 260$ far trials).

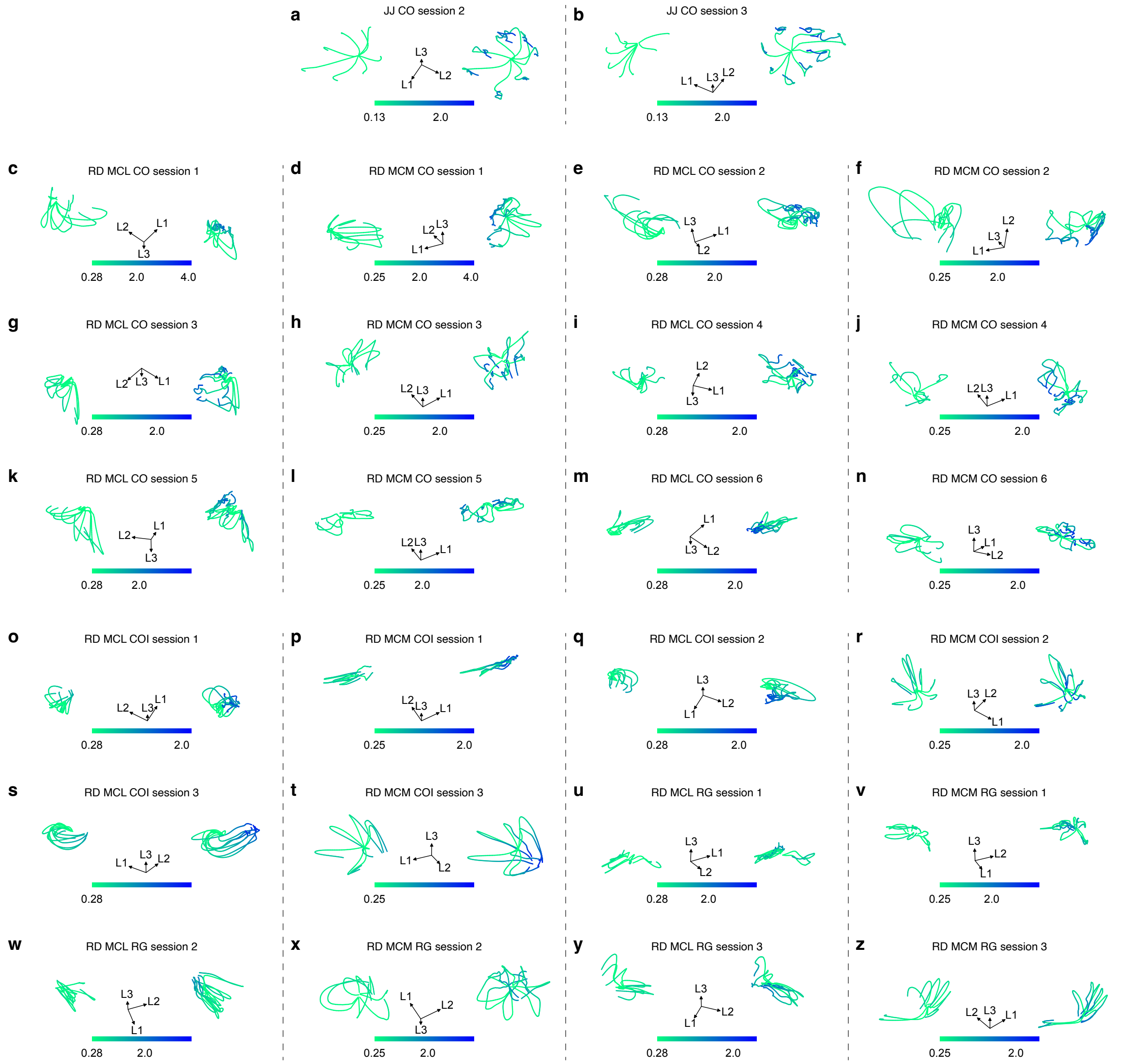

Supplementary Figure 6. LDS latent neural trajectories color-coded by time since the start of trials. The trajectories show target averages after time-resampling. **a,b**, Results from Participant JJ's center-out sessions. **c-n**, Results from Participant RD's center-out sessions. **o-t**, Results from Participant RD's interleaved center-out sessions. **u-z**, Results from Participant RD's radial-grid sessions.

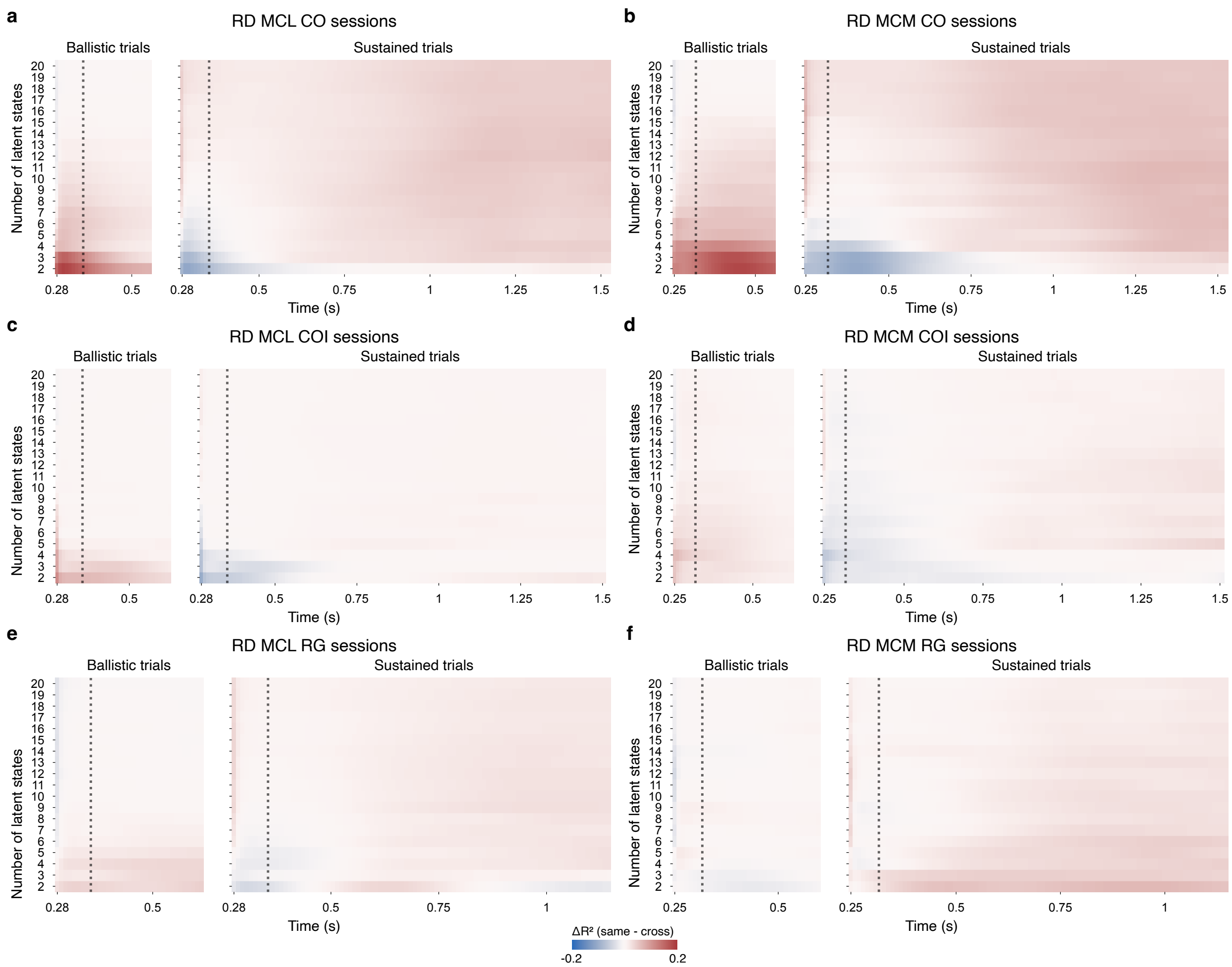

Supplementary Figure 7. Difference in inference performance between same-condition and cross-condition models for participant RD's sessions. Positive values (red) indicate that the same-condition model forecasts neural dynamics more accurately than the cross-condition model; negative values (blue) indicate the reverse. For each combination of task and brain area, results are averaged over trials pool from all applicable sessions and 5 cross-validation folds. The width of each heatmap is proportional to trial duration. Dotted vertical line indicates peak time where the neural speed is highest (see Methods). **a,b**, Results from Participant RD's center-out sessions ($n = 6$ sessions; $n = 426$ ballistic and $n = 429$ sustained trials). **c,d**, Results from Participant RD's interleaved center-out sessions ($n = 3$ sessions; $n = 476$ ballistic and $n = 366$ sustained trials). **e,f**, Results from Participant RD's radial-grid sessions ($n = 3$ sessions; $n = 252$ near and $n = 260$ far trials).

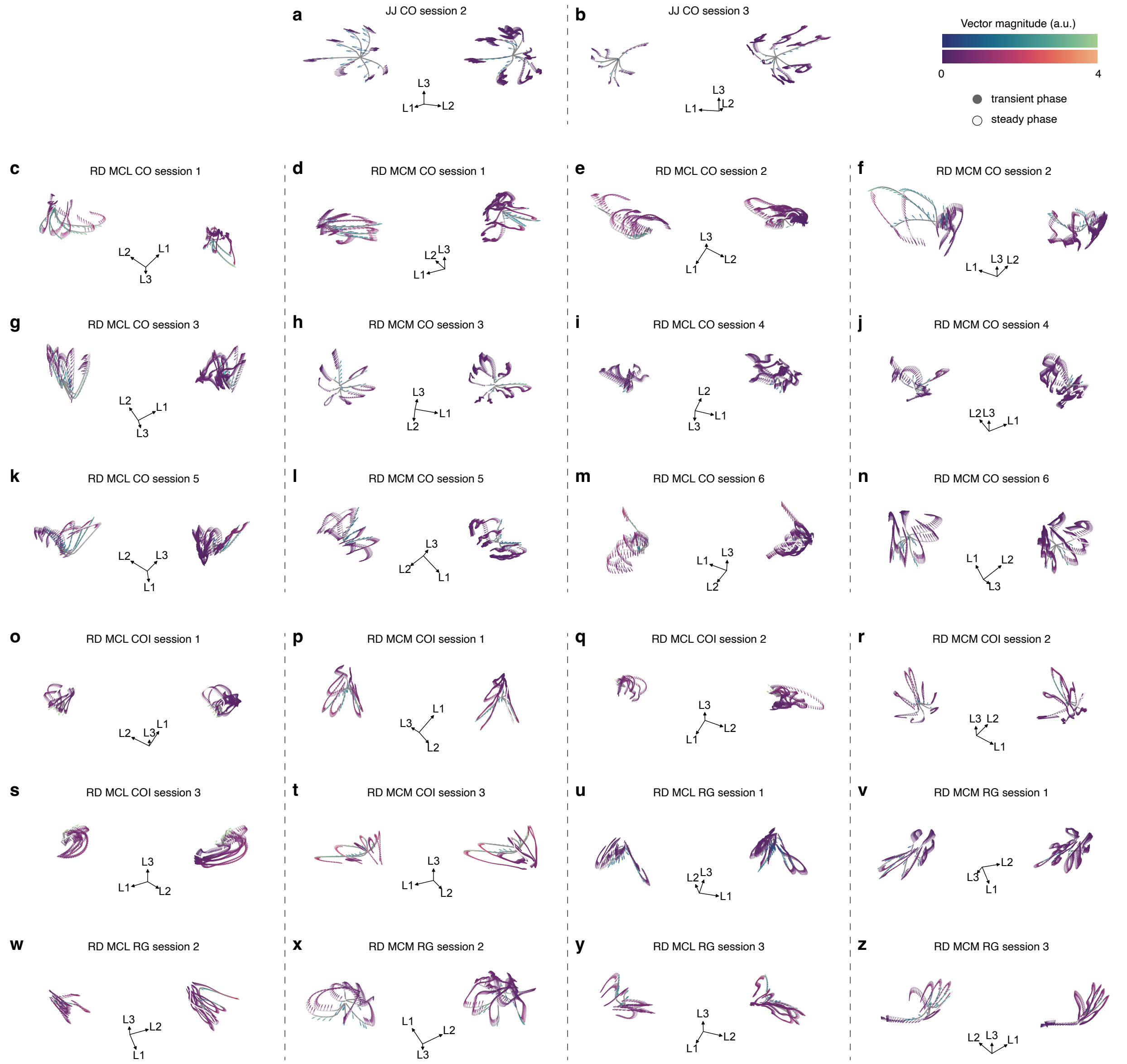

Supplementary Figure 8. rSLDS (C3D2) latent neural trajectories color-coded by discrete states. The trajectories show target averages after time-resampling. Flow field vectors learned by rSLDS are overlaid on the trajectories. Transient phase and steady phase flow field vectors are represented by cold and warm color maps, respectively. **a,b**, Results from Participant JJ's center-out sessions. **c-n**, Results from Participant RD's center-out sessions. **o-t**, Results from Participant RD's interleaved center-out sessions. **u-z**, Results from Participant RD's radial-grid sessions.

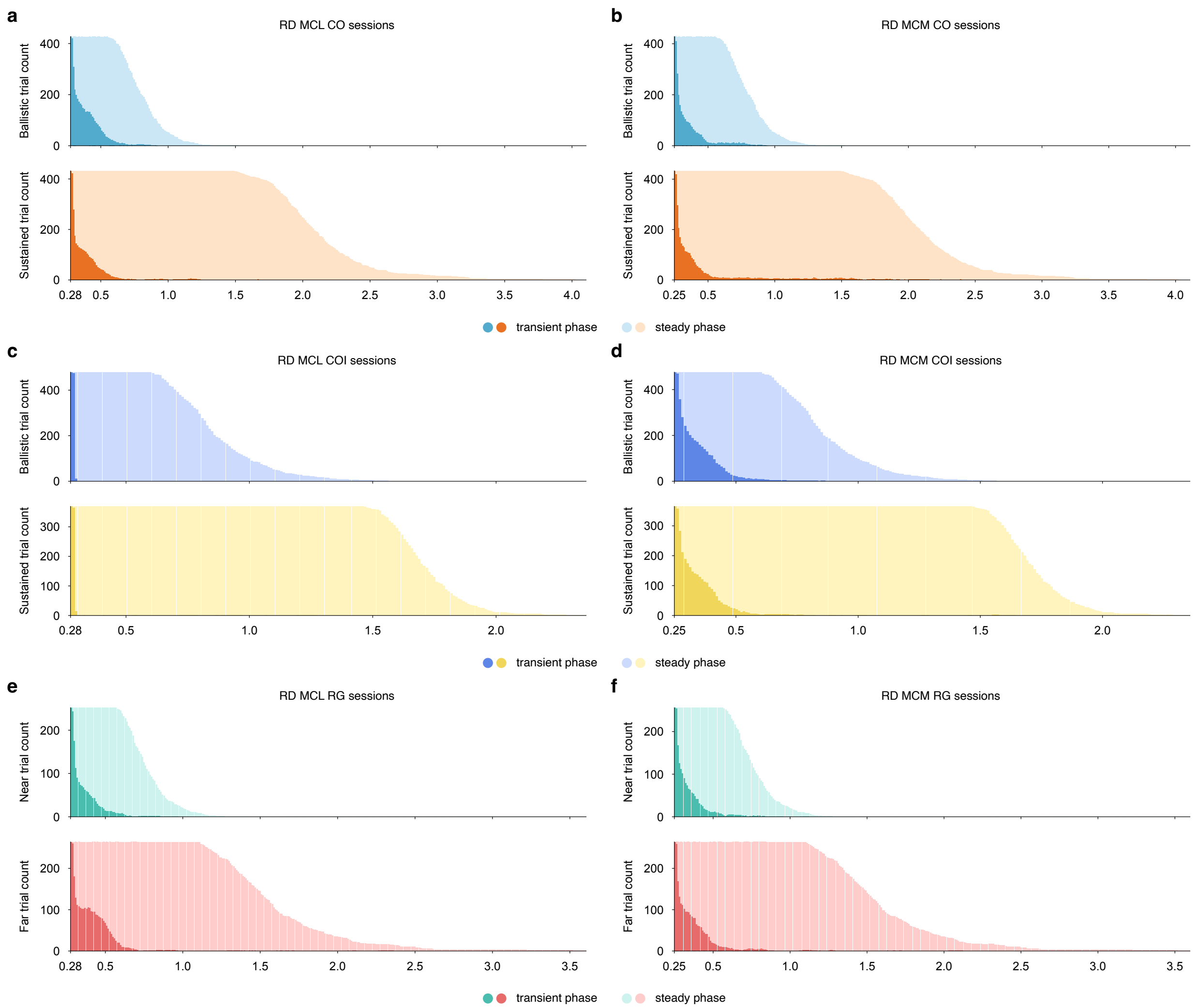

Supplementary Figure 9. Trial counts of rSLDS (C3D2) discrete states over time grouped by task condition. a,b, Results from Participant RD's center-out sessions ($n = 6$ sessions; $n = 426$ ballistic and $n = 429$ sustained trials). **c,d**, Results from Participant RD's interleaved center-out sessions ($n = 3$ sessions; $n = 476$ ballistic and $n = 366$ sustained trials). **e,f**, Results from Participant RD's radial-grid sessions ($n = 3$ sessions; $n = 252$ near and $n = 260$ far trials).

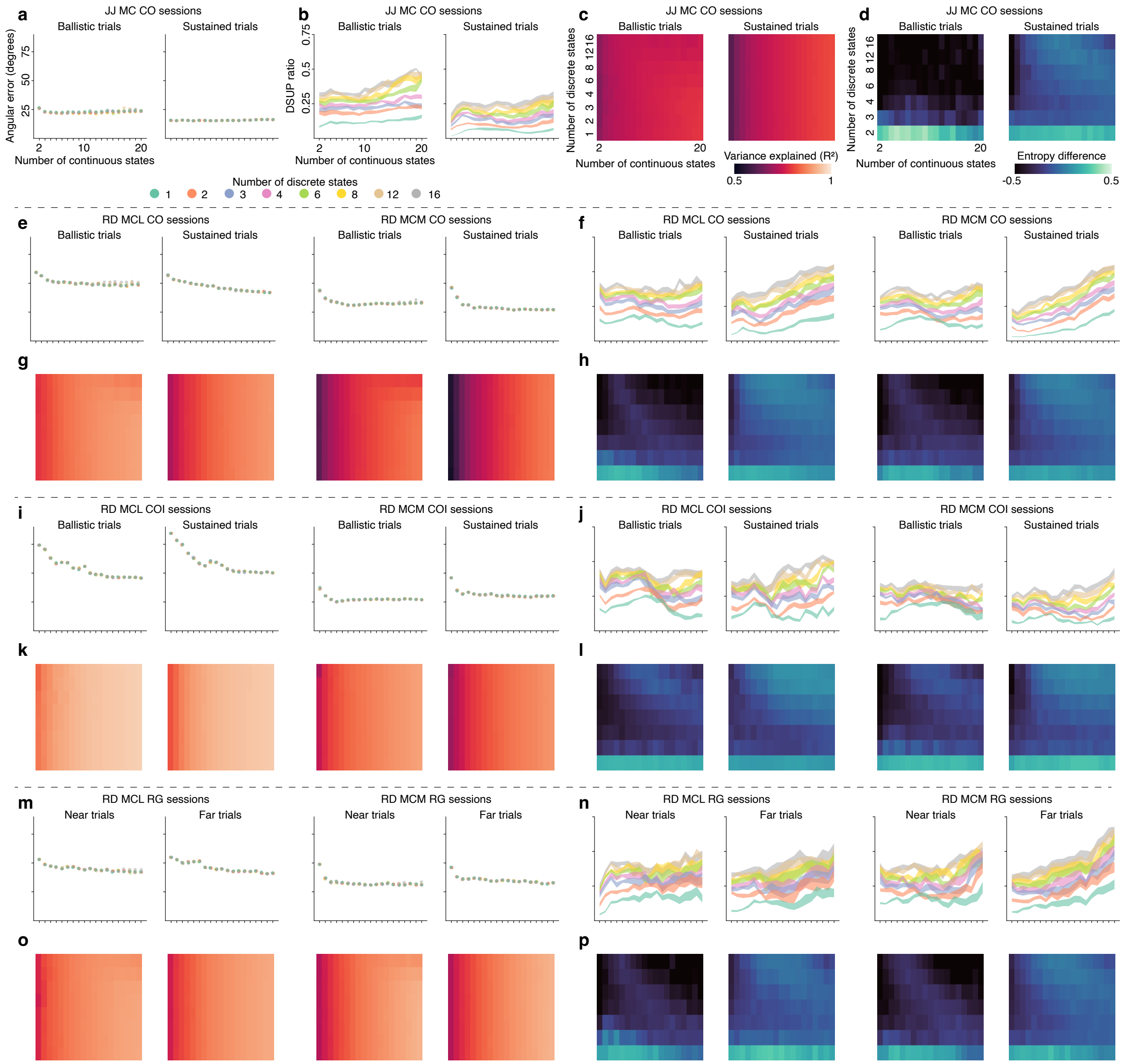

Supplementary Figure 10. Performance of LDS and rSLDS on position-error decoding, DSUP ratio, inference, and dimensional entropy difference (see Methods), swept over continuous- and discrete-state counts. The continuous-state counts are C $\in \{2, 3, ..., 20\}$. The discrete-state counts are D $\in \{1, 2, 3, 4, 6, 8, 12, 16\}$. Results are organized by participant, brain area, and task. For each combination of participant, brain area, and task, results are averaged over trials pool from all applicable sessions and 5 cross-validation folds. There are $n = 162$ ballistic and $n = 186$ sustained trials for Participant JJ's center-out task, $n = 426$ ballistic and $n = 429$ sustained trials for Participant RD's center-out task, $n = 476$ ballistic and $n = 366$ sustained trials for Participant RD's interleaved center-out task, and $n = 252$ near and $n = 260$ far trials for Participant RD's radial-grid task. **a,e,i,m**, Position-error decoding results. **b,f,j,n**, DSUP ratio results. **c,g,k,o**, Inference results. **d,h,l,p**, Dimensional entropy difference results.

Supplementary Table 1. Summary of data used across all sessions. # Trials 1 and # Trials 2 denote the number of trials retained for each condition, with the percentage discarded in parentheses. Tasks: CO, RG, and COI denote center-out, radial-grid, and center-out interleaved, respectively. Array locations: MCL and MCM denote MC-LAT and MC-MED, respectively.

Session	Participant	Task	Array(s)	# Neurons	Conditions	# Trials 1 (% disc.)	# Trials 2 (% disc.)
20190412	JJ	CO	MC	134	ballistic, sustained	58 (9.38)	58 (9.38)
20190517	JJ	CO	MC	127	ballistic, sustained	58 (27.50)	71 (11.25)
20190528	JJ	CO	MC	146	ballistic, sustained	64 (33.33)	78 (18.75)
**JJ SUM**			**MC**	**407**		**180 (25.00)**	**207 (13.75)**
20240516	RD	CO	MCL, MCM	63, 78	ballistic, sustained	55 (14.06)	64 (0.00)
20240530	RD	CO	MCL, MCM	88, 43	ballistic, sustained	50 (21.88)	64 (0.00)
20240816	RD	CO	MCL, MCM	90, 79	ballistic, sustained	112 (11.81)	96 (0.00)
20240820	RD	CO	MCL, MCM	85, 82	ballistic, sustained	64 (33.33)	64 (0.00)
20241015	RD	CO	MCL, MCM	88, 101	ballistic, sustained	103 (19.53)	96 (0.00)
20241022	RD	CO	MCL, MCM	91, 75	ballistic, sustained	89 (7.29)	96 (0.00)
20241105	RD	RG	MCL, MCM	103, 51	near, far	93 (3.13)	92 (4.17)
20241211	RD	RG	MCL, MCM	91, 52	near, far	76 (5.00)	78 (2.50)
20250408	RD	RG	MCL, MCM	73, 45	near, far	113 (5.83)	120 (0.00)
20250417	RD	COI	MCL, MCM	71, 70	ballistic, sustained	189 (9.13)	152 (0.00)
20250422	RD	COI	MCL, MCM	61, 86	ballistic, sustained	175 (8.85)	112 (0.00)
20250509	RD	COI	MCL, MCM	67, 76	ballistic, sustained	164 (6.82)	144 (0.00)
**RD SUM**			**MCL, MCM**	**971, 838**		**1283 (11.33)**	**1178 (0.51)**
