# Supplementary material for: Duration-modulated neural population dynamics in humans during BMI controls
Source: Commun Biol. 2026 Jun 26;9:1354. doi: 10.1038/s42003-026-10369-8 (PMC13669311; doi:10.1038/s42003-026-10369-8)
Supplement: Supplementary file 3 [file 42003_2026_10369_MOESM3_ESM.pdf]

Description of Additional Supplementary Files

File name: Supplementary Data 1
Description: Example trials of Participant JJ performing the center-out task and Participant RD performing the radial-grid task.
