# Supplementary material for: Duration-modulated neural population dynamics in humans during BMI controls
Source: Commun Biol. 2026 Jun 26;9:1354. doi: 10.1038/s42003-026-10369-8 (PMC13669311; doi:10.1038/s42003-026-10369-8)
Supplement: Supplementary file 4 [file 42003_2026_10369_MOESM5_ESM.pdf]

nature portfolio

Corresponding author(s): Fei Yin and Richard Andersen

Last updated by author(s): Apr 29, 2026

Reporting Summary

Nature Portfolio wishes to improve the reproducibility of the work that we publish. This form provides structure for consistency and transparency in reporting. For further information on Nature Portfolio policies, see our Editorial Policies and the Editorial Policy Checklist.

Statistics

For all statistical analyses, confirm that the following items are present in the figure legend, table legend, main text, or Methods section.

n/a	Confirmed	
☐	☒	The exact sample size (*n*) for each experimental group/condition, given as a discrete number and unit of measurement
☐	☒	A statement on whether measurements were taken from distinct samples or whether the same sample was measured repeatedly
☐	☒	The statistical test(s) used AND whether they are one- or two-sided<br>*Only common tests should be described solely by name; describe more complex techniques in the Methods section.*
☐	☒	A description of all covariates tested
☐	☒	A description of any assumptions or corrections, such as tests of normality and adjustment for multiple comparisons
☐	☒	A full description of the statistical parameters including central tendency (e.g. means) or other basic estimates (e.g. regression coefficient) AND variation (e.g. standard deviation) or associated estimates of uncertainty (e.g. confidence intervals)
☐	☒	For null hypothesis testing, the test statistic (e.g. F, t, r) with confidence intervals, effect sizes, degrees of freedom and P value noted<br>*Give P values as exact values whenever suitable.*
☒	☐	For Bayesian analysis, information on the choice of priors and Markov chain Monte Carlo settings
☒	☐	For hierarchical and complex designs, identification of the appropriate level for tests and full reporting of outcomes
☐	☒	Estimates of effect sizes (e.g. Cohen's *d*, Pearson's *r*), indicating how they were calculated

Our web collection on statistics for biologists contains articles on many of the points above.

Software and code

Policy information about availability of computer code

Data collection	Data were collected using the NeuroPort system from Blackrock Neurotech.
Data analysis	Matlab R2023b, Python 3.10.13

For manuscripts utilizing custom algorithms or software that are central to the research but not yet described in published literature, software must be made available to editors and reviewers. We strongly encourage code deposition in a community repository (e.g. GitHub). See the Nature Portfolio guidelines for submitting code & software for further information.

Data

Policy information about availability of data

All manuscripts must include a data availability statement. This statement should provide the following information, where applicable:

- Accession codes, unique identifiers, or web links for publicly available datasets
- A description of any restrictions on data availability
- For clinical datasets or third party data, please ensure that the statement adheres to our policy

The data supporting the findings of this study are openly available via Zenodo at https://doi.org/10.5281/zenodo.19445138. The code developed for this study is openly available via GitHub at https://github.com/ynffsy/DurationModulatedDynamics.

Research involving human participants, their data, or biological material

Policy information about studies with human participants or human data. See also policy information about sex, gender (identity/presentation), and sexual orientation and race, ethnicity and racism.

Reporting on sex and gender	Neural data were recorded from participants: JJ, a male in his 50s; RD, a male in his 40s.
Reporting on race, ethnicity, or other socially relevant groupings	The study design did not consider race, ethnicity and other socially relevant groupings.
Population characteristics	Participant JJ is tetraplegic (C5-C6); Participant RD is tetraplegic (C3-C4).
Recruitment	Participants were recruited through our collaborating hospitals according to IRB approved inclusion and exclusion criterion.
Ethics oversight	The study and all procedures were approved by the Institutional Review Boards of the California Institute of Technology, the University of California, Los Angeles, Casa Colina Hospital and Centers for Healthcare, and the University of Southern California.

Note that full information on the approval of the study protocol must also be provided in the manuscript.

Field-specific reporting

Please select the one below that is the best fit for your research. If you are not sure, read the appropriate sections before making your selection.

☐ Life sciences ☒ Behavioural & social sciences ☐ Ecological, evolutionary & environmental sciences

For a reference copy of the document with all sections, see nature.com/documents/nr-reporting-summary-flat.pdf

Behavioural & social sciences study design

All studies must disclose on these points even when the disclosure is negative.

Study description	Data were quantitative experimental. Neuronal firing rates were recorded while each participant performed variations of cursor control tasks with a brain-machine interface. Recorded data were analyzed offline.
Research sample	Two human participants affected by tetraplegia performed cursor control tasks with brain-machine interfaces. For Participant JJ, an average of 136 sorted MC units (between 127–146) were recorded per session. For Participant RD, an average of 81 sorted lateral MC units (between 61-103) and 70 sorted medial MC units (between 43-101) were recorded per session. For Participant JJ, data were collected over 3 study sessions, with a total of 387 trials. Two experimental conditions were run, a ballistic condition and a sustained condition. The two conditions are block-interleaved. For Participant 2, data were collected over 12 sessions, with a total of 2461 trials. Four experimental conditions were run, including ballistic, sustained, near, and far. The near and far conditions were trial-interleaved. For ballistic and sustained conditions, both trial-interleaving and block-interleaving were explored.
Sampling strategy	Study sessions were repeated on different session days. On all session days, the participants were able to control the cursor effectively. The number of trials per session were not predetermined.
Data collection	Data were recorded with Blackrock NeuroPort arrays. Blackrock neural signal processor system and headstages were used. Besides the participant and researcher, the participant's caretaker or hospital staffs were present during all data collection. Blindness was not applicable.
Timing	For Participant JJ, data were collected between April 2019 and May 2019. For Participant RD, data were collected between May 2024 and May 2025.
Data exclusions	Trials where cursor trajectories deviated significantly from the shortest trajectories between starting positions and targets were excluded.
Non-participation	No participant dropped out.
Randomization	Participants were not allocated into experimental groups. In both participants, we evaluated neural population dynamics in the motor cortex.

Reporting for specific materials, systems and methods

We require information from authors about some types of materials, experimental systems and methods used in many studies. Here, indicate whether each material, system or method listed is relevant to your study. If you are not sure if a list item applies to your research, read the appropriate section before selecting a response.

Materials & experimental systems

n/a	Involved in the study
☒	☐ Antibodies
☒	☐ Eukaryotic cell lines
☒	☐ Palaeontology and archaeology
☒	☐ Animals and other organisms
☐	☒ Clinical data
☒	☐ Dual use research of concern
☒	☐ Plants

Methods

n/a	Involved in the study
☒	☐ ChIP-seq
☒	☐ Flow cytometry
☒	☐ MRI-based neuroimaging

Clinical data

Policy information about clinical studies

All manuscripts should comply with the ICMJE guidelines for publication of clinical research and a completed CONSORT checklist must be included with all submissions.

Clinical trial registration

NCT01958086

Study protocol

Available at clinicaltrials.gov under the above registration numbers.

Data collection

All data were collected at Casa Colina Hospital and Centers for Healthcare. Data were collected in 1–2 hour recording sessions, which occurred over 2 months for Participant JJ and 1 year for Participant RD.

Outcomes

We pre-specified outcomes at two levels. Primary outcome measures quantified condition-dependent neural activity during movement: (i) single-neuron firing-rate modulation, assessed by comparing trial-averaged firing rates between movement conditions within a pre-defined mid-movement analysis window and testing for significant differences across trials; and (ii) population-level divergence of latent dynamics, assessed by fitting a linear dynamical system (LDS) to the recorded neural population and comparing inferred latent trajectories across conditions within the same pre-defined window. Secondary outcome measures evaluated model generalization and whether condition differences could be explained by switching dynamics: (i) cross-condition generalization, assessed by training an LDS on one condition and quantifying predictive/generative performance on the other; and (ii) discrete-state structure, assessed by fitting recurrent switching LDS (rSLDS) models with varying numbers of discrete states and selecting the minimal state count that improved fit without overfitting, and by verifying that inferred states aligned with pre-defined transient and steady movement phases.

Plants

Seed stocks

Report on the source of all seed stocks or other plant material used. If applicable, state the seed stock centre and catalogue number. If plant specimens were collected from the field, describe the collection location, date and sampling procedures.

Novel plant genotypes

Describe the methods by which all novel plant genotypes were produced. This includes those generated by transgenic approaches, gene editing, chemical/radiation-based mutagenesis and hybridization. For transgenic lines, describe the transformation method, the number of independent lines analyzed and the generation upon which experiments were performed. For gene-edited lines, describe the editor used, the endogenous sequence targeted for editing, the targeting guide RNA sequence (if applicable) and how the editor was applied.

Authentication

Describe any authentication procedures for each seed stock used or novel genotype generated. Describe any experiments used to assess the effect of a mutation and, where applicable, how potential secondary effects (e.g. second site T-DNA insertions, mosiacism, off-target gene editing) were examined.
